# Transcriptomics and metabolomics reveal the underlying mechanism of drought treatment on anthocyanin accumulation in postharvest blood orange fruit

**DOI:** 10.1186/s12870-024-04868-1

**Published:** 2024-03-02

**Authors:** Hongbin Liu, Yan Jin, Le Huang, Chouyu Miao, Jiayi Tang, Huimin Zhang, Haojie Yin, Xiaopeng Lu, Na Li, Suming Dai, Alessandra Gentile, Ling Zhang, Ling Sheng

**Affiliations:** 1 National Center for Citrus Improvement Changsha, College of Horticulture, Hunan Agricultural University, Changsha, 410128 China; 2 Department of Agriculture and Food Science, University of Catania, Catania, 95123 Italy; 3 Agriculture and Rural Bureau of Mayang Miao Autonomous County, Huaihua, China

**Keywords:** Anthocyanin, Seasonal drought, Metabolome, Transcriptome, ‘Tarocco’ blood orange, Postharvest

## Abstract

**Background:**

Anthocyanins are the most important compounds for nutritional quality and economic values of blood orange. However, there are few reports on the pre-harvest treatment accelerating the accumulation of anthocyanins in postharvest blood orange fruit. Here, we performed a comparative transcriptome and metabolomics analysis to elucidate the underlying mechanism involved in seasonal drought (SD) treatment during the fruit expansion stage on anthocyanin accumulation in postharvest ‘Tarocco’ blood orange fruit.

**Results:**

Our results showed that SD treatment slowed down the fruit enlargement and increased the sugar accumulation during the fruit development and maturation period. Obviously, under SD treatment, the accumulation of anthocyanin in blood orange fruit during postharvest storage was significantly accelerated and markedly higher than that in CK. Meanwhile, the total flavonoids and phenols content and antioxidant activity in SD treatment fruits were also sensibly increased during postharvest storage. Based on metabolome analysis, we found that substrates required for anthocyanin biosynthesis, such as amino acids and their derivatives, and phenolic acids, had significantly accumulated and were higher in SD treated mature fruits compared with that of CK. Furthermore, according to the results of the transcriptome data and weighted gene coexpression correlation network analysis (WGCNA) analysis, phenylalanine ammonia-lyase (*PAL3*) was considered a key structural gene. The qRT-PCR analysis verified that the *PAL3* was highly expressed in SD treated postharvest stored fruits, and was significantly positively correlated with the anthocyanin content. Moreover, we found that other structural genes in the anthocyanin biosynthesis pathway were also upregulated under SD treatment, as evidenced by transcriptome data and qRT-PCR analysis.

**Conclusions:**

The findings suggest that SD treatment promotes the accumulation of substrates necessary for anthocyanin biosynthesis during the fruit ripening process, and activates the expression of anthocyanin biosynthesis pathway genes during the postharvest storage period. This is especially true for *PAL3*, which co-contributed to the rapid accumulation of anthocyanin. The present study provides a theoretical basis for the postharvest quality control and water-saving utilization of blood orange fruit.

**Supplementary Information:**

The online version contains supplementary material available at 10.1186/s12870-024-04868-1.

## Background

Blood oranges are rich in anthocyanins, which are responsible for the red color of the fruit and also exhibit a potent antioxidant activity with benefits for human health [1, 2], and are therefore favored by consumers. In fact, the blood orange is the only commercial citrus variety that can accumulate anthocyanins in its fruit. In production, blood orange fruit matures from February to March, and the fruit is prone to freezing damage when hanging on trees for overwintering. Therefore, the fruit is usually picked in December, which means that the low accumulation of anthocyanins and poor flesh coloring at this time seriously affect its commercial value and market competitiveness. Generally, the accumulation of anthocyanins in blood orange fruit usually requires postharvest cold storage [2, 3]. As is well known, prolonged postharvest storage can lead to a decrease in fruit flavor and quality, and long-term low temperature storage can also cause chilling injury to the fruit [4]. Thus, finding efficient and safe methods to accelerate the accumulation of anthocyanins in postharvest blood orange fruit is of great significance for the fresh fruit market and also for the industry for juice processing.

In fact, soil plastic film mulch, a form of regulated deficit irrigation (RDI) used to control soil moisture, is commonly employed in citrus production regions to improve fruit quality. This includes plastic film covering during fruit expansion period [5, 6] and pre-harvest stage [7]. A few studies about the RDI during citrus fruit expansion period (summer to autumn) have mainly focused on sugar and acid [6, 8]. Indeed, researches has shown that RDI treatment during the fruit growth can improve the red skin and increase the anthocyanin content in mature peach fruit [9]. Meanwhile, many studies have shown that the RDI strategy was used during the growing stage of grape to improve red color development, by inducing the expression of genes involved in three major pathways that control the red color in grapes: anthocyanin biosynthesis, hormone biosynthesis, and the antioxidant system [10, 11]. However, there are examples of studies that have shown no significant changes in anthocyanin levels under water deficit, so this response, although common, is not universal [12, 13]. Therefore, it is unclear whether drought treatment during the expansion period of blood orange fruit will affect the accumulation of anthocyanin during storage.

The genes involved in anthocyanin biosynthesis pathway have been extensively reported, including phenylalanine ammonia-lyase (PAL), chalcone synthase (CHS), chalcone isomerase (CHI), flavanone 3-hydroxylase (F3H), flavonoid 3’-hydroxylase (F3’H), dihydroflavonol 4-reductase (DFR), anthocyanidin synthase (ANS), uridine diphosphate-glucose:flavonoid 3-O-glucosyltransferase (UFGT) and glutathione S transferase (GST) [14, 15]. And the regulatory related genes for the anthocyanin biosynthesis are MYB, bHLH and WD40 [16–18]. Research has shown that UV-A irradiation induced significant accumulations of anthocyanin in both the cotyledons and hypocotyls of tomato seedlings through regulation of *PAL* gene expression [19]. Exogenous ABA treatment can accelerate the increase of anthocyanin and phenolic contents and PAL activity during strawberry storage [20]. Melatonin treatment could promote the accumulation of total phenols, flavonoids and anthocyanins through upregulating the expression of genes related to phenylpropane metabolism in postharvest stored blueberry fruit [21]. Cooler temperatures during ripening promote the accumulation of phenolics, especially anthocyanins in blueberries, which may be attributed to the up-regulation of flavonoid biosynthetic genes at cooler temperatures [22]. Early season water deficits accelerated sugar accumulation, and increased anthocyanin accumulation in grape berries through upregulating the expression of genes in the anthocyanin biosynthetic pathway including *F3H*, *DFR*, *UFGT* and *GST* [23]. It can be seen that the main genes responsible for accumulating anthocyanin under different environmental stresses or treatments are different.

Generally, citrus production areas in China have sufficient annual rainfall, but are mismatching spatial-temporal distribution. High temperature and low rainfall from late July to early September (the fruit expansion period) occur universally in most citrus production areas, which are referred as “summer-autumn seasonal drought” or “seasonal drought” (SD). Our previous research has shown that SD for 30 d was an important time node that influence the fruit quality of ‘Bingtangcheng’ sweet orange. Hence, this study simulates SD through film covering for 30 d to explore its impact and potential mechanism on blood oranges fruit quality. Our results provide a basis for fruit quality control of blood orange.

## Results

### The influences of drought treatment on phenotype and fruit quality during ripening and postharvest blood orange

**Fig. 1:**
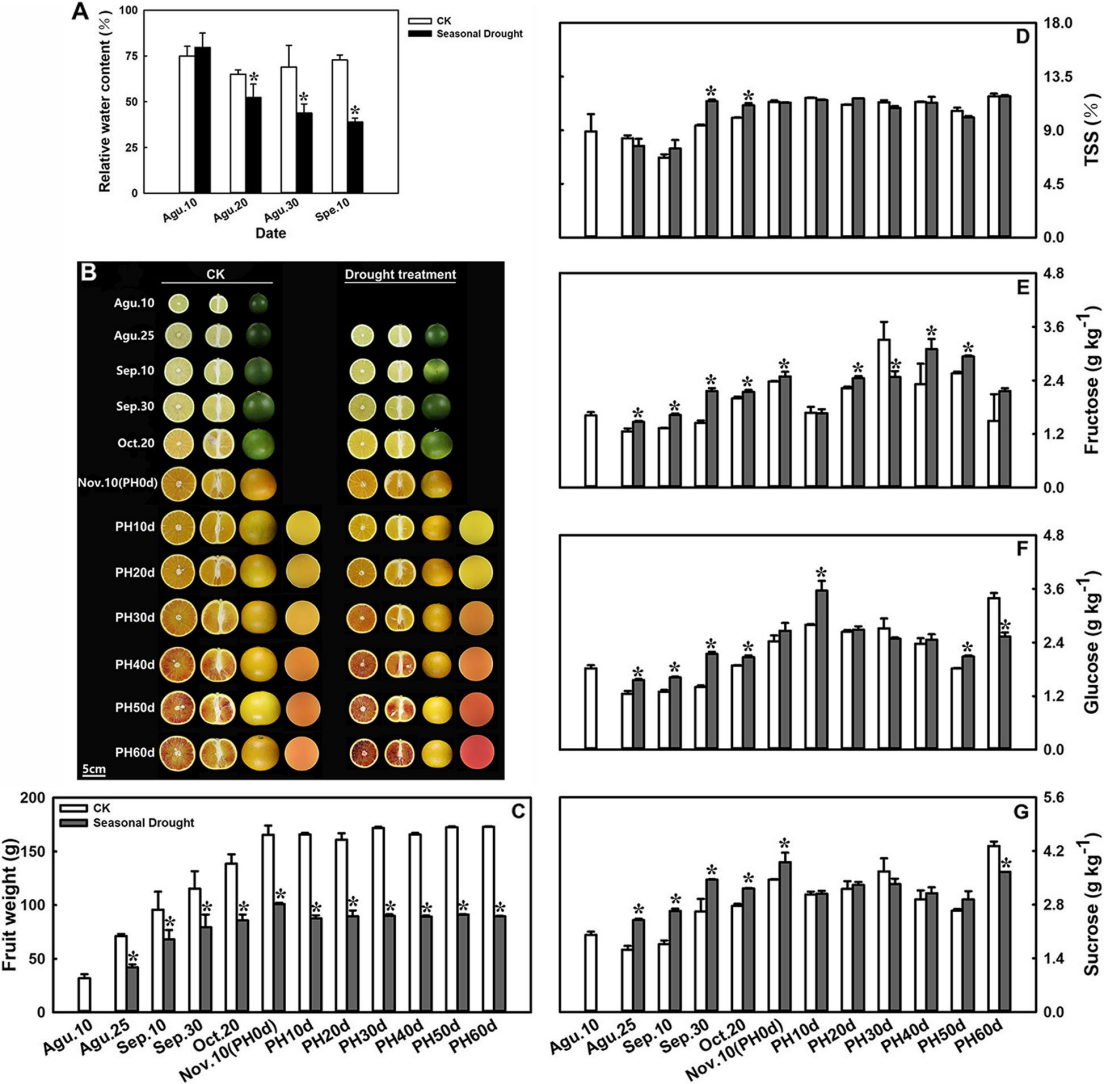
Effects of drought treatment on the phenotype and fruit quality during fruit ripening and postharvest storage period. **A **Relative water content of soil. **B **Fruit phenotype. The fourth column is picture of the juice. **C **Fruit weight. **D **Total soluble solid (TSS) content. **E **Fructose content. **F **Glucose content. **G **Sucrose content. Error bars represent the standard deviations of the mean in three replicates, and for each stage, * stand for significant differences between seasonal drought treatment and control at *p* < 0.05

The results showed that the seasonal drought (SD) treatment significantly reduced the soil moisture content (Fig. 1A). Before treatment, the soil relative water content of the control (CK) and treatment groups was 74.99 ± 5.36% and 79.63 ± 7.86% respectively, and with no significant difference. However, after 30 d of drought treatment, the soil relative water content of the CK group was maintained 72.95 ± 2.75%, but the drought treatment group decreased to 38.97 ± 2.10%, which was significantly lower than that of the CK. Notably, SD treatment slows down the fruit enlargement in the fruit development process, resulting in significantly smaller fruit weight (Fig. 1C). Interestingly, SD treatment significantly accelerates the accumulation of anthocyanin in the flesh of the fruit during postharvest storage (Fig. 1B). Quality analysis results indicated that SD did not significantly affect total organic acid (TOA) content during fruit ripening and postharvest storage (Fig. S1A). The total soluble solid (TSS) content significantly increased during fruit ripening under the treatment (Fig. 1D). Moreover, SD increased the ascorbic acid content and decreased the juice yield overall (Fig. S1D and E).

The results indicated that the content of citric acid was not affected by SD treatment, while the content of malic acid increased at some points due to the treatment (Supplementary Fig. S1B and C). However, the sugar content was greatly affected by SD treatment. In Fig. 1E-G, it can be seen that the accumulation of fructose, glucose, and sucrose content in the treated fruit was significantly higher than those in the control during fruit ripening. Additionally, during postharvest storage, the fructose and glucose contents were significantly higher in the treated fruit than those in control at postharvest (PH) 20d, 40d, 50d and 10d, 50d, respectively. These results demonstrated that SD can significantly promote the accumulation of the substrate sugars during fruit ripening, which is beneficial for anthocyanin biosynthesis during postharvest storage.

### Effects of drought treatment on fruit peel and juice color index and anthocyanin content in postharvest blood orange fruit

**Fig. 2:**
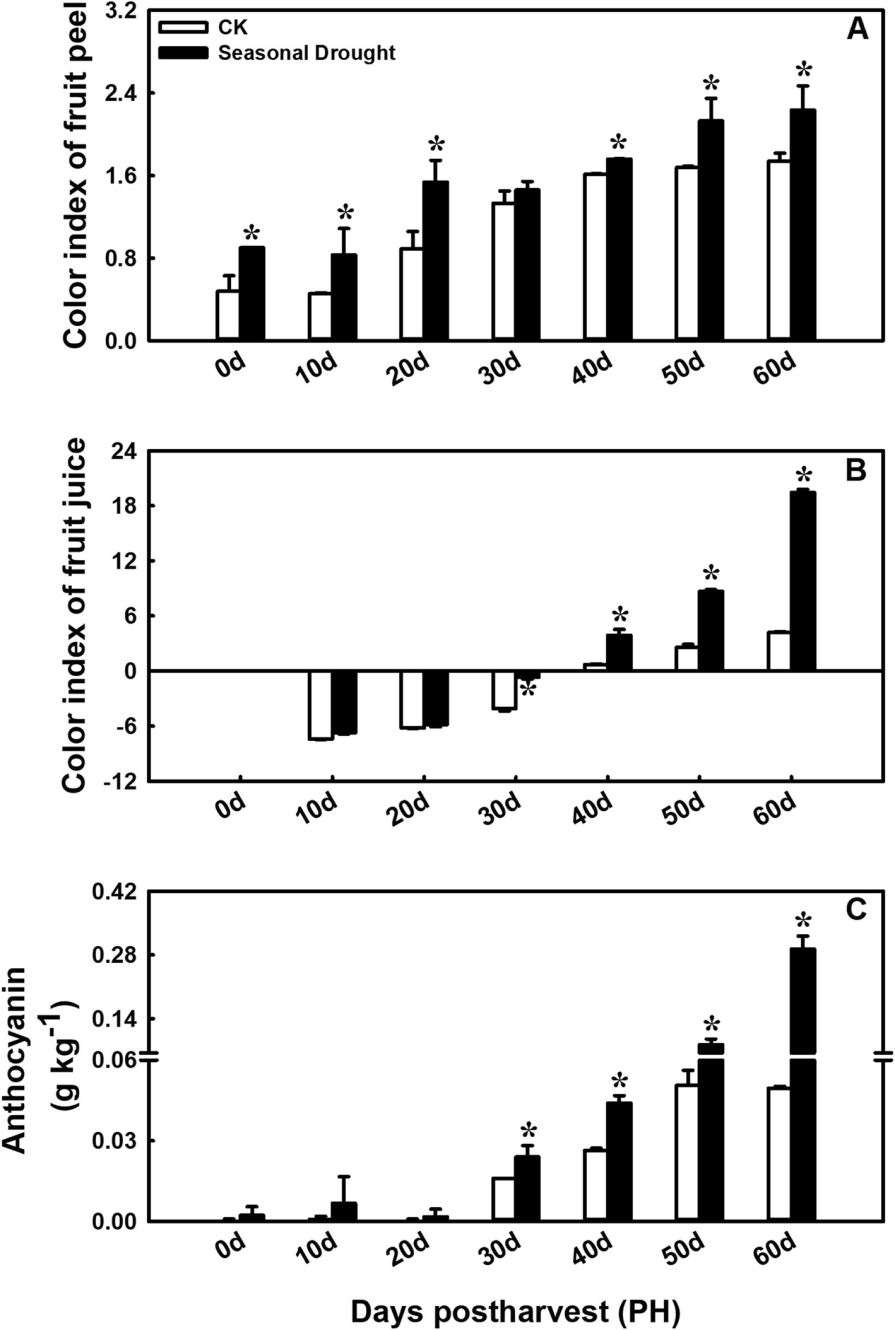
Effects of drought treatment on color index of fruit peel (**A**) and juice (**B**), and anthocyanin content (**C**) in postharvest stored blood orange fruit. Error bars represent the standard deviations of the mean in three replicates, and for each stage, * stand for significant differences between seasonal drought treatment and control at *p* < 0.05

The color index of both fruit peel and juice, as well as the anthocyanin content, demonstrates a gradual upward trend throughout the storage period. Under SD treatment, the color index of fruit peel was significantly higher than that of the control for almost the entire storage period (Fig. 2A). Compared to the control fruit, the treated fruit exhibited a more orange-red skin color. However, the color index of the fruit juice in the treatment group was significantly different from that of the control starting from PH30d, and was significantly higher than that of the control (Fig. 2B). A similar trend was observed in the anthocyanin content; it was significantly higher in the treated fruit compared to that in the control fruit from PH30d to PH60d (Fig. 2C). Particularly at PH60d, the anthocyanin content in treated fruit reached 0.2930 ± 0.0283 g kg^-1^ fresh fruit, which was nearly 6 times higher than that in the control fruit (0.0495 ± 0.0007 g kg^-1^). These results suggest that SD treatment could enhance the nutritional value of blood orange fruit by accelerating anthocyanin accumulation during postharvest storage, consistent with the observed phenotype.

### Effects of drought treatment on total flavonoid and phenol content, and antioxidant activity in postharvest blood orange fruit

**Fig. 3:**
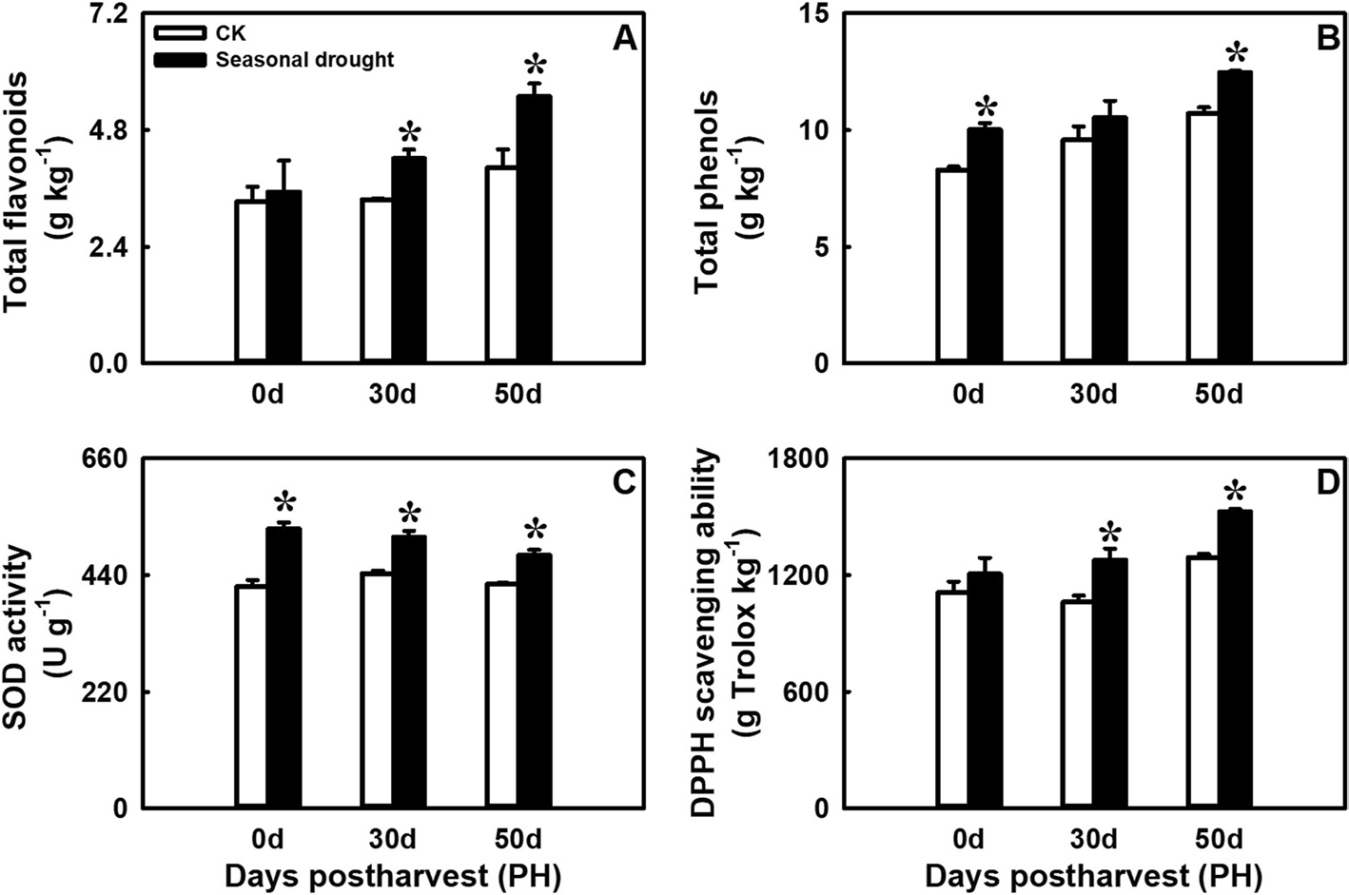
Effects of seasonal drought treatment on total flavonoids content (**A**), total phenols content (**B**), SOD activity (**C**) and DPPH scavenging ability (**D**) during postharvest storage. Error bars represent the standard deviations of the mean in three replicates, and for each stage, * stand for significant differences between seasonal drought treatment and control at *p* < 0.05

The content of total flavonoids and phenols exhibited an overall upward trend during postharvest storage, and the total flavonoid content in SD treated fruit was significantly higher than that in the control at PH30d and PH50d (Fig. 3A). Meanwhile, the total phenol content was significantly higher at PH0d and PH50d (Fig. 3B). Consequently, fruit treated with SD also exhibited notably higher superoxide dismutase (SOD) enzyme activity and 1,1-Diphenyl-2-picrylhydrazyl radical 2,2-Diphenyl-1-(2,4,6-trinitrophenyl)hydrazyl (DPPH) free radical scavenging ability during postharvest storage (Fig. 3C and D). These results indicated that SD treatment can significantly enhance the accumulation of secondary metabolites and improve the fruit’s antioxidant capacity.

### Differentially accumulated metabolites in blood orange fruit upon drought treatment through widely targeted metabolome analysis

We speculate that SD treatment activated the metabolic pathways and promoted the accumulation of metabolites related to anthocyanin biosynthesis during fruit ripening. Therefore, a widely targeted metabolomics technique was utilized to systematically identify and quantify all metabolites present in the SD-treated and control fruit at the point of fruit maturity (at PH0d). PCA results showed that samples were scattered between the control (CK) and the SD treatment (SD) groups, while the samples within each group were closely clustered (Supplementary Fig. S2A), suggesting that the experiment was reproducible and reliable.

**Fig. 4:**
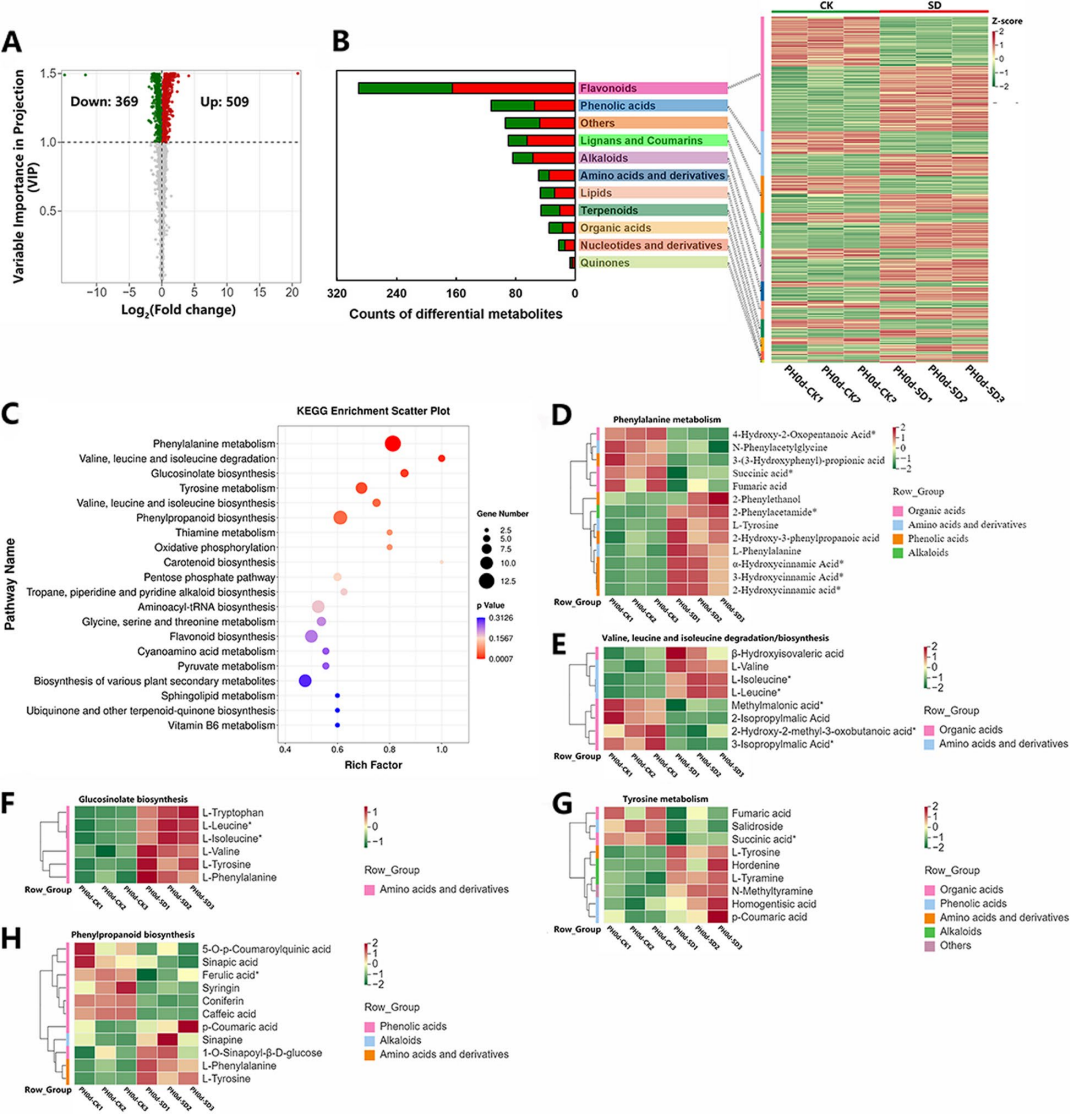
Analysis of differentially accumulated metabolites in juice sacs of blood orange fruit under drought treatment compared with control. **A **Volcano map of DAMs. **B** Biochemical categories of DAMs and their hierarchical clustering. **C** Scatter diagram of pathway enrichment of DAMs. **D**-**H** Clustering of DAMs belonging to various KEGG pathway; **D** phenylalanine metabolism; **E** valine, leucine and isoluecine degradation/biosynthesis; **F** glucosinolate biosynthesis; **G** tyrosine metabolism; **H** phenylpropanoid biosynthesis. The color indicates the accumulation level of each metabolite, from low (green) to high (red). The data was standardized by Z-score. “PH0d-CK” and “PH0d-SD” means samples from control and drought treatment blood orange fruit at mature stage, respectively; All of these DAMs were listed in Table S2

A total of 1846 metabolites were identified and categorized into 11 groups (Supplementary Fig. S2B). Further cluster analysis indicated that the majority of metabolites differed significantly between the control (CK) and the SD groups, with the biological replicates of the samples clustering together (Supplementary Fig. S2C). This indicated that the metabolome data are reliable and suitable for subsequent quantitative analyses. A further 878 differentially accumulated metabolites (DAMs) were identified, with 509 being up-regulated and 369 down-regulated (Fig. 4A). These DAMs were classified into 11 categories, with the largest group being flavonoids (33.18%, 291/878), followed by phenolic acids (12.88%, 113/878), and then by others (10.72%, 94/878) lignans and coumarins (10.26%, 90/878). Interestingly, the majority of lignans and coumarins (approximately 72.22%, 65/90) were up-regulated in the SD-treated fruit (Fig. 4B, Table S2), indicating that lignans and coumarins contribute to postharvest anthocyanin accumulation.

To reveal which metabolic pathways were involved in the changes of the metabolites in SD treatment fruit, we employed Kyoto Encyclopedia of Genes and Genomes (KEGG) analysis to annotate these metabolites and clarify the active metabolic pathways. The results showed that a total of six pathways were significantly regulated under SD treatment, including “phenylalanine metabolism”, “valine, leucine and isoluecine degradation”, “glucosinolate biosynthesis”, “tyrosine metabolism”, “valine, leucine and isoluecine biosynthesis” and “phenylpropanoid biosynthesis” (Fig. 4C). This indicates that these pathways may be altered under SD treatment.

All the DAMs identified in the six most significantly enriched metabolic pathways were further analyzed. A total of 13 DAMs were annotated to the ‘phenylalanine metabolism’ pathway. Notably, the phenylalanine content increased by 28.31% in SD-treated fruit (Fig. 4D, Table S2). Additionally, 8 DAMs were annotated to the “valine, leucine and isoluecine degradation/biosynthesis” pathway. The content of valine, leucine and isoluecine increased by 32.62%, 42.28% and 42.28%, respectively, under SD treatment (Fig. 4E, Table S2). Furthermore, 6 DAMs related to “glucosinolate biosynthesis”, which were all amino acids and derivatives and were upregulated. Pronounced changes were observed in the levels of tryptophan and tyrosine, which increased by 2.17- and 1.57-fold, respectively (Fig. 4F, Table S2). In the “tyrosine metabolism” pathway, 9 DAMs were annotated (Fig. 4G, Table S2). In the “phenylpropanoid biosynthesis” pathway, 11 DAMs were annotated, and 8 were phenolic acids. Notably, the content of p-coumaric acid increased by 25.90%, while the contents of caffeic acid, ferulic acid, and p-coumaroylquinic acid decreased by 34.40%, 17.22%, and 12.84%, respectively (Fig. 4H, Table S2). The data suggested an accumulation of metabolic substrates in anthocyanin biosynthesis, such as amino acids and derivatives (like phenylalanine, tryptophan, tyrosine, valine, leucine and isoleucine) and phenolic acids (like p-coumaric acid), accompanied by a decrease of metabolites in the anthocyanin biosynthesis branching pathway, such as phenolic acids (like caffeic acid, ferulic acid, and p-coumaroylquinic acid), which occurred in SD-treated fruit. This indicates that the upstream substrates for anthocyanin biosynthesis had already largely accumulated due to SD treatment during the fruit ripening process.

### Identification of key genes for anthocyanin accumulation in response to drought treatment

**Fig. 5:**
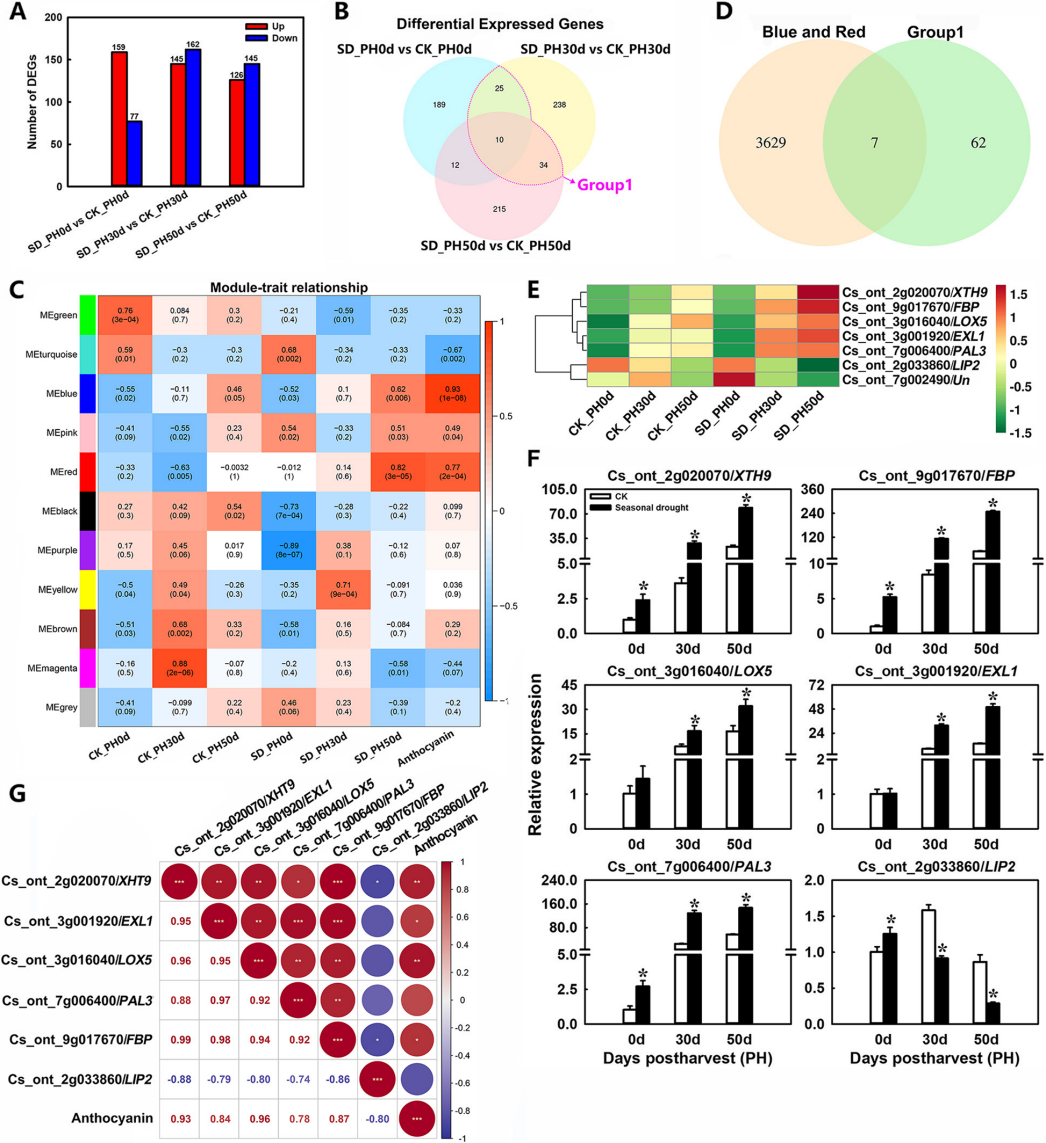
Analysis of key genes related to anthocyanin accumulation. **A** Number of DEGs in different comparison groups. **B** Venn diagram shows DEGs in drought treated fruit vs. control fruit. **C** Module/trait correlations and corresponding *p* values. The right panel shows a colour scale for module/trait correlations from − 1 to 1. **D** Venn diagram shows DEGs in group1 and the blue and red modules. **E** Heatmap of the expression levels of DEGs in the intersection genes between group1 and the blue and red modules. **F** qRT-PCR detection of intersection genes between group1 and the blue and red modules, *XTH9* (xyloglucan endotransglucosylas), *LIP2* (octanoyltransferase), *EXL1* (GDSL esterase/lipase), *LOX5* (probable linoleate 9s-lipoxygenase), *PAL3* (phenylalanine ammonia lyase) and *FBP* (F-box protein). Error bars represent the standard deviations of the mean in three replicates, and for each stage, * stand for significant differences between seasonal drought treatment and control at *p* < 0.05. **G** Correlation analysis of the expression profiles in qRT-PCR and anthocyanin content (*, ** and *** represent significant differences at *p* < 0.05, *p* < 0.01 and *p* < 0.001, respectively between the two sets of data)

To uncover the mechanism behind the impact of SD treatment on the accumulation of anthocyanin in blood orange fruit, samples from three detection time points (PH0d, PH30d, and PH50d) from the CK and SD group were used for deep RNA-seq analysis. A total of 83 million reads were mapped to the *Citrus sinensis* reference genome (Table S3). The average percentage of mapped reads per sample ranged from 93.50 to 94.45%. A total of 29,875 annotated genes were obtained. Differentially expressed genes (DEGs) were identified based on their expression levels between the CK and SD group, and functional annotation and enrichment analysis were performed. A total of 814 genes were differentially expressed between the CK and the SD group (Fig. 5A). To screen the candidate genes related to anthocyanin biosynthesis in response to SD treatment, our study focused on the DEGs in the SD vs. the CK. There were 236, 307, and 271 genes differentially expressed on PH0d, PH30d, and PH50d, respectively (Fig. 5A). Among them, 25, 34, and 10 intersection genes of PH0d + PH30d, PH30d + PH50d, and PH0d + PH30d + PH50d were identified, respectively. These 69 DEGs were classified as group 1 (Fig. 5B).

Further relationships between DEGs and anthocyanin content were explored by constructing a weighted gene coexpression correlation network analysis (WGCNA) (Fig. 5C, Supplementary Fig. S3), and fourteen coexpression modules were identified (Supplementary Fig. S3). Among these, the blue and red modules were highly positively correlated with anthocyanin contents (blue: *r* = 0.93, *p*-value = 1e-08; red: *r* = 0.77, *p*-value = 2e-04). A total of 3361 genes from the blue and 275 genes from the red module were identified. To further explore key genes involved in anthocyanin accumulation in response to SD treatment, we focused on the intersection genes between the group1 (Fig. 5B) and the blue and red modules. Ultimately, 7 valuable genes were identified (Fig. 5D), including xyloglucan endotransglucosylase (*XTH9*), octanoyltransferase (*LIP2*), GDSL esterase/lipase (*EXL1*), probable linoleate 9s-lipoxygenase (*LOX5*), phenylalanine ammonia lyase (*PAL3*), F-box protein (*FBP*), and an unknown protein (*Un*). Of these, 5 genes were upregulated and 2 genes were downregulated under SD-treatment (Fig. 5E). Further the results of quantitative real-time PCR (qRT-PCR) are consistent with transcriptome data (Fig. 5F). Notably, the structural gene *PAL3*, involved in the anthocyanin biosynthesis pathway, was identified. Its expression was significantly upregulated throughout the storage period in SD-treated fruit as shown by qRT-PCR analysis and was positively correlated with anthocyanin content (Fig. 5F and G). These results imply that the significant role of *PAL3* in SD-induced anthocyanin accumulation in postharvest blood orange fruit.

### Expression patterns of anthocyanin biosynthesis pathway genes in response to drought treatment

**Fig. 6:**
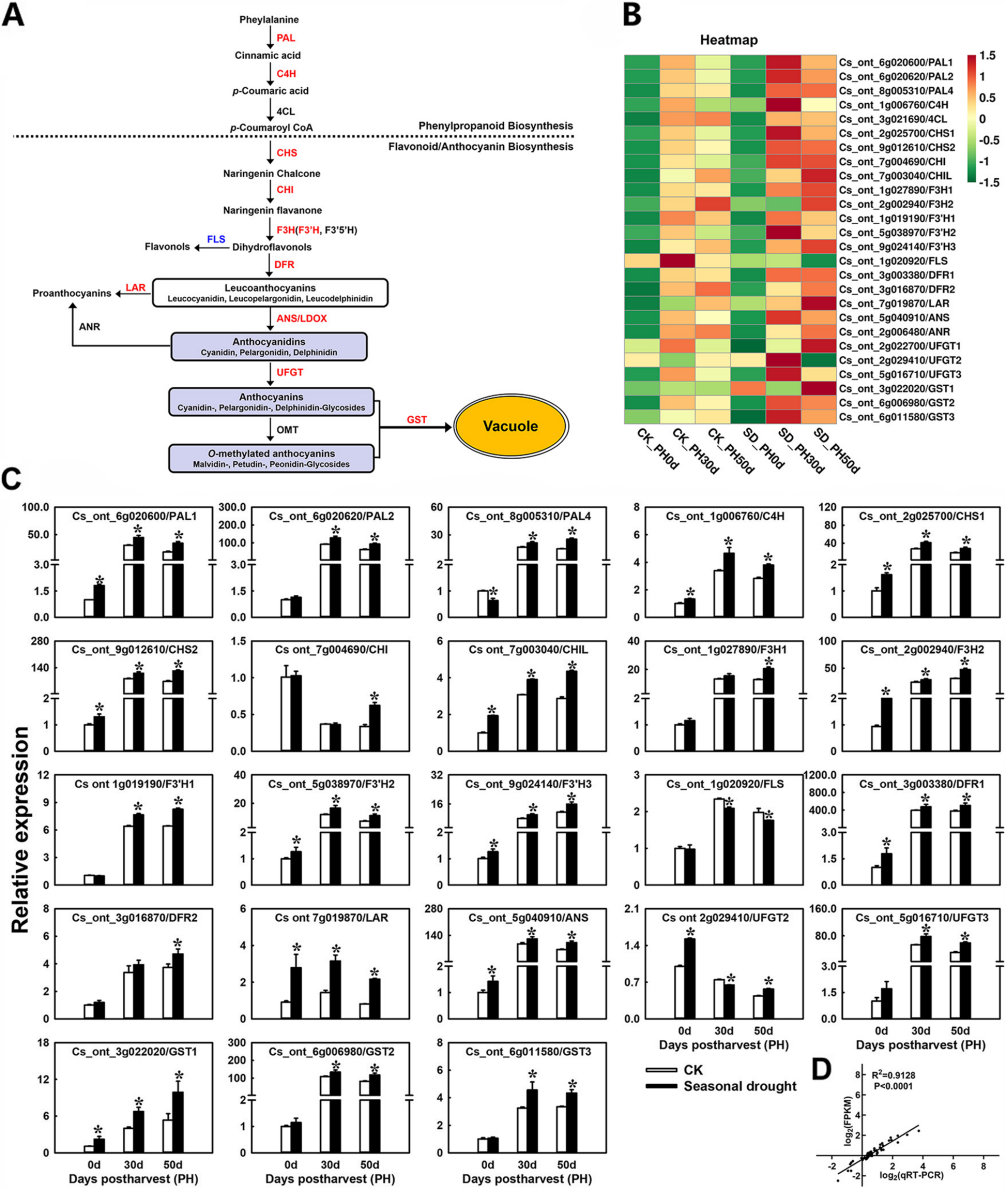
Expression patterns of anthocyanin biosynthesis pathway genes. **A** Anthocyanin biosynthesis pathway. The red and blue font indicates the genes upregulated and downregulated by drought treatment respectively, and the black font indicates that there is no difference in the expression of this gene between the treatment and the control. **B** Heatmap of the expression levels of anthocyanin biosynthesis pathway genes. **C** qRT-PCR detection of anthocyanin biosynthesis pathway genes. **D** Correlation between the RNA-seq and qRT-PCR. Error bars represent the standard deviations of the mean in three replicates, and for each stage, * stand for significant differences between seasonal drought treatment and control at *p* < 0.05

Further analysis of the expression patterns of the entire anthocyanin biosynthesis pathway genes indicated that almost all of the structural genes from the major steps of the anthocyanin biosynthesis pathway were upregulated during postharvest storage by SD-treatment (Fig. 6A and B). This included three additional *PAL* genes (*PAL1*, *PAL2*, and *PAL4*), cinnamate 4-hydroxylase (*C4H*), two chalcone synthase (*CHS1* and *CHS2*), two chalcone isomerase (*CHI* and *CHIL*), two flavanone 3-hydroxylase (*F3H1* and *F3H2*), three flavonoid 3’-hydroxylase (*F3’H1*, *F3’H2*, and *F3’H3*), two dihydroglavonol 4-reductase (*DFR1* and *DFR2*), anthocyanidin synthase (*ANS*), two uridine diphosphate-glucose:flavonoid 3-O-glucosyltransferase (*UFGT2* and *UFGT3*), and three glutathione-S-transferases (*GST1*, *GST2*, and *GST3*). Moreover, we observed that the transcript level of flavonol synthase gene (*FLS*) was significantly downregulated following SD-treatment. Additionally, the expression patterns of these structural genes were confirmed via qRT-PCR analysis (Fig. 6C), which was consistent with the results of transcriptome analysis. Linear regression analysis revealed a correlation coefficient of 0.9128 between RNA-seq and qRT-PCR data (Fig. 6D), indicating the reliability and reproducibility of the RNA-seq data in our study.

## Discussion

### Changes in fruit quality of blood orange under drought treatment

The most distinctive feature of blood orange fruit is its ability to accumulate anthocyanins, while other flavor qualities such as sugars and acids are not as prominent. Consequently, the anthocyanin content directly influences its commercial value. The noticeable visual changes in blood orange fruit under SD treatment included smaller fruit size and redder flesh (Fig. 1). Research has showed that RDI conditions in citrus may lead to smaller fruit size [24, 25]. But the variations in fruit weight at any stage of development can depend on the species and the specific water restriction conditions [26]. Significantly, anthocyanin accumulation accelerated and was markedly higher in treated fruit than in control fruit from PH30d to PH60d (Fig. 2C), consistent with the observed phenotype (Fig. 1B). This result corroborates the previous reports that RDI during fruit development period can enhance fruit quality by increasing the anthocyanin content in mature grape and peach fruit [9–11]. Therefore, fruit under SD-treatment began to significantly accumulate anthocyanins (0.0240 ± 0.0042 g kg^-1^) at PH30d (on December 10th), and reached 0.2930 ± 0.0283 g kg^-1^ at PH60d (on January 10th). At this time, the ripening was still far ahead of the normal maturity period (February to March), thereby enhancing the unique market value of blood orange.

Some studies have shown that similar drought treatments can significantly increase the sugars, acids, and ascorbic acid content in mature citrus fruit [6, 8]. In this study, a significant increase in TSS, fructose, glucose, sucrose, and ascorbic acid content was observed in blood orange fruit during the ripening process under SD-treatment, although the TOA and citric acid content did not change significantly (Fig. 1, Supplementary Fig. S1). This variation may be attributed to inherent differences between varieties, as blood oranges typically have a higher acid content compared to other citrus cultivated varieties. A previous study observed that total acidity, ascorbic acid, glucose, and fructose were not affected by DI treatments in pomegranate [27]. Additionally, several studies have demonstrated that anthocyanin content is closely related to the antioxidant activity of the fruit [28, 29]. Prior research indicated that sustained DI during the fruit-growing season could increase the total phenolic content in the leaves and fruit, as well as the anthocyanin content in apples [30]. Furthermore, the total phenol content, antioxidant activity, and anthocyanins content in pomegranate juices were higher in samples subjected to the DI treatments compared to those receiving full irrigation [27]. In this study, an increase in total flavonoids, total phenols, SOD activity, and DPPH scavenging ability was found in SD-treated fruit during postharvest storage (Fig. 3). These results suggest that SD-treatment could enhance the quality of blood orange in multiple ways.

### Drought treatment promotes sugars and substrates accumulation and gene expression required for anthocyanin biosynthesis in postharvest blood orange fruit

Both phenotypic and physiological indicators confirm that SD treatment can indeed accelerate the accumulation of anthocyanin in postharvest blood oranges. To elucidate the underlying mechanism of SD treatment during fruit enlargement and its impact on the accumulation of anthocyanin during postharvest storage, transcriptome and metabolome analyses were then employed. The plant metabolome has been extensively applied to investigate patterns of metabolite accumulation, and has been utilized to discern the changes in genes and metabolites profiles across various tissues [10, 31, 32]. In *Cabernet Sauvignon* grape berries, transcriptomics and metabolomics studies have shown that the potential mechanisms by RDI treatment affects anthocyanin accumulation in mature fruit are due to the upregulation of key genes in the anthocyanin biosynthetic pathway, such as *VvPAL*, *VvC4H*, *Vv4CL*, *VvCHS*, *VvF3’H*, *VvF3’5’H*, *VvLDOX*, *VvUFGT*, and *VvOMT*, and the significantly in metabolites within this pathway, including cinnamic acid, naringenin chalcone, naringenin and eriodictyol [10, 33]. Moreover, a targeted metabolomic approach and transcriptome analysis have revealed a significant increase in the content of anthocyanin-related metabolites, including pelargonidin, cyanidin, and peonidin. Additionally, there was a marked increase in the expression levels of anthocyanin biosynthesis genes such as *CHS*, *CHI*, *F3H*, *DFR*, *ANS* and *UFGT* during fruit peel development. These factors collectively contribute to the color development of *Cerasus humilis* [31]. In the current study, a widely targeted metabolomic analysis was employed to identify the DAMs in mature blood orange fruit subjected to SD-treatment. Notably, among these DAMs, we observed a significant accumulation of metabolic substrates in anthocyanin biosynthesis, such as amino acids and their derivatives (like phenylalanine, tryptophan, tyrosine, valine, leucine, and isoluecine), and phenolic acids (like p-coumaric acid). This was paired with a decrease in metabolites within the anthocyanin biosynthesis branching pathway, such as phenolic acids (like caffeic acid, ferulic acid, and p-coumaroylquinic acid) (Fig. 4D-H). These results suggest that SD-treatment promotes the accumulation of substrates necessary for anthocyanin biosynthesis during fruit ripening process, which could be one reason why the accumulation of anthocyanins in SD-treated blood orange fruit occurs earlier than in control fruit during postharvest storage.

The anthocyanin metabolism pathway has been largely elucidated in model plants based on previous studies, and significant progress has been made in researching genes involved in anthocyanin biosynthesis in fruit crops [34, 35]. In recent years, transcriptomic analysis has emerged as a crucial method for identifying key genes that regulate phenotype characteristics [2, 35]. This has enabled researchers to gather valuable information about the accelerated accumulation of anthocyanin in SD-treated blood orange fruit during postharvest storage. In this study, differential and upregulated expression of almost all structural genes including *PAL*, *C4H*, *CHS*, *CHI*, *F3H*, *F3’H*, *DFR*, *ANS*, *UFGT*, and *GST* was observed, as evidenced by transcriptome data and qRT-PCR. These genes were mapped to the entire anthocyanin biosynthesis pathway (Fig. 6), with particular emphasis on *PAL3* (Fig. 5). In non-bagged and shaded apples, a positive linear relationship was noted between maximum PAL activity and anthocyanin accumulation [36]. Furthermore, correlation analysis suggested that *CsPAL4* is closely associated with various anthocyanin accumulations in purple-leaf tea [37]. In most cases, the accumulation of anthocyanins and the corresponding phenotype in crops are determined by the upregulation of numerous genes in the anthocyanin biosynthesis pathway, along with related regulatory factors. A notable decrease in anthocyanins accumulation in Yunyan 87 mutant tobacco was attributed to the coordinated downregulation of anthocyanin biosynthetic genes including *CHI*, *F3H*, *DFR*, *UFGT*, as well as several genes encoding MYB and bHLH transcription factors [38]. Thirteen anthocyanin biosynthetic-related genes were upregulated in a deep pink-colored mutant rose, contributing to higher anthocyanin levels in the mutant [39]. Additionally, ABA increases the accumulation of anthocyanins in developing *Fragaria chiloensis* fruit by activating the expression of *PAL*, *CHS*, and *ANS* [40]. In our current study, the hub genes identified, which probably participated in SD-driven anthocyanin accumulation during postharvest in blood orange, were primarily structural genes (Fig. 5). Notably, we observed that the expression levels of *FBP* (Cs_ont_9g017670) and *XTH9* (Cs_ont_2g020070) gene were significantly higher in treated fruit than in control fruit throughout the storage period (Fig. 5F). This expression was significantly correlated with *PAL3* and anthocyanin content. Studies have shown that FBPs could improve drought tolerance in transgenic tobacco by maintaining higher antioxidative content [41]. However, some studies have demonstrated that F-box proteins containing kelch repeats (FBKs) negatively regulate anthocyanin accumulation by degrading PAL isoenzymes or through other unclear mechanisms [42]. Therefore, the role of these proteins in anthocyanin accumulation in SD-treated blood orange fruit remains unclear and warrants further investigation. Additionally, research has shown that *XTHs* are downregulated under drought stress and upregulated upon rewatering. This is accompanied by a significant increase in anthocyanin in drought treated plants [43]. However, there is no direct correlation between *XTHs* expression and anthocyanin content. The expression of *XTHs* genes and the accumulation of anthocyanin content appear to be distinct responses to drought stress.

**Fig. 7:**
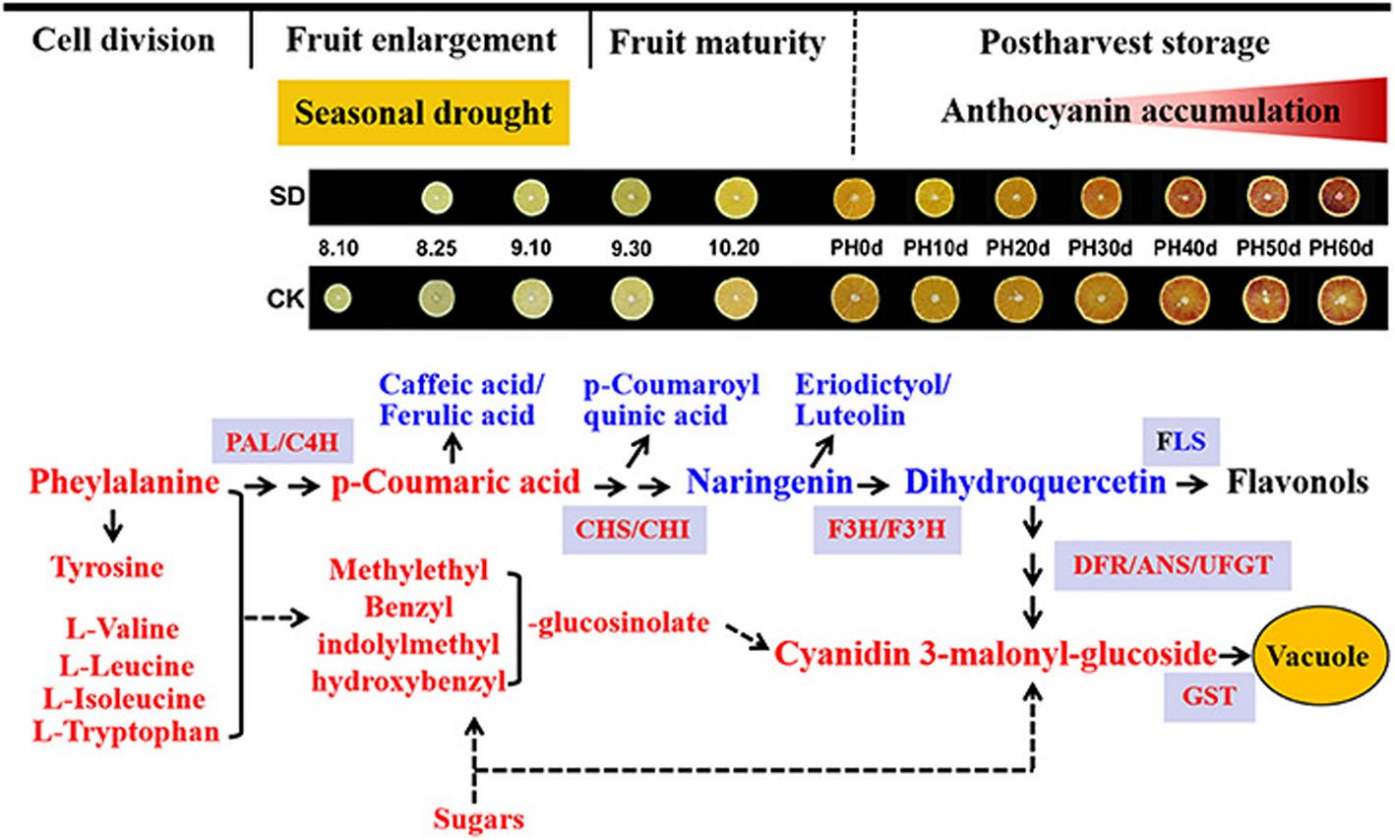
Sketch of the potential mechanisms underlying the anthocyanin accumulation in drought treated blood orange fruit compared with that in control fruit. The red typeface indicates that the metabolite content or gene expression level is significantly higher in seasonal drought treated blood orange fruit than control fruit. The blue typeface is the opposite

In addition, numerous studies have shown that anthocyanin biosynthesis could be positively affected by sugar accumulation, leading to an increase in red coloration in plant tissues [44–46]. At the molecular level, anthocyanins can be induced by a sugar signaling network [47]. Sucrose can positively modulate the expression of anthocyanin biosynthesis genes and regulators in grape berry skin and Arabidopsis [48, 49]. Glucose could activate a hexokinase (HXK)-dependent pathway, which regulates the expression of specific genes including *CHS* and *PAL1* in Arabidopsis [50]. Therefore, the sugars accumulation in blood orange during development and postharvest process under SD treatment likely induced the anthocyanin biosynthesis making the pulp more reddish than that of control. Overall, the activation of the anthocyanin biosynthesis pathway may partially explain the accelerated accumulation of anthocyanins in blood orange fruit during postharvest storage under SD-treatment. Based on the findings of previous studies [2, 3] and this study, we have developed a model to elucidate the accelerated accumulation of anthocyanin in SD-treated blood orange fruit during postharvest storage (Fig. 7).

## Conclusion

Our multi-omics approach, in combination with physiological and biochemical assays, has shown that SD-treatment enhances the contents of anthocyanin, substrates in the anthocyanin biosynthesis pathway, sugars (fructose, glucose, and sucrose), total flavonoids, total phenols, and the activity of SOD and DPPH scavenging ability in postharvest blood orange fruit. Importantly, this increase in anthocyanin is co-led by the accumulation of necessary substrates for anthocyanin biosynthesis during the fruit ripening process and the upregulation of structural genes, especially *PAL3*, in the anthocyanin biosynthesis pathway. Future research will focus on exploring the mechanism of key genes involved in anthocyanin accumulation in response to drought, and provide an application reference for postharvest quality control and water-saving utilization of blood orange fruit.

## Materials and methods

### Plant materials and treatments

Seven-year-old ‘Tarocco’ blood orange (*Citrus sinensis* [L.] Osbeck ‘Tarocco’) trees from an cooperative orchard of Agriculture and Rural Bureau of Mayang Miao Autonomous County (Mayang city, Hunan province, China) were used in the study. Twenty uniform trees were selected, with ten as the control and ten treated with drought for 30 days. The orchard ground of selected trees undergoing drought treatment was covered with plastic film depending on rainfall. The control trees (CK) were watered every three to five days to maintain constant soil moisture, as referred to in our previously study. For drought treatment, rehydration immediately synchronized with CK after the end of the drought until October 10th, after which there is no irrigation and relying on natural rainfall until the fruit ripens [51]. The drought treatment was carried out on August 10th (Aug.10, fruit enlargement stage), 2021. The detection of relative soil water content was referred to a previous study [52], and is defined as soil field water-holding capacity divided by soil maximum water-holding capacity.

During the fruit development stage: at Aug.10 and Aug.25, September 10th and 30th (Sep.10 and Sep.30), October 20th (Oct.20), 30 fruits were randomly selected from the crown periphery of the treatment and CK group for the experiment, with three replicates. When the fruit matured on November 10th (Nov.10), all the fruit on the treatment and CK trees are picked and immediately transported to the laboratory for a storage experiment. Fruit with uniform size and color and free of any visible damage or defects were selected as samples for further experiments. The fruit were individually packed in polyethylene bags and stored in a temperature-controlled chamber at 8 ± 1 °C with a relative humidity of 85-90% for about two months.

Juice sacs were separated from 10 fruits (three replicates, a total of 30 fruits) in each group at Aug.10, Aug.25, Sep.10, Sep.30, Oct.20, Nov.10 (0 day postharvest, PH0d), PH10d, PH20d, PH30d, PH40d, PH50d, and PH60d to measure the levels of organic acids, sugars, anthocyanins, total flavonoids, total phenols, antioxidant enzyme activity, and for transcriptome and metabolomics analysis, and expression analysis of related genes. The samples were immediately frozen in liquid nitrogen and stored at -80 °C for further analysis. The results are expressed on a fresh weight (FW) basis throughout the paper.

### Quality parameter determination

The fruit were weighed using an electronic balance (Model: PB2002-N, Mettler-Toledo Instrument Ltd., Shanghai, China). Total organic acid (TOA, %) and total soluble solids (TSS, %) were determined with a refractometer (Model: PAL-BX/ACIDF5, Atago Inc., Tokyo, Japan) according to the manufacturer’s instructions. The ascorbic acid content was measured by the 2,6-dichloroindophenol titrimetric method, referring to a previous study [53]. The results were expressed as g ascorbic acid per L of fruit juice (g L^-1^). Juice yield (%) was expressed as the percentage of juice weight to fruit weight. Fruit surface and juice color were measured as previously described [53]. Three readings were taken at different points using a Chroma Meter CR-400 Spectrophotometer (Japan), which provides CIE L*, a*, and b* values. The color index (CI) was calculated by the equation: CI = 1000× *a* /( *L× b)*.

### Determination of sugars and organic acids

Sugars (glucose, fructose, and sucrose) and organic acids (citric acid and malic acid) were measured and analyzed by high-performance liquid chromatography (HPLC) following a method previously described in [54]. The results were expressed based on a fresh weight (FW) basis.

### Determination of anthocyanin

The determination of anthocyanin content was conducted using the spectrophotometer method, referencing our previously described method [2] the anthocyanin content (g kg^-1^ FW) = (A510 at pH1.0 – A510 at pH4.5) × 484.8 (molecular weight of cyanidin) / 24,825 (molar absorption coefficient of cyanidin at A510) × dilution ratio, and was expressed as g kg^-1^ FW.

### Determination of total flavonoids and total phenols

Total flavonoids were determined by the spectrophotometry method referred to in a previously study [35], with minor modifications. Briefly, a 0.5 mL methanol (80%) extract was mixed with 5 mL ethanol (30%) and 0.4 mL NaNO_2_ (5%), shaken well and allowed to stand for 5 min. Then, 0.4 mL of Al(NO_3_)_3_・9H_2_O (10%) was added, followed by the addition of 0.4 mL of 1 N NaOH after being kept for 6 min. The solution was then diluted with ethanol (30%) to a final volume of 10 mL and the absorbance was recorded at 510 nm. Total flavonoids were quantified and calculated using rutin as standard and expressed as g kg^−1^.

Total phenols content was determined according to a previous study [55] with minor modifications. Accurately 2 g of samples were homogenized with 10 mL of 80% methanol and subjected to ultrasonic extracted for 30 min, followed by centrifugation at 5000 g for 20 min. The supernatant (50 µL), distilled water (2 mL) and Folin-Ciocalteu phenol reagent (0.5 mL) were mixed well and incubated for 5 min in the dark. Then, 1 mL of sodium carbonate (5%) was added, and the mixture was diluted to 5 mL with distilled water. The absorbance of the solution was measured using a spectrophotometer at 750 nm after incubation for 1 h in the dark, and total phenols were quantified and calculated with gallic acid as a standard and expressed as g kg^-1^.

### Analysis of SOD enzyme activity and DPPH scavenging ability

The determination of SOD enzyme activity was conducted using a method previously described [56], with appropriate modifications. Accurately, 0.2 g of flesh tissue was homogenized in 1 mL of 50 mM potassium phosphate buffer (PBS, pH 7.8) at 4 °C, then centrifuged at 12,000 g for 20 min at 4 °C. The supernatant was collected for determination of SOD enzymes activities. A total of 3 mL mixture consisting of 2.7 mL of 14.5 mM methionine, 0.01 mL of 30 µM EDTA-Na_2_, 0.09 mL PBS (pH7.8), 0.1 mL of 2.25 mM nitroblue tetrazolium (NBT), 0.1 mL of 60 µM riboflavin, and the appropriate volume of plant extract. The reaction was initiated by light illumination. The inhibition of NBT photoreduction 50% as one enzyme activity unit (U g^-1^ ).

For DPPH scavenging ability analysis, 0.1 g of flesh tissue was ground in triplicate with 1 mL of 80% methanol on ice, then centrifuged at 12,000 rpm for 10 min at 4 °C. The supernatant was then used for the DPPH scavenging ability analysis with a DPPH scavenging ability assay kit (Nanjing Jiancheng Bioengineering, Nanjing, China).

### Widely targeted metabolome profiling

A widely targeted metabolome approach was conducted to compare global changes in metabolites between seasonal drought treatment (SD) and CK fruit. All samples analyses were undertaken by Wuhan Metware Biotechnology Co., Ltd. (Wuhan, China), as previously described [57], with some different parameters. The ultraperformance liquid chromatography-tandem mass spectrometry (UPLC-ESI-MS/MS) analysis included UPLC: ExionLC™ AD; MS/MS: AB SCIEX QTRAP 6500; Column: Agilent SB-C18: 1.8 μm, 2.1 mm × 100 mm. Metabolites were quantified based on orthogonal partial least squares-discriminant analysis (OPLS-DA) results, and a variable importance in projection (VIP) **>** 1 was considered to indicate differentially accumulated metabolites. Three biological replicates were carried out.

### Transcriptome sequencing and weighted gene coexpression correlation network analysis

Total RNA was extracted from seasonal drought (SD) treatment and CK fruit samples for PH0d, PH30d, and PH50d (with three biological repelicates) using the Trizol reagent (Invitrogen, CA, USA), following the manufacturer’s procedure. RNA sequencing was performed on an Illumina Novaseq^™^ 6000 platform (LC-Bio, Hangzhou, China) to produce raw reads according to the protocol, as described in our previous study [2]. Differentially expressed mRNAs and genes were selected based on log_2_ (fold change) > 1 or < -1 and with statistical significance (*q* value < 0.05) using the R package Ballgown [58]. DEGs were analyzed for the enrichment in GO and KEGG databases to identify the changes in biological functions and metabolism pathways [59]. Finally, the WGCNA package was employed in RStudio software to construct a gene coexpression network using a variant set of genes (13,646 genes, with the average FPKM in all samples ≥ 1) [60]. The main parameters of WGCNA program were: variance data expression > 0; no mission data expression < 0.1; soft threshold = 8 (scale-free R^2^ = 0.9); deepsplit = 2; min module size = 60; merge cut height = 0.25.

### Quantitative real-time PCR validation

The candidate DEGs were verified by qRT-PCR, which was performed according to our previous study [2]. The relative expression levels were calculated with the 2^−ΔΔCt^ method in three biological replications. The gene encoding *Actin* was used as the endogenous control. Specific primer were designed using Primer Express 3.0 (Applied Biosystems, Foster City, CA, USA). The primer sequences used in qRT-PCR analysis are listed in Table S1.

### Statistical analysis

Each experiment was conducted with three replicates using a completely randomized design and analyzed using the IBM SPSS program. All data were expressed as the mean ± standard deviation (SD). An asterisk (*) above the bars indicates significant differences at *p* < 0.05 between the drought treatment and the CK group, which were obtained based on an unpaired Student’s t-test. Histograms were generated with SigmaPlot 12.5, and the Pearson correlation heatmap of anthocyanin and related gene expression was visualized using the corrplot package in RStudio.

### Supplementary Information

**Supplementary Material 1.**

## Data Availability

The transcriptome data discussed in this publication have been deposited in NCBI’s Gene Expression Omnibus and are accessible through GEO Series accession number GSE253283 at https://www.ncbi.nlm.nih.gov/geo/query/acc.cgi?acc=GSE253283.

## Abbreviations

SD: Seasonal drought
WGCNA: Weighted gene coexpression correlation network analysis
PAL: Phenylalanine ammonia-lyase
RDI: Regulated deficit irrigation
CHS: Chalcone synthase
CHI: Chalcone isomerase
F3H: Flavanone 3-hydroxylase
F3’H: Flavonoid 3’-hydroxylase
DFR: Dihydroflavonol 4-reductase
ANS: Anthocyanidin synthase
UFGT: Uridine diphosphate-glucose:flavonoid 3-O-glucosyltransferase
GST: Glutathione S transferase
PH: Postharvest
TOA: Total organic acid
TSS: Total soluble solid
SOD: Superoxide dismutase
DPPH: 1,1-Diphenyl-2-picrylhydrazyl radical 2,2-Diphenyl-1-(2,4,6-trinitrophenyl)hydrazyl
DAMs: Differentially accumulated metabolites
DEGs: Differentially expressed genes
XTH9: Xyloglucan endotransglucosylase
LIP2: Octanoyltransferase
EXL1: GDSL esterase/lipase
LOX5: Probable linoleate 9s-lipoxygenase
FBP: F-box protein
Un: Unknown protein
qRT-PCR: Quantitative real-time PCR
C4H: Cinnamate 4-hydroxylase
FLS: Flavonol synthase
FBK: F-box proteins containing kelch repeats
CI: Color index
HPLC: High-performance liquid chromatography
FW: Fresh weight
PBS: Potassium phosphate buffer
NBT: Nitroblue tetrazolium
UPLC-ESI-MS/MS: Ultraperformance liquid chromatography-tandem mass spectrometry
OPLS-DA: Orthogonal partial least squares-discriminant analysis
