# Supplementary material for: Transcriptomics and metabolomics reveal the underlying mechanism of drought treatment on anthocyanin accumulation in postharvest blood orange fruit
Source: BMC Plant Biol. 2024 Mar 2;24:160. doi: 10.1186/s12870-024-04868-1 (PMC10908157; doi:10.1186/s12870-024-04868-1)
Supplement: Supplementary file 1 — Supplementary Material 1. [file 12870_2024_4868_MOESM1_ESM.docx]

Supplementary Material

**Transcriptomics and metabolomics reveals the underlying mechanism of seasonal drought on anthocyanin accumulation in postharvest blood orange fruits**

**Hongbin Liu^1,#^, Yan Jin^1,#^, Le Huang^1,#^, Chouyu Miao^1^, Jiayi Tang^1^, Huimin Zhang^1^, Haojie Yin^1^, Xiaopeng Lu^1^, Na Li^1^, Suming Dai^1^, Alessandra Gentile^1,2^, Ling Zhang^3^, Ling Sheng^1,*^**

^1^ National Center for Citrus Improvement Changsha, College of Horticulture, Hunan Agricultural University, 410128 Changsha, China

^2^ Department of Agriculture and Food Science, University of Catania, 95123 Catania, Italy

^3^ Agriculture and Rural Bureau of Mayang Miao Autonomous County, Huaihua, China

*******Correspondence:**

Corresponding Author

E-mail: [shengling0629@163.com](mailto:shengling0629@163.com)

^#^These authors contributed equally to this work.

Table S1. Primers used for quantitative real-time PCR analysis

| Gene Name | Gene ID | Primer 5’-3’ | |
| --- | --- | --- | --- |
| XTH9 | Cs_ont_2g020070 | ACCAGCGCCAAGTTGTATTT | TGGCTTGGTCCTTTGGGAAG |
| LIP2 | Cs_ont_2g033860 | TATGCCACAACCAAACGTGC | CCACCATCTTTGTTGCCCATT |
| EXL1 | Cs_ont_3g001920 | CGCCAGCATATCTCGATCCAAC | ATAAAGCTGCTGCCGAAAGTC |
| LOX5 | Cs_ont_3g016040 | TGCTGTGGTGATGATCGTGA | TCGCCATGAACAGCACTGAT |
| F-box | Cs_ont_9g017670 | TCCTCCGATTACCTCTGGCA | GGCGAAGATGAGGTTGTCGA |
| Un | Cs_ont_7g002490 | GAGCAGTATGGGCATCAGCA | TGCACCAGAAAGCACTACCA |
| PAL1 | Cs_ont_6g020600 | TTCAAGGGCGCTGAAATTGC | AATAGCCTGGCAAAGCGCTA |
| PAL2 | Cs_ont_6g020620 | AATGTGCCAGGGCAAGATCA | ACCATTTAGTCACATCGGCA |
| PAL3 | Cs_ont_7g006400 | GGTTTAGCTGCCACGGTACT | ATTTGTCCCGGATGGTGCTT |
| PAL4 | Cs_ont_8g005310 | GTCGTTGAATGCTGCTGAGG | ACGCAAGTCCCTCTTTAGGC |
| C4H | Cs_ont_1g006760 | ATTCCTCTTCTTGTGCCGCA | GCTTCTTCTCCCAACACCGA |
| CHS1 | Cs_ont_2g025700 | TCAAGCGCATGTGTGACAAG | CCACAATGTCTTGACGTGCG |
| CHS2 | Cs_ont_9g012610 | TCAAGCGCATGTGTGACAA | GCCATGTACGCACACACATT |
| CHI | Cs_ont_7g004690 | ACATCGTCGCCTTAGCTCTG | GACATCACTGCCACAGTCCA |
| CHIL | Cs_ont_7g003040 | GATGCTCTTGTCTCTGCTCCA | GTCCCTCACCGCACTTTCTA |
| F3H1 | Cs_ont_1g027890 | TTCAAGGGCGCTGAAATTGC | CTCCCATTGCTCAGATAATGACC |
| F3H2 | Cs_ont_2g002940 | AATGTGCCAGGGCAAGATCA | CCATGGTTCACCAACTGAAAGA |
| F3'H1 | Cs_ont_1g019190 | CCCGAGCGGCTAGAAAAGAT | CCACAAACCTGGGTCTCTCC |
| F3'H2 | Cs_ont_5g038970 | CACGTTCGCCAGGAAGAGAT | CAACGCACAAGTTCAACCGT |
| F3'H3 | Cs_ont_9g024140 | CACACTACCGTTTTGGCAGC | TACAAAGGGCAAGCCTCGAG |
| FLS | Cs_ont_1g020920 | TCTTTCTGCCCTCACCGTTC | CCCGATGTGGATGACGAGAG |
| DFR1 | Cs_ont_3g003380 | TCAATGTGCCCACAGAGTTCG | TGGCTCTGCAAGTATCCACAG |
| DFR2 | Cs_ont_3g016870 | GTGTCTTCTGGAGCTGCTGT | ACGTTGGACTGCAGAAGAGG |
| LAR | Cs_ont_7g019870 | CACCCTTCGGAGGTTCTTCC | CCAATGTCAGTGCCTGCAAC |
| ANS | Cs_ont_5g040910 | TTCAAGGGCGCTGAAATTGC | AAGCCAAGTGACAACACTGC |
| UFGT2 | Cs_ont_2g029410 | AATGTGCCAGGGCAAGATCA | TGGCTCTGGTGAAGGGAACA |
| UFGT3 | Cs_ont_5g016710 | CCGACTTGCCTGAAGGAGTT | GCTTGAAAGGGCCAACACTG |
| GST1 | Cs_ont_3g022020 | CGTCACTGCTGGCTTCAGAA | TGAGCAAATGGGCACGAGTA |
| GST2 | Cs_ont_6g006980 | CTTCTTCGACAGCCGTTTGG | GTTTGGCCCTTGGTTTGCAT |
| GST3 | Cs_ont_6g011580 | GGTTGCAAGGCTTCTCGTTG | GGTTGATCGTCGGGAAGGTT |

Table S2. Differentially accumulated metabolites in seasonal drought treated blood orange fruits compared with control

| Index | Class I | PH0d-CK1 | PH0d-CK2 | PH0d-CK3 | PH0d-T1 | PH0d-T2 | PH0d-T3 | VIP | FC | Type |
| --- | --- | --- | --- | --- | --- | --- | --- | --- | --- | --- |
| HJAP126 | Flavonoids | 69609 | 167384 | 591230 | 9 | 9 | 9 | 1.49 | 0.00 | down |
| Hmlp003185 | Flavonoids | 44176 | 9370 | 36540 | 9 | 9 | 9 | 1.49 | 0.00 | down |
| HJAP127 | Flavonoids | 2439050 | 1063965 | 1468068 | 462746 | 395819 | 430885 | 1.40 | 0.26 | down |
| Lmsp003881 | Flavonoids | 2188338 | 952234 | 4216530 | 531892 | 909565 | 681630 | 1.15 | 0.29 | down |
| Lmpp003268 | Flavonoids | 390078 | 174189 | 695030 | 142919 | 162918 | 99690 | 1.14 | 0.32 | down |
| Labn004865 | Flavonoids | 4062978 | 2989377 | 3051831 | 1315301 | 1368572 | 725903 | 1.39 | 0.34 | down |
| Lmfp003891 | Flavonoids | 302547 | 345992 | 372261 | 141269 | 134541 | 130370 | 1.49 | 0.40 | down |
| Lmsn002815 | Flavonoids | 7634296 | 9646409 | 8812615 | 3588748 | 3492481 | 3309342 | 1.48 | 0.40 | down |
| pmb0691 | Flavonoids | 15450 | 30390 | 30510 | 9709 | 8251 | 13932 | 1.26 | 0.42 | down |
| Jmgn004021 | Flavonoids | 3651348 | 3888105 | 3836915 | 1613757 | 1602647 | 1549126 | 1.50 | 0.42 | down |
| Lmlp005572 | Flavonoids | 38196705 | 66298605 | 32070184 | 17478959 | 19544194 | 22315245 | 1.30 | 0.43 | down |
| Lmsp004632 | Flavonoids | 1922945 | 1468097 | 1768060 | 774329 | 769730 | 742853 | 1.47 | 0.44 | down |
| Lmtn002796 | Flavonoids | 5732189 | 5396555 | 4547309 | 2447177 | 2303297 | 2199936 | 1.47 | 0.44 | down |
| Lmsp004670 | Flavonoids | 4570556 | 5178368 | 5182737 | 1302422 | 2873294 | 2445128 | 1.31 | 0.44 | down |
| pmb3023 | Flavonoids | 2526147 | 2694911 | 2534413 | 1119079 | 1199151 | 1147195 | 1.50 | 0.45 | down |
| Lmzn001875 | Flavonoids | 1888651 | 1811573 | 1885324 | 848382 | 828074 | 913105 | 1.50 | 0.46 | down |
| HJN087 | Flavonoids | 3279816 | 3890365 | 3053751 | 1557663 | 1311671 | 1952236 | 1.41 | 0.47 | down |
| Zmdn002049 | Flavonoids | 229137 | 250682 | 188205 | 93478 | 112222 | 113918 | 1.44 | 0.48 | down |
| Lmlp003531 | Flavonoids | 19127975 | 18043678 | 20265015 | 9208396 | 9994919 | 8365288 | 1.48 | 0.48 | down |
| Lmpp003930 | Flavonoids | 42030536 | 34183255 | 37022404 | 17197226 | 16641356 | 20718890 | 1.46 | 0.48 | down |
| pma0791 | Flavonoids | 66038 | 109935 | 88031 | 17001 | 50793 | 60401 | 1.05 | 0.49 | down |
| HJN055 | Flavonoids | 146645 | 170680 | 168830 | 78605 | 79339 | 85766 | 1.48 | 0.50 | down |
| MWSslk254 | Flavonoids | 4791617 | 4573754 | 4454244 | 2137753 | 1766844 | 3085272 | 1.36 | 0.51 | down |
| MWSHY0080 | Flavonoids | 4791617 | 4573754 | 4454244 | 2137753 | 1766844 | 3085272 | 1.36 | 0.51 | down |
| Lmsn003297 | Flavonoids | 205847 | 225932 | 196052 | 52374 | 151317 | 114729 | 1.15 | 0.51 | down |
| Hmpp003270 | Flavonoids | 24814041 | 26765516 | 24765536 | 11940225 | 13127753 | 13843781 | 1.48 | 0.51 | down |
| Lmwp003442 | Flavonoids | 43403821 | 41252391 | 35536408 | 21572738 | 22763964 | 19134630 | 1.46 | 0.53 | down |
| Lamp007163 | Flavonoids | 497523 | 653140 | 405353 | 288063 | 264358 | 271316 | 1.37 | 0.53 | down |
| Lmgp004474 | Flavonoids | 33856211 | 43384121 | 39727166 | 17047996 | 23090025 | 22659086 | 1.40 | 0.54 | down |
| pme0368 | Flavonoids | 45910332 | 35924105 | 36130160 | 25013172 | 18422556 | 20377416 | 1.40 | 0.54 | down |
| Zmhn001036 | Flavonoids | 354538 | 183437 | 397440 | 168660 | 213495 | 124673 | 1.07 | 0.54 | down |
| Lmgp004731 | Flavonoids | 37897889 | 33798642 | 35042831 | 19864814 | 20922041 | 17768477 | 1.47 | 0.55 | down |
| mws0072 | Flavonoids | 30454226 | 34741186 | 40676872 | 22091935 | 17532780 | 19234753 | 1.41 | 0.56 | down |
| mws1035 | Flavonoids | 4048184 | 4570528 | 3643358 | 2210489 | 2074840 | 2565224 | 1.43 | 0.56 | down |
| HJN090 | Flavonoids | 3332472 | 1706133 | 2519503 | 1708211 | 1770397 | 766114 | 1.01 | 0.56 | down |
| Zbjp003463 | Flavonoids | 2575220 | 3394585 | 2791278 | 1905697 | 1648162 | 1420060 | 1.39 | 0.57 | down |
| Zmjp003463 | Flavonoids | 2575220 | 3394585 | 2791278 | 1905697 | 1648162 | 1420060 | 1.39 | 0.57 | down |
| Zbsp005056 | Flavonoids | 170084 | 143225 | 190183 | 77656 | 110814 | 98659 | 1.36 | 0.57 | down |
| Zmbp006041 | Flavonoids | 37377 | 37318 | 43208 | 17707 | 22399 | 27213 | 1.36 | 0.57 | down |
| Zbpp001992 | Flavonoids | 1340339 | 1914057 | 2378933 | 982300 | 1469379 | 781342 | 1.12 | 0.57 | down |
| pmp000194 | Flavonoids | 318039 | 241931 | 248273 | 171896 | 136924 | 159190 | 1.39 | 0.58 | down |
| mws0047 | Flavonoids | 37654899 | 29712182 | 37120290 | 19934679 | 18916574 | 22058636 | 1.42 | 0.58 | down |
| Smhp004476 | Flavonoids | 2537976 | 3700319 | 3400755 | 1526445 | 1976850 | 2200227 | 1.29 | 0.59 | down |
| Lmmp002795 | Flavonoids | 193018 | 178259 | 157416 | 69248 | 102378 | 141294 | 1.19 | 0.59 | down |
| MWSHY0165 | Flavonoids | 3138573 | 2649126 | 1509730 | 1234120 | 1624631 | 1481328 | 1.07 | 0.59 | down |
| pmb2991 | Flavonoids | 33126 | 32546 | 23774 | 16273 | 16778 | 20313 | 1.34 | 0.60 | down |
| pmp000413 | Flavonoids | 49868035 | 50077877 | 50530145 | 29776753 | 28565885 | 31646309 | 1.49 | 0.60 | down |
| MWSHY0136 | Flavonoids | 22911342 | 21801252 | 25455440 | 14644238 | 13279238 | 14301531 | 1.47 | 0.60 | down |
| Lajp004590 | Flavonoids | 3003888 | 2731343 | 2630385 | 1475393 | 1799108 | 1774958 | 1.44 | 0.60 | down |
| pmb0681 | Flavonoids | 183666 | 176780 | 256473 | 154543 | 118143 | 100274 | 1.24 | 0.60 | down |
| HJN104 | Flavonoids | 72421 | 87693 | 62971 | 49026 | 42052 | 43870 | 1.38 | 0.60 | down |
| pmp001105 | Flavonoids | 901667 | 1038220 | 861034 | 772319 | 417913 | 527181 | 1.21 | 0.61 | down |
| Zbsp004438 | Flavonoids | 218974 | 145698 | 202843 | 138389 | 126350 | 86897 | 1.18 | 0.62 | down |
| MWSslk146 | Flavonoids | 25347479 | 29400701 | 31082992 | 17384764 | 17663809 | 18278528 | 1.45 | 0.62 | down |
| pmp000573 | Flavonoids | 347046 | 424317 | 383119 | 222195 | 249924 | 247111 | 1.44 | 0.62 | down |
| pmp000237 | Flavonoids | 3308225 | 3723216 | 2893113 | 2336395 | 1620919 | 2238534 | 1.30 | 0.62 | down |
| pmp000411 | Flavonoids | 2999248 | 3145658 | 3017403 | 2137476 | 1736877 | 1867873 | 1.45 | 0.63 | down |
| Zmdn002353 | Flavonoids | 183389 | 171129 | 161347 | 72421 | 94323 | 159606 | 1.10 | 0.63 | down |
| mws1066 | Flavonoids | 8222369 | 8586816 | 9878457 | 5731247 | 5834270 | 5386372 | 1.45 | 0.64 | down |
| Lamp006812 | Flavonoids | 494762 | 545863 | 477495 | 316414 | 357748 | 298806 | 1.44 | 0.64 | down |
| Smfp005803 | Flavonoids | 14905591 | 27407051 | 21684526 | 16198520 | 13810933 | 11065121 | 1.07 | 0.64 | down |
| pmp000238 | Flavonoids | 809239 | 1074528 | 739425 | 616954 | 488358 | 581203 | 1.28 | 0.64 | down |
| Lmyn005006 | Flavonoids | 11971796 | 12310420 | 12328520 | 8232936 | 7634593 | 7925041 | 1.49 | 0.65 | down |
| MWS20145 | Flavonoids | 2034448 | 2204580 | 2201755 | 1489353 | 1268134 | 1432280 | 1.45 | 0.65 | down |
| Xmyp005654 | Flavonoids | 28632633 | 25642392 | 27090110 | 15908955 | 18351602 | 18678349 | 1.45 | 0.65 | down |
| MWSHY0104 | Flavonoids | 28632633 | 25642392 | 27090110 | 15908955 | 18351602 | 18678349 | 1.45 | 0.65 | down |
| Zbsp004479 | Flavonoids | 4190106 | 4075117 | 3716409 | 3000634 | 2422378 | 2390056 | 1.40 | 0.65 | down |
| Hmcp001919 | Flavonoids | 511053 | 348667 | 508302 | 363343 | 243055 | 295232 | 1.16 | 0.66 | down |
| pmp000596 | Flavonoids | 138265 | 251788 | 213769 | 126367 | 144044 | 128746 | 1.09 | 0.66 | down |
| Zbsn003878 | Flavonoids | 939202 | 1230113 | 687780 | 598796 | 661669 | 634490 | 1.12 | 0.66 | down |
| Zajp002650 | Flavonoids | 7433821 | 4128686 | 5142051 | 4413483 | 3391194 | 3283211 | 1.06 | 0.66 | down |
| Lmlp002990 | Flavonoids | 19378184 | 15956342 | 19203584 | 12783455 | 11062111 | 12484153 | 1.40 | 0.67 | down |
| pmb0603 | Flavonoids | 87768 | 100040 | 66685 | 71037 | 58773 | 40447 | 1.07 | 0.67 | down |
| Lmnp202580 | Flavonoids | 3178219 | 3368365 | 2306345 | 2134961 | 1966234 | 1858614 | 1.26 | 0.67 | down |
| HJN041 | Flavonoids | 212578 | 140935 | 141109 | 108539 | 116167 | 111699 | 1.20 | 0.68 | down |
| mws1290 | Flavonoids | 3574674 | 3694702 | 3434134 | 2392176 | 2440803 | 2450946 | 1.49 | 0.68 | down |
| Hmgp003664 | Flavonoids | 215607 | 144013 | 127444 | 107554 | 103144 | 125901 | 1.07 | 0.69 | down |
| mws0046 | Flavonoids | 8378734 | 9310666 | 8640909 | 6450431 | 6118976 | 5694310 | 1.45 | 0.69 | down |
| Lamn006170 | Flavonoids | 1530017 | 1808055 | 1658382 | 1246917 | 1202233 | 1165753 | 1.43 | 0.72 | down |
| Hmbp002730 | Flavonoids | 4293377 | 4518481 | 4434633 | 3072288 | 3321318 | 3201955 | 1.48 | 0.72 | down |
| Lmmp004504 | Flavonoids | 4547205 | 4335439 | 3986996 | 3215489 | 3080073 | 3043298 | 1.45 | 0.73 | down |
| Zbzp003832 | Flavonoids | 48551099 | 44489299 | 52833901 | 37409352 | 34810457 | 33676512 | 1.41 | 0.73 | down |
| Zmjp005293 | Flavonoids | 487619 | 517956 | 567199 | 384957 | 340455 | 426131 | 1.34 | 0.73 | down |
| mws0914 | Flavonoids | 122402 | 120695 | 123127 | 80457 | 105027 | 83622 | 1.33 | 0.73 | down |
| pme0376 | Flavonoids | 122402 | 120695 | 123127 | 80457 | 105027 | 83622 | 1.33 | 0.73 | down |
| pme0088 | Flavonoids | 14986665 | 14055039 | 15988813 | 10653157 | 11348038 | 11102634 | 1.45 | 0.74 | down |
| MWSHY0145 | Flavonoids | 3709852 | 3572658 | 3656600 | 2564523 | 2648678 | 2885430 | 1.46 | 0.74 | down |
| pmf0472 | Flavonoids | 85480 | 90964 | 114085 | 64368 | 89735 | 61005 | 1.08 | 0.74 | down |
| pmb0626 | Flavonoids | 1090656 | 784281 | 892296 | 626844 | 747097 | 679436 | 1.20 | 0.74 | down |
| mws0791 | Flavonoids | 3960250 | 4291187 | 4502146 | 3244406 | 3217780 | 3017251 | 1.44 | 0.74 | down |
| Zbqn006065 | Flavonoids | 276578 | 282406 | 290561 | 204834 | 217774 | 208968 | 1.48 | 0.74 | down |
| Zbsp003798 | Flavonoids | 16654069 | 13464709 | 18310526 | 10228378 | 11308332 | 14496404 | 1.10 | 0.74 | down |
| Smgp004575 | Flavonoids | 12617657 | 14332833 | 13044284 | 10012335 | 10505059 | 9535306 | 1.42 | 0.75 | down |
| Hmpp002612 | Flavonoids | 1743936 | 1513514 | 1659879 | 1259640 | 1088231 | 1360968 | 1.32 | 0.75 | down |
| pmb0666 | Flavonoids | 501912 | 526129 | 485543 | 466422 | 318530 | 361768 | 1.18 | 0.76 | down |
| pmb0613 | Flavonoids | 1196194 | 1264192 | 1340087 | 975427 | 966184 | 937723 | 1.46 | 0.76 | down |
| Hmlp002975 | Flavonoids | 743788 | 576873 | 558493 | 475535 | 402913 | 548540 | 1.10 | 0.76 | down |
| pmp000383 | Flavonoids | 2089811 | 2237670 | 1916401 | 1487364 | 1720670 | 1537529 | 1.36 | 0.76 | down |
| Lmnp003080 | Flavonoids | 1429947 | 1407138 | 1137160 | 823743 | 1012362 | 1195857 | 1.09 | 0.76 | down |
| Lmdp004892 | Flavonoids | 8515451 | 7022858 | 8004798 | 5457666 | 6316571 | 6207431 | 1.32 | 0.76 | down |
| Lmjp003295 | Flavonoids | 22373156 | 21007852 | 19858827 | 17537796 | 12319585 | 18720852 | 1.08 | 0.77 | down |
| Lagp003663 | Flavonoids | 1055097 | 860463 | 1041229 | 670465 | 885777 | 733148 | 1.16 | 0.77 | down |
| Lahp004593 | Flavonoids | 313157 | 268874 | 301317 | 238546 | 171455 | 277020 | 1.00 | 0.78 | down |
| Zbdp005176 | Flavonoids | 3757276 | 3779494 | 3247753 | 2927088 | 2489258 | 3003608 | 1.28 | 0.78 | down |
| MWSHY0016 | Flavonoids | 1128352 | 1176587 | 1017049 | 891985 | 750265 | 956456 | 1.24 | 0.78 | down |
| Lmnp002961 | Flavonoids | 171148 | 155090 | 159441 | 130119 | 135978 | 124658 | 1.41 | 0.80 | down |
| pma0724 | Flavonoids | 809035 | 727698 | 951566 | 613694 | 772090 | 631066 | 1.05 | 0.81 | down |
| Lajp006633 | Flavonoids | 153798 | 130356 | 154314 | 121674 | 115355 | 118648 | 1.31 | 0.81 | down |
| Lmbp003668 | Flavonoids | 528370 | 401728 | 469763 | 406464 | 361435 | 378507 | 1.12 | 0.82 | down |
| Hmgp003641 | Flavonoids | 335212 | 331620 | 409642 | 300104 | 296379 | 293626 | 1.20 | 0.83 | down |
| Cmsp004086 | Flavonoids | 3373223 | 3717917 | 3822277 | 3137907 | 3069367 | 2822817 | 1.33 | 0.83 | down |
| Zbzp003271 | Flavonoids | 9254053 | 9996733 | 10643606 | 7372705 | 9515895 | 8346992 | 1.08 | 0.84 | down |
| Wagp010653 | Flavonoids | 469472 | 490202 | 455328 | 337782 | 414470 | 449475 | 1.04 | 0.85 | down |
| Lmmp003817 | Flavonoids | 7685426 | 7137959 | 6851905 | 6620302 | 5782081 | 6047866 | 1.26 | 0.85 | down |
| Lajp006279 | Flavonoids | 159998 | 136534 | 156043 | 135614 | 120359 | 130525 | 1.19 | 0.85 | down |
| Hmgp001902 | Flavonoids | 821234 | 890413 | 701236 | 640707 | 712450 | 709696 | 1.05 | 0.85 | down |
| pmp000239 | Flavonoids | 831970 | 856856 | 741930 | 627103 | 728675 | 728076 | 1.14 | 0.86 | down |
| mws0044 | Flavonoids | 500392 | 492563 | 553710 | 472174 | 398351 | 482843 | 1.04 | 0.88 | down |
| mws1519 | Flavonoids | 9218580 | 10303667 | 10054477 | 9076749 | 8875544 | 8051618 | 1.19 | 0.88 | down |
| Wagp008324 | Flavonoids | 1101443 | 1066283 | 1100964 | 959052 | 973502 | 948306 | 1.47 | 0.88 | down |
| Hajp008550 | Flavonoids | 421260 | 399047 | 464829 | 381365 | 392009 | 367917 | 1.16 | 0.89 | down |
| Wagp009375 | Flavonoids | 10529790 | 10564403 | 10620755 | 9223217 | 10276353 | 9451152 | 1.22 | 0.91 | down |
| MWSHC2050 | Flavonoids | 10529790 | 10564403 | 10620755 | 9223217 | 10276353 | 9451152 | 1.22 | 0.91 | down |
| Hmgp002036 | Flavonoids | 21646174 | 20728053 | 22128020 | 18649517 | 20341686 | 19976569 | 1.21 | 0.91 | down |
| Hamp007846 | Flavonoids | 10408335 | 10348101 | 10066152 | 9100198 | 9875011 | 9753433 | 1.19 | 0.93 | down |
| Zmsp007375 | Flavonoids | 15405274 | 14935446 | 14817494 | 14339606 | 14383465 | 14162750 | 1.34 | 0.95 | down |
| Wagp006380 | Flavonoids | 1289185 | 1254516 | 1196923 | 1304215 | 1297801 | 1478613 | 1.00 | 1.09 | up |
| pmp000117 | Flavonoids | 28185899 | 30122323 | 32343584 | 31307497 | 33880803 | 34421073 | 1.04 | 1.10 | up |
| mws0036 | Flavonoids | 14173824 | 16355865 | 14610364 | 17928267 | 17056281 | 15920548 | 1.11 | 1.13 | up |
| Zbsp003857 | Flavonoids | 11431888 | 11230200 | 10652208 | 11957023 | 13140292 | 12662319 | 1.31 | 1.13 | up |
| MWSHY0061 | Flavonoids | 6341305 | 7514567 | 7071339 | 7970799 | 7753485 | 8064688 | 1.18 | 1.14 | up |
| Wagp006939 | Flavonoids | 178572 | 214412 | 220721 | 236415 | 240984 | 223264 | 1.05 | 1.14 | up |
| HJAP023 | Flavonoids | 1746872 | 1893537 | 1864808 | 2066541 | 2149937 | 2108968 | 1.40 | 1.15 | up |
| pmp000013 | Flavonoids | 203268 | 200073 | 200572 | 208892 | 233076 | 252932 | 1.17 | 1.15 | up |
| Zmcn004206 | Flavonoids | 355547 | 378854 | 360416 | 425213 | 443092 | 392026 | 1.30 | 1.15 | up |
| Lmlp005236 | Flavonoids | 1821119 | 2030419 | 2375082 | 2390139 | 2401834 | 2385786 | 1.04 | 1.15 | up |
| Wagp010352 | Flavonoids | 624789 | 701591 | 684480 | 774389 | 840947 | 732909 | 1.24 | 1.17 | up |
| Lmsp010098 | Flavonoids | 45744 | 36666 | 33984 | 46961 | 45073 | 44079 | 1.01 | 1.17 | up |
| Wcfp003437 | Flavonoids | 279281 | 259921 | 274285 | 318079 | 306173 | 327333 | 1.41 | 1.17 | up |
| Wagp005905 | Flavonoids | 182075 | 213003 | 164890 | 212405 | 239922 | 205744 | 1.02 | 1.18 | up |
| Zmxp004480 | Flavonoids | 256080 | 342316 | 302141 | 357785 | 342989 | 372893 | 1.08 | 1.19 | up |
| Smhp007666 | Flavonoids | 197776 | 208679 | 173390 | 264516 | 209050 | 218484 | 1.04 | 1.19 | up |
| Hmcp001598 | Flavonoids | 111917 | 108461 | 123044 | 134750 | 144809 | 133112 | 1.34 | 1.20 | up |
| Wagp009942 | Flavonoids | 598071 | 660461 | 687309 | 809939 | 778525 | 761911 | 1.36 | 1.21 | up |
| pmp000008 | Flavonoids | 613035 | 648994 | 615954 | 768886 | 713697 | 787232 | 1.41 | 1.21 | up |
| Wagp006908 | Flavonoids | 83543 | 101041 | 106904 | 119343 | 124645 | 112187 | 1.18 | 1.22 | up |
| MWSHY0054 | Flavonoids | 104164 | 95137 | 90934 | 127047 | 115923 | 111762 | 1.32 | 1.22 | up |
| Zmsp004363 | Flavonoids | 292243 | 268279 | 313685 | 332263 | 361817 | 379871 | 1.30 | 1.23 | up |
| Hmgp003086 | Flavonoids | 242726 | 341460 | 292181 | 342010 | 341246 | 393545 | 1.05 | 1.23 | up |
| Wagp007022 | Flavonoids | 1278550 | 1105331 | 910350 | 1201616 | 1369200 | 1496644 | 1.02 | 1.23 | up |
| Wagp007859 | Flavonoids | 34300 | 40657 | 37002 | 49944 | 42678 | 45896 | 1.27 | 1.24 | up |
| pmp001309 | Flavonoids | 16388964 | 13105300 | 12101912 | 16119023 | 17477535 | 18133328 | 1.14 | 1.24 | up |
| Lmfn004093 | Flavonoids | 407322 | 538367 | 510107 | 576646 | 649999 | 586777 | 1.16 | 1.25 | up |
| HJAP026 | Flavonoids | 2242129 | 2383271 | 2591018 | 2928710 | 3128532 | 2998765 | 1.39 | 1.25 | up |
| mws1002 | Flavonoids | 21349 | 22517 | 25671 | 25271 | 29954 | 32180 | 1.17 | 1.26 | up |
| Wagp010987 | Flavonoids | 501140 | 528978 | 546535 | 652876 | 649445 | 681313 | 1.45 | 1.26 | up |
| Zbgp008956 | Flavonoids | 880280 | 893399 | 817544 | 1143282 | 982537 | 1143836 | 1.34 | 1.26 | up |
| Cmyp002064 | Flavonoids | 9420624 | 9275212 | 9376409 | 11131568 | 12177607 | 12165936 | 1.45 | 1.26 | up |
| pmp000110 | Flavonoids | 218801 | 224163 | 224753 | 277247 | 262554 | 307866 | 1.39 | 1.27 | up |
| HJN051 | Flavonoids | 10665509 | 14011773 | 10488975 | 14237362 | 15908980 | 14565880 | 1.17 | 1.27 | up |
| pmp000006 | Flavonoids | 126284 | 170394 | 133912 | 170855 | 186548 | 200956 | 1.19 | 1.30 | up |
| Hmxp007703 | Flavonoids | 431844 | 432004 | 423831 | 599689 | 539181 | 532492 | 1.44 | 1.30 | up |
| Lamn005190 | Flavonoids | 903911 | 885047 | 1008479 | 1233231 | 1235948 | 1171596 | 1.42 | 1.30 | up |
| Wagp007236 | Flavonoids | 202546 | 184851 | 218285 | 256102 | 267691 | 264531 | 1.40 | 1.30 | up |
| Wagp006554 | Flavonoids | 187181 | 207253 | 170921 | 256306 | 244943 | 237485 | 1.37 | 1.31 | up |
| Hmgp002121 | Flavonoids | 29100028 | 31837508 | 22767760 | 34825367 | 33884537 | 40852753 | 1.15 | 1.31 | up |
| Wagp009231 | Flavonoids | 580471 | 645728 | 574903 | 778693 | 783122 | 812274 | 1.44 | 1.32 | up |
| Wagp006264 | Flavonoids | 438462 | 414832 | 403574 | 594854 | 522069 | 550754 | 1.43 | 1.33 | up |
| Zbsp003971 | Flavonoids | 793368 | 898659 | 653572 | 1107822 | 1114526 | 891980 | 1.16 | 1.33 | up |
| Wagp008700 | Flavonoids | 264594 | 204342 | 194903 | 298619 | 281517 | 301905 | 1.25 | 1.33 | up |
| MWSHY0089 | Flavonoids | 50828 | 71182 | 78955 | 85984 | 93022 | 88532 | 1.12 | 1.33 | up |
| pmb3002 | Flavonoids | 19419916 | 21308052 | 19506051 | 25009702 | 26028374 | 29276973 | 1.40 | 1.33 | up |
| Wagp008723 | Flavonoids | 70150 | 83633 | 91414 | 108436 | 106634 | 112366 | 1.32 | 1.34 | up |
| Wagp007778 | Flavonoids | 150908 | 188577 | 148999 | 227045 | 216049 | 213730 | 1.33 | 1.34 | up |
| HJAP011 | Flavonoids | 17853895 | 20428756 | 17429683 | 24942996 | 24180819 | 25826599 | 1.41 | 1.35 | up |
| Lmzn001961 | Flavonoids | 105626 | 105655 | 103420 | 122752 | 151479 | 149288 | 1.36 | 1.35 | up |
| mws2118 | Flavonoids | 32597 | 37624 | 44656 | 49970 | 44670 | 60065 | 1.15 | 1.35 | up |
| Wagp009533 | Flavonoids | 273990 | 295009 | 284456 | 392291 | 384329 | 376613 | 1.48 | 1.35 | up |
| Hajp007218 | Flavonoids | 50014 | 69163 | 71798 | 84235 | 100048 | 75712 | 1.12 | 1.36 | up |
| MWSHC20110 | Flavonoids | 50014 | 69163 | 71798 | 84235 | 100048 | 75712 | 1.12 | 1.36 | up |
| pmp001080 | Flavonoids | 6687572 | 5498366 | 6319161 | 8711137 | 8092312 | 8422048 | 1.40 | 1.36 | up |
| Lahn005548 | Flavonoids | 19759979 | 18559638 | 17769516 | 25302067 | 26571856 | 24855818 | 1.46 | 1.37 | up |
| Wagp005934 | Flavonoids | 415878 | 383502 | 412269 | 530474 | 540117 | 598240 | 1.44 | 1.38 | up |
| Wagp006347 | Flavonoids | 228782 | 203184 | 192603 | 307308 | 252492 | 303259 | 1.34 | 1.38 | up |
| Lmsp010393 | Flavonoids | 288507 | 284437 | 298849 | 414641 | 371304 | 421740 | 1.45 | 1.39 | up |
| pma0795 | Flavonoids | 239261 | 271347 | 208821 | 339346 | 360624 | 299437 | 1.31 | 1.39 | up |
| Lmjn005592 | Flavonoids | 46069 | 53724 | 54513 | 71056 | 73450 | 70545 | 1.42 | 1.39 | up |
| Cmxn004016 | Flavonoids | 321452 | 295442 | 293999 | 441882 | 417886 | 410856 | 1.47 | 1.39 | up |
| pmp001006 | Flavonoids | 32406 | 41977 | 38861 | 56186 | 43681 | 58173 | 1.22 | 1.40 | up |
| mws1329 | Flavonoids | 4385826 | 3578158 | 4222002 | 5731878 | 6281159 | 5093158 | 1.33 | 1.40 | up |
| Zmhp004065 | Flavonoids | 148819 | 136380 | 130138 | 195238 | 189407 | 198388 | 1.46 | 1.40 | up |
| Wagp009413 | Flavonoids | 83490 | 65224 | 75462 | 93315 | 100350 | 121103 | 1.27 | 1.40 | up |
| Lajp005753 | Flavonoids | 530949 | 509816 | 507380 | 644368 | 774314 | 758228 | 1.41 | 1.41 | up |
| Lmhp206353 | Flavonoids | 530949 | 509816 | 507380 | 644368 | 774314 | 758228 | 1.41 | 1.41 | up |
| Wagp007545 | Flavonoids | 76972 | 83307 | 97000 | 110099 | 137553 | 114345 | 1.31 | 1.41 | up |
| Hajp007261 | Flavonoids | 76972 | 83307 | 97000 | 110099 | 137553 | 114345 | 1.31 | 1.41 | up |
| Hmhp005846 | Flavonoids | 143927 | 138629 | 117784 | 185487 | 189404 | 188925 | 1.41 | 1.41 | up |
| pmb0628 | Flavonoids | 235893 | 141656 | 207005 | 243440 | 265595 | 316547 | 1.09 | 1.41 | up |
| Zmjp009119 | Flavonoids | 74818 | 59241 | 85484 | 120505 | 96742 | 94298 | 1.20 | 1.42 | up |
| Wagp005792 | Flavonoids | 954642 | 882057 | 951103 | 1363240 | 1275848 | 1317931 | 1.48 | 1.42 | up |
| Lmnp002845 | Flavonoids | 20276439 | 20167498 | 19436153 | 29097267 | 27460533 | 28658776 | 1.49 | 1.42 | up |
| Lmsp003939 | Flavonoids | 943740 | 877741 | 988158 | 1272431 | 1603154 | 1123306 | 1.26 | 1.42 | up |
| Lmcn005735 | Flavonoids | 1758405 | 1945959 | 1760690 | 2520299 | 2536543 | 2767452 | 1.46 | 1.43 | up |
| Wagp010678 | Flavonoids | 559295 | 621543 | 602383 | 797780 | 922649 | 835927 | 1.44 | 1.43 | up |
| pmp000179 | Flavonoids | 541012 | 552628 | 476932 | 909610 | 611002 | 738680 | 1.23 | 1.44 | up |
| pmp000120 | Flavonoids | 11918605 | 11635331 | 11962292 | 16135701 | 17437464 | 17639937 | 1.48 | 1.44 | up |
| Zbsp002894 | Flavonoids | 8903399 | 7243787 | 6748580 | 10743275 | 8410811 | 13939964 | 1.09 | 1.45 | up |
| Lajp005820 | Flavonoids | 123455 | 123953 | 109726 | 172745 | 160734 | 182857 | 1.44 | 1.45 | up |
| Lmgp004959 | Flavonoids | 274399 | 312083 | 233882 | 319439 | 433712 | 461669 | 1.21 | 1.48 | up |
| Wagp005210 | Flavonoids | 976787 | 804958 | 1141565 | 1686958 | 1264687 | 1393138 | 1.25 | 1.49 | up |
| Lmjp002596 | Flavonoids | 24867 | 56776 | 38668 | 57581 | 60163 | 62012 | 1.04 | 1.49 | up |
| Zmhp002409 | Flavonoids | 2008933 | 1592728 | 2171194 | 3260313 | 2651232 | 2827513 | 1.33 | 1.51 | up |
| Layp005773 | Flavonoids | 1188827 | 1002212 | 1097534 | 1621663 | 1580228 | 1776823 | 1.44 | 1.51 | up |
| Zbsp004301 | Flavonoids | 3806114 | 2268670 | 5512970 | 6568950 | 5109470 | 6006456 | 1.00 | 1.53 | up |
| Lmcp007155 | Flavonoids | 3486392 | 3492305 | 3510203 | 6483889 | 6057419 | 3505253 | 1.06 | 1.53 | up |
| pmp001076 | Flavonoids | 3486392 | 3492305 | 3510203 | 6483889 | 6057419 | 3505253 | 1.06 | 1.53 | up |
| Zmhn002574 | Flavonoids | 1871618 | 1638403 | 1901088 | 2927261 | 2587598 | 2840170 | 1.45 | 1.54 | up |
| Wagp005964 | Flavonoids | 201746 | 197333 | 153960 | 309150 | 252578 | 293720 | 1.35 | 1.55 | up |
| Wagp004758 | Flavonoids | 197821 | 146249 | 256048 | 263580 | 342825 | 331220 | 1.18 | 1.56 | up |
| Wagp005813 | Flavonoids | 1231156 | 1112500 | 1022129 | 1768943 | 1727355 | 1796167 | 1.46 | 1.57 | up |
| pmn001696 | Flavonoids | 52711 | 58342 | 61332 | 91726 | 86334 | 93016 | 1.47 | 1.57 | up |
| Lmjp003722 | Flavonoids | 43762 | 50834 | 52833 | 52210 | 75847 | 106517 | 1.06 | 1.59 | up |
| Zmhp004269 | Flavonoids | 73106 | 69404 | 75105 | 125487 | 109807 | 114270 | 1.47 | 1.61 | up |
| pmp000593 | Flavonoids | 825486 | 823186 | 1383442 | 1762188 | 1147005 | 1992132 | 1.07 | 1.62 | up |
| Lmsp004749 | Flavonoids | 1139974 | 948739 | 1027385 | 1380573 | 1579190 | 2102154 | 1.30 | 1.62 | up |
| Lmmp004749 | Flavonoids | 10035 | 17365 | 9959 | 17166 | 24069 | 20005 | 1.17 | 1.64 | up |
| Hmcp002399 | Flavonoids | 251587 | 284224 | 148702 | 364487 | 346470 | 411165 | 1.19 | 1.64 | up |
| pmn001527 | Flavonoids | 81245 | 88190 | 98622 | 145372 | 159545 | 137985 | 1.44 | 1.65 | up |
| Zmhp003514 | Flavonoids | 64848 | 53910 | 58313 | 83231 | 111733 | 98429 | 1.39 | 1.66 | up |
| pmp000115 | Flavonoids | 72791 | 75889 | 80631 | 139327 | 111546 | 131751 | 1.44 | 1.67 | up |
| Wagp005796 | Flavonoids | 169923 | 193482 | 134375 | 291651 | 326871 | 212440 | 1.25 | 1.67 | up |
| Wagp006119 | Flavonoids | 486132 | 435199 | 611564 | 794195 | 965305 | 800551 | 1.36 | 1.67 | up |
| pmp000128 | Flavonoids | 534962 | 638218 | 557760 | 1336979 | 761753 | 792659 | 1.18 | 1.67 | up |
| Wagp005137 | Flavonoids | 76298 | 59921 | 54233 | 120893 | 103111 | 94841 | 1.36 | 1.67 | up |
| pme3514 | Flavonoids | 460954 | 922260 | 429376 | 1237390 | 747496 | 1155721 | 1.07 | 1.73 | up |
| HJAP005 | Flavonoids | 201807 | 214095 | 163654 | 255322 | 426363 | 333538 | 1.27 | 1.75 | up |
| pmb3000 | Flavonoids | 1180361 | 1466043 | 1181391 | 2398405 | 2217508 | 2101407 | 1.44 | 1.75 | up |
| Wagp005333 | Flavonoids | 433558 | 589002 | 602251 | 1076424 | 994460 | 839505 | 1.37 | 1.79 | up |
| Hmcp001628 | Flavonoids | 196275 | 201659 | 251042 | 517488 | 315537 | 341257 | 1.29 | 1.81 | up |
| Hmcp001579 | Flavonoids | 196275 | 201659 | 251042 | 517488 | 315537 | 341257 | 1.29 | 1.81 | up |
| pmp000127 | Flavonoids | 12850633 | 5576744 | 6164381 | 13498622 | 16234559 | 14784659 | 1.17 | 1.81 | up |
| Zmhp002401 | Flavonoids | 197522 | 169807 | 175157 | 315381 | 353212 | 317797 | 1.47 | 1.82 | up |
| Wagp006295 | Flavonoids | 199075 | 231790 | 159969 | 290971 | 379520 | 413470 | 1.34 | 1.83 | up |
| Lajp005489 | Flavonoids | 111715 | 373257 | 403579 | 544044 | 518005 | 612428 | 1.02 | 1.88 | up |
| Wagp007355 | Flavonoids | 17758 | 20079 | 18047 | 24785 | 26618 | 55639 | 1.12 | 1.92 | up |
| Wagp003712 | Flavonoids | 87946 | 101964 | 135354 | 254916 | 239134 | 135070 | 1.20 | 1.93 | up |
| Lmdp008003 | Flavonoids | 63932 | 55589 | 60446 | 104191 | 127927 | 116927 | 1.47 | 1.94 | up |
| Hmmp007513 | Flavonoids | 63932 | 55589 | 60446 | 104191 | 127927 | 116927 | 1.47 | 1.94 | up |
| Zmjp004852 | Flavonoids | 2033480 | 2120645 | 2036494 | 4424309 | 3308525 | 4541509 | 1.44 | 1.98 | up |
| Wagp004981 | Flavonoids | 2033480 | 2120645 | 2036494 | 4424309 | 3308525 | 4541509 | 1.44 | 1.98 | up |
| pmb3053 | Flavonoids | 3512 | 2670 | 4151 | 6534 | 6760 | 7359 | 1.40 | 2.00 | up |
| Wahp004750 | Flavonoids | 51743 | 85496 | 95227 | 114314 | 223515 | 132248 | 1.17 | 2.02 | up |
| pmp000588 | Flavonoids | 30901 | 20160 | 13198 | 43826 | 42028 | 44946 | 1.26 | 2.04 | up |
| Wagp006002 | Flavonoids | 196335 | 144018 | 198475 | 483798 | 238330 | 376340 | 1.24 | 2.04 | up |
| Hahp004959 | Flavonoids | 1353651 | 1069931 | 1458933 | 2161832 | 2220281 | 3618768 | 1.32 | 2.06 | up |
| pmp000004 | Flavonoids | 34334 | 39709 | 32427 | 76186 | 62983 | 80305 | 1.45 | 2.06 | up |
| HJAP006 | Flavonoids | 2099316 | 2130054 | 1653652 | 4007924 | 3795489 | 4399966 | 1.46 | 2.07 | up |
| Hmcp005575 | Flavonoids | 43345 | 112419 | 41126 | 182074 | 129410 | 102067 | 1.12 | 2.10 | up |
| Hmxp008844 | Flavonoids | 202440 | 243043 | 214206 | 448558 | 450078 | 498812 | 1.48 | 2.12 | up |
| Lmjp004941 | Flavonoids | 27901 | 36756 | 51436 | 77654 | 92413 | 76297 | 1.35 | 2.12 | up |
| MWSHY0069 | Flavonoids | 27901 | 36756 | 51436 | 77654 | 92413 | 76297 | 1.35 | 2.12 | up |
| Wagp006055 | Flavonoids | 156303 | 137336 | 139029 | 299348 | 329891 | 292630 | 1.48 | 2.13 | up |
| Zbnp004535 | Flavonoids | 218755 | 202157 | 156725 | 468086 | 405415 | 374680 | 1.43 | 2.16 | up |
| Hmcp002187 | Flavonoids | 1910014 | 1863515 | 1612700 | 3801335 | 3807969 | 4152421 | 1.48 | 2.18 | up |
| Wagp005336 | Flavonoids | 172338 | 258889 | 254469 | 592455 | 337877 | 576071 | 1.30 | 2.20 | up |
| pmb2999 | Flavonoids | 26966 | 43759 | 34191 | 99048 | 84390 | 48216 | 1.24 | 2.21 | up |
| pmp000241 | Flavonoids | 2504357 | 2793299 | 2667623 | 5927432 | 4859675 | 6810405 | 1.45 | 2.21 | up |
| Wagp006311 | Flavonoids | 32682 | 20985 | 75995 | 95551 | 96922 | 94238 | 1.17 | 2.21 | up |
| Lmmp002963 | Flavonoids | 3343012 | 4480930 | 7041020 | 16353561 | 10022229 | 6659648 | 1.13 | 2.22 | up |
| Wagp005347 | Flavonoids | 17465 | 11670 | 55165 | 77500 | 49900 | 64046 | 1.10 | 2.27 | up |
| HJAP020 | Flavonoids | 9437 | 10210 | 16484 | 18177 | 19445 | 45010 | 1.12 | 2.29 | up |
| Lmzn002255 | Flavonoids | 80808 | 73331 | 75548 | 132817 | 174297 | 219192 | 1.41 | 2.29 | up |
| Zmjp004875 | Flavonoids | 137242 | 125689 | 142852 | 300761 | 340360 | 291146 | 1.48 | 2.30 | up |
| pmn001551 | Flavonoids | 2476 | 3239 | 2222 | 6124 | 6840 | 5449 | 1.43 | 2.32 | up |
| Wagp004984 | Flavonoids | 119039 | 88483 | 145934 | 275738 | 273328 | 293373 | 1.42 | 2.38 | up |
| Hmcp001947 | Flavonoids | 56170 | 23517 | 65590 | 126492 | 92155 | 131166 | 1.23 | 2.41 | up |
| Lmjp005224 | Flavonoids | 15894 | 17650 | 31408 | 54967 | 59562 | 42508 | 1.34 | 2.42 | up |
| pmp000240 | Flavonoids | 235423 | 221575 | 240154 | 448491 | 597561 | 644434 | 1.45 | 2.42 | up |
| Wagp005996 | Flavonoids | 22443 | 30423 | 23207 | 47778 | 85030 | 51680 | 1.35 | 2.43 | up |
| Wagp004055 | Flavonoids | 164247 | 155346 | 84280 | 206337 | 325871 | 453798 | 1.22 | 2.44 | up |
| Zbnp003712 | Flavonoids | 164247 | 155346 | 84280 | 206337 | 325871 | 453798 | 1.22 | 2.44 | up |
| Hmhp005160 | Flavonoids | 20280 | 36030 | 29727 | 76207 | 66091 | 75139 | 1.41 | 2.53 | up |
| pmb0608 | Flavonoids | 54602 | 40952 | 49603 | 137097 | 113303 | 120338 | 1.47 | 2.55 | up |
| Wagp005556 | Flavonoids | 73656 | 105573 | 142590 | 213499 | 303980 | 309786 | 1.36 | 2.57 | up |
| Zbnp004589 | Flavonoids | 623030 | 1273055 | 1195630 | 3197156 | 3623875 | 1559811 | 1.22 | 2.71 | up |
| pmp000589 | Flavonoids | 30462 | 22086 | 38839 | 63240 | 95035 | 108163 | 1.38 | 2.92 | up |
| Wagp004602 | Flavonoids | 301073 | 227592 | 197687 | 652254 | 656395 | 834975 | 1.45 | 2.95 | up |
| Lmxn007955 | Flavonoids | 22464 | 23040 | 20278 | 60548 | 72767 | 68949 | 1.49 | 3.07 | up |
| Wagp004789 | Flavonoids | 179007 | 118115 | 189634 | 613996 | 577225 | 479136 | 1.45 | 3.43 | up |
| pmb0542 | Flavonoids | 38345 | 77848 | 71726 | 1066318 | 1030474 | 1198837 | 1.48 | 17.54 | up |
| MWSHY0135 | Flavonoids | 9 | 9 | 9 | 16750444 | 15698183 | 19169367 | 1.50 | 1911778 | up |
| Lajp003510 | Phenolic acids | 186276 | 157932 | 218437 | 54085 | 81096 | 59120 | 1.44 | 0.35 | down |
| Lakn003294 | Phenolic acids | 1156305 | 974251 | 911557 | 400958 | 333637 | 322638 | 1.48 | 0.35 | down |
| Lmdn003756 | Phenolic acids | 1466289 | 1619426 | 1604020 | 579716 | 528176 | 559826 | 1.50 | 0.36 | down |
| Zmhn001793 | Phenolic acids | 5858215 | 6758429 | 5554863 | 2532565 | 2302164 | 2421280 | 1.49 | 0.40 | down |
| Hmgn002038 | Phenolic acids | 331668 | 237412 | 265571 | 108428 | 126869 | 114066 | 1.46 | 0.42 | down |
| mws0906 | Phenolic acids | 6048885 | 5960665 | 6169006 | 2897543 | 2295577 | 2517503 | 1.48 | 0.42 | down |
| pmn001420 | Phenolic acids | 5220617 | 8105641 | 6428305 | 4367640 | 3086658 | 1448518 | 1.17 | 0.45 | down |
| Lmgn002253 | Phenolic acids | 13147 | 13512 | 15509 | 7867 | 1893 | 10345 | 1.01 | 0.48 | down |
| Lmqp002761 | Phenolic acids | 203098 | 183779 | 215326 | 127119 | 108052 | 60635 | 1.28 | 0.49 | down |
| Waln003742 | Phenolic acids | 31303 | 30185 | 40586 | 14548 | 21149 | 17566 | 1.38 | 0.52 | down |
| Hmcn001296 | Phenolic acids | 241321 | 218517 | 206127 | 122932 | 113747 | 112992 | 1.48 | 0.53 | down |
| Lmhn002573 | Phenolic acids | 40244790 | 37384727 | 41712208 | 21149588 | 22973934 | 20403093 | 1.48 | 0.54 | down |
| Lman002731 | Phenolic acids | 1055143 | 937056 | 925291 | 725713 | 514807 | 417138 | 1.31 | 0.57 | down |
| Wdtp003528 | Phenolic acids | 1396183 | 1346216 | 1396316 | 951505 | 870995 | 574955 | 1.32 | 0.58 | down |
| mws0011 | Phenolic acids | 2057853 | 2617738 | 3127745 | 1371064 | 1689136 | 1482987 | 1.34 | 0.58 | down |
| Lmsn002288 | Phenolic acids | 8056052 | 7050060 | 6316648 | 3429065 | 4440976 | 4807185 | 1.36 | 0.59 | down |
| pme2828 | Phenolic acids | 16601250 | 17338001 | 16516976 | 10067444 | 10198578 | 9687526 | 1.50 | 0.59 | down |
| Smhn003633 | Phenolic acids | 1556786 | 1759460 | 1929630 | 1003162 | 1121435 | 996897 | 1.44 | 0.60 | down |
| Lmhn002321 | Phenolic acids | 165966 | 169297 | 135228 | 122937 | 74441 | 87948 | 1.27 | 0.61 | down |
| mws0179 | Phenolic acids | 283300 | 259545 | 221275 | 127158 | 214502 | 124903 | 1.21 | 0.61 | down |
| Lmtn003598 | Phenolic acids | 361873 | 338368 | 390377 | 240605 | 206616 | 234473 | 1.45 | 0.63 | down |
| pmb2871 | Phenolic acids | 281553 | 275103 | 479089 | 193167 | 186095 | 271338 | 1.07 | 0.63 | down |
| pmn001367 | Phenolic acids | 281553 | 275103 | 479089 | 193167 | 186095 | 271338 | 1.07 | 0.63 | down |
| Wafn002491 | Phenolic acids | 10235332 | 11909281 | 10806233 | 6206182 | 7443781 | 7103327 | 1.44 | 0.63 | down |
| mws0028 | Phenolic acids | 33409 | 50181 | 63405 | 27226 | 25618 | 40064 | 1.03 | 0.63 | down |
| pme1816 | Phenolic acids | 281342 | 340795 | 290695 | 157598 | 238112 | 196277 | 1.29 | 0.65 | down |
| mws2212 | Phenolic acids | 2580250 | 2716132 | 2734203 | 1724186 | 1808406 | 1735472 | 1.49 | 0.66 | down |
| Lmhn003074 | Phenolic acids | 56256 | 63986 | 70690 | 43937 | 43952 | 40378 | 1.41 | 0.67 | down |
| pmn001553 | Phenolic acids | 628035 | 767120 | 886331 | 554568 | 496786 | 500498 | 1.31 | 0.68 | down |
| Lmsn004970 | Phenolic acids | 159650 | 156456 | 146557 | 114113 | 115244 | 86636 | 1.34 | 0.68 | down |
| Zmhn004909 | Phenolic acids | 63646 | 48669 | 55984 | 36803 | 37139 | 42120 | 1.35 | 0.69 | down |
| mws4085 | Phenolic acids | 227175 | 184357 | 171909 | 163317 | 130065 | 115540 | 1.21 | 0.70 | down |
| HJAP051 | Phenolic acids | 146048 | 145125 | 106260 | 82692 | 96972 | 101001 | 1.22 | 0.71 | down |
| Qazn005429 | Phenolic acids | 89291 | 104943 | 81458 | 81729 | 50337 | 64865 | 1.11 | 0.71 | down |
| Zblp002839 | Phenolic acids | 1223556 | 1102032 | 1108790 | 805846 | 864562 | 811202 | 1.46 | 0.72 | down |
| pmb3142 | Phenolic acids | 226142 | 299139 | 291102 | 210982 | 181960 | 208998 | 1.24 | 0.74 | down |
| Wafn001957 | Phenolic acids | 1285212 | 1403692 | 1279391 | 1039695 | 898625 | 1076958 | 1.36 | 0.76 | down |
| pme0422 | Phenolic acids | 6032019 | 5242637 | 5625729 | 4327245 | 3956453 | 4591674 | 1.38 | 0.76 | down |
| pmn001421 | Phenolic acids | 168302 | 155866 | 166085 | 104395 | 148417 | 128789 | 1.17 | 0.78 | down |
| Zmtn001661 | Phenolic acids | 469857 | 458746 | 460305 | 360091 | 364974 | 365963 | 1.50 | 0.79 | down |
| Wasn004683 | Phenolic acids | 1067365 | 1312232 | 1195700 | 956220 | 904335 | 979385 | 1.31 | 0.79 | down |
| NK10264324 | Phenolic acids | 3377749 | 3132611 | 3673065 | 2761663 | 2587395 | 2760394 | 1.37 | 0.80 | down |
| Lmdp003146 | Phenolic acids | 884678 | 963369 | 795264 | 756680 | 720988 | 648010 | 1.25 | 0.80 | down |
| Wasn003200 | Phenolic acids | 10153956 | 11722223 | 8930710 | 7953044 | 8273602 | 8648054 | 1.18 | 0.81 | down |
| Hmtn001120 | Phenolic acids | 283755 | 294310 | 269732 | 208667 | 212802 | 263804 | 1.21 | 0.81 | down |
| Ymjm000148 | Phenolic acids | 481895 | 522680 | 506038 | 393286 | 445705 | 402191 | 1.37 | 0.82 | down |
| MWSmce294 | Phenolic acids | 9346207 | 9686118 | 10008604 | 8398054 | 7863616 | 7638588 | 1.42 | 0.82 | down |
| mws0014 | Phenolic acids | 4376456 | 4548033 | 4440173 | 3291720 | 3655659 | 4116528 | 1.24 | 0.83 | down |
| Labn001995 | Phenolic acids | 755996 | 854245 | 724409 | 691990 | 556035 | 686530 | 1.11 | 0.83 | down |
| pmb3317 | Phenolic acids | 24294 | 26341 | 30209 | 22822 | 22059 | 22622 | 1.21 | 0.83 | down |
| pmb0752 | Phenolic acids | 1059613 | 1037367 | 1092837 | 931893 | 855781 | 900134 | 1.42 | 0.84 | down |
| Zmhn001358 | Phenolic acids | 460144 | 551649 | 547025 | 491549 | 407476 | 441089 | 1.03 | 0.86 | down |
| MWSmce482 | Phenolic acids | 1239535 | 1215704 | 1173971 | 1050045 | 1087051 | 1020268 | 1.42 | 0.87 | down |
| MWSmce389 | Phenolic acids | 1346957 | 1253575 | 1277183 | 1143367 | 1139578 | 1109557 | 1.42 | 0.87 | down |
| pmb3074 | Phenolic acids | 124757 | 107480 | 113438 | 97948 | 109671 | 95740 | 1.10 | 0.88 | down |
| pmn001518 | Phenolic acids | 164956 | 150518 | 167472 | 157528 | 137240 | 136846 | 1.06 | 0.89 | down |
| pmb2940 | Phenolic acids | 20360842 | 20022790 | 20015831 | 18731391 | 19443489 | 17093818 | 1.14 | 0.92 | down |
| pmb3068 | Phenolic acids | 243699 | 243447 | 231064 | 216123 | 217120 | 236343 | 1.08 | 0.93 | down |
| HJN003 | Phenolic acids | 2352052 | 2672609 | 2460598 | 2826423 | 2863382 | 2559620 | 1.03 | 1.10 | up |
| MWSmce083 | Phenolic acids | 67996 | 67204 | 64237 | 75158 | 73038 | 72923 | 1.40 | 1.11 | up |
| pmb2620 | Phenolic acids | 59678 | 53563 | 49746 | 62688 | 60556 | 63602 | 1.18 | 1.15 | up |
| pme0281 | Phenolic acids | 349982 | 398175 | 351732 | 465492 | 436108 | 404565 | 1.24 | 1.19 | up |
| Zmhn001420 | Phenolic acids | 349982 | 398175 | 351732 | 465492 | 436108 | 404565 | 1.24 | 1.19 | up |
| mad2085 | Phenolic acids | 86554 | 75606 | 89319 | 94317 | 113323 | 94800 | 1.14 | 1.20 | up |
| Zmgn004894 | Phenolic acids | 68179 | 61932 | 62529 | 82660 | 67902 | 82750 | 1.19 | 1.21 | up |
| Lmrgn03360 | Phenolic acids | 225535 | 233335 | 215356 | 262413 | 288135 | 272467 | 1.41 | 1.22 | up |
| ML10179289 | Phenolic acids | 5748318 | 5314401 | 5392249 | 5492014 | 7216662 | 7924559 | 1.04 | 1.25 | up |
| pme1439 | Phenolic acids | 307090 | 250867 | 258846 | 300688 | 321703 | 406031 | 1.07 | 1.26 | up |
| pme1292 | Phenolic acids | 107231 | 95849 | 116545 | 122246 | 134226 | 148703 | 1.24 | 1.27 | up |
| Wbmn005986 | Phenolic acids | 97395315 | 97762453 | 81737883 | 108947066 | 120814899 | 121330322 | 1.30 | 1.27 | up |
| Wmzn002116 | Phenolic acids | 74155 | 89514 | 98965 | 118451 | 107572 | 107172 | 1.20 | 1.27 | up |
| Zmhn002422 | Phenolic acids | 2511844 | 2572227 | 2862921 | 3467031 | 3540862 | 3153959 | 1.38 | 1.28 | up |
| mws1150 | Phenolic acids | 4922946 | 3780467 | 4653048 | 5145584 | 5675254 | 6356097 | 1.18 | 1.29 | up |
| Hmhn003518 | Phenolic acids | 27693524 | 30064522 | 25561402 | 35831417 | 34936203 | 37494691 | 1.40 | 1.30 | up |
| Lmmn003663 | Phenolic acids | 2365789 | 2044616 | 2148746 | 2647056 | 3224855 | 2815286 | 1.33 | 1.32 | up |
| MWSslk149 | Phenolic acids | 67359 | 65143 | 82046 | 85352 | 96938 | 102484 | 1.27 | 1.33 | up |
| Labn003679 | Phenolic acids | 1304780 | 1255192 | 1194275 | 1623525 | 1616940 | 1747382 | 1.45 | 1.33 | up |
| Lmgp002593 | Phenolic acids | 2377390 | 2427120 | 1903462 | 3413200 | 2652450 | 2882120 | 1.20 | 1.33 | up |
| Jmbp006554 | Phenolic acids | 442205 | 558515 | 548743 | 651863 | 758346 | 686324 | 1.30 | 1.35 | up |
| Lmrn003000 | Phenolic acids | 120706 | 145509 | 135961 | 196085 | 170573 | 183957 | 1.38 | 1.37 | up |
| Zmhn002334 | Phenolic acids | 2994148 | 2575067 | 2940031 | 4079157 | 3671016 | 3928289 | 1.41 | 1.37 | up |
| pmn001671 | Phenolic acids | 146273 | 169557 | 173437 | 228917 | 221354 | 221335 | 1.42 | 1.37 | up |
| Zbfn002396 | Phenolic acids | 91606 | 98888 | 94364 | 138888 | 127103 | 136437 | 1.47 | 1.41 | up |
| pmn001690 | Phenolic acids | 274101 | 254601 | 250364 | 375396 | 392637 | 355943 | 1.47 | 1.44 | up |
| Lmmn000774 | Phenolic acids | 321333 | 326452 | 209183 | 533419 | 312140 | 413571 | 1.00 | 1.47 | up |
| pmn001459 | Phenolic acids | 111173 | 150670 | 125307 | 203601 | 179731 | 185757 | 1.35 | 1.47 | up |
| Wayn001856 | Phenolic acids | 5248321 | 4982519 | 5177092 | 7754343 | 7637497 | 7518359 | 1.49 | 1.49 | up |
| Lmbn004790 | Phenolic acids | 225388 | 167279 | 201926 | 333911 | 354595 | 199752 | 1.02 | 1.49 | up |
| Lmgp003989 | Phenolic acids | 309350 | 329738 | 291130 | 370529 | 590734 | 437945 | 1.22 | 1.50 | up |
| Lmsn003628 | Phenolic acids | 163824 | 148456 | 174877 | 248253 | 244240 | 244204 | 1.46 | 1.51 | up |
| Cmzp002057 | Phenolic acids | 2232539 | 2005240 | 2011489 | 2928160 | 3286546 | 3261550 | 1.46 | 1.52 | up |
| Jmwn002494 | Phenolic acids | 56352 | 61246 | 43039 | 86649 | 71661 | 86525 | 1.30 | 1.52 | up |
| Lmhn003240 | Phenolic acids | 39426 | 53764 | 41312 | 76568 | 64039 | 65099 | 1.33 | 1.53 | up |
| Hmhn003067 | Phenolic acids | 70831 | 31311 | 41284 | 79720 | 76893 | 68528 | 1.08 | 1.57 | up |
| pmn001672 | Phenolic acids | 102844 | 99546 | 124235 | 197294 | 138988 | 179933 | 1.31 | 1.58 | up |
| Lmsn000363 | Phenolic acids | 756162 | 742601 | 533863 | 677111 | 1201541 | 1375825 | 1.00 | 1.60 | up |
| Waxn004250 | Phenolic acids | 42740 | 43062 | 63161 | 63546 | 103655 | 83510 | 1.21 | 1.68 | up |
| Wasn006403 | Phenolic acids | 250506 | 388665 | 511350 | 754427 | 546151 | 649529 | 1.16 | 1.69 | up |
| Lmfn003035 | Phenolic acids | 3358766 | 3313498 | 3110528 | 5525936 | 5675255 | 5912150 | 1.49 | 1.75 | up |
| Wasn006882 | Phenolic acids | 460673 | 436295 | 467399 | 895306 | 713762 | 789894 | 1.46 | 1.76 | up |
| Lmbp000728 | Phenolic acids | 12610662 | 10349220 | 11125525 | 19088584 | 19875694 | 21615043 | 1.46 | 1.78 | up |
| MWS2070 | Phenolic acids | 20280 | 25004 | 25101 | 56911 | 42266 | 32106 | 1.29 | 1.87 | up |
| Lmmn001643 | Phenolic acids | 1126301 | 1258509 | 1187125 | 2601801 | 2546571 | 2112372 | 1.47 | 2.03 | up |
| MWSmce248 | Phenolic acids | 1126301 | 1258509 | 1187125 | 2601801 | 2546571 | 2112372 | 1.47 | 2.03 | up |
| Lmbn002648 | Phenolic acids | 1126301 | 1258509 | 1187125 | 2601801 | 2546571 | 2112372 | 1.47 | 2.03 | up |
| Li512115 | Phenolic acids | 279268 | 172882 | 222981 | 353731 | 584666 | 437154 | 1.31 | 2.04 | up |
| MWSslk216 | Phenolic acids | 176385 | 159162 | 185631 | 374323 | 384307 | 391575 | 1.49 | 2.21 | up |
| Hmbn002692 | Phenolic acids | 151884 | 180476 | 161935 | 422413 | 325103 | 345996 | 1.46 | 2.21 | up |
| Wasn004620 | Phenolic acids | 21260 | 10323 | 11739 | 31523 | 36259 | 37745 | 1.35 | 2.44 | up |
| Lasp010305 | Phenolic acids | 322710 | 334886 | 337999 | 869525 | 861484 | 805118 | 1.50 | 2.55 | up |
| Jmbn005456 | Phenolic acids | 131937 | 88188 | 62420 | 311570 | 209803 | 303663 | 1.37 | 2.92 | up |
| pmn001382 | Phenolic acids | 115955 | 74123 | 90894 | 369339 | 773497 | 468062 | 1.44 | 5.73 | up |
| pmn001384 | Phenolic acids | 115955 | 74123 | 90894 | 369339 | 773497 | 468062 | 1.44 | 5.73 | up |
| Wasn002508 | Others | 269987 | 252671 | 296845 | 113279 | 123178 | 171772 | 1.40 | 0.50 | down |
| Lsmp121707 | Others | 32011 | 21434 | 17290 | 15295 | 8670 | 12114 | 1.21 | 0.51 | down |
| Wcgp003276 | Others | 9083811 | 7503589 | 7900461 | 4854068 | 4570331 | 4645882 | 1.47 | 0.57 | down |
| Wafn004433 | Others | 24202 | 31127 | 37579 | 21675 | 21324 | 11530 | 1.13 | 0.59 | down |
| Zmgn000173 | Others | 182042 | 184096 | 207269 | 140783 | 78811 | 119733 | 1.26 | 0.59 | down |
| pmn001491 | Others | 84409 | 77029 | 67263 | 53234 | 50987 | 34576 | 1.29 | 0.61 | down |
| Lasp003143 | Others | 9472673 | 11338937 | 7771362 | 5691622 | 6154737 | 5507260 | 1.36 | 0.61 | down |
| Zmsp002859 | Others | 10618523 | 12330214 | 10835848 | 6422346 | 7212476 | 7400282 | 1.45 | 0.62 | down |
| Lmjp101403 | Others | 257635 | 150248 | 213101 | 127567 | 140109 | 128151 | 1.20 | 0.64 | down |
| Wafn004792 | Others | 117822 | 111984 | 127284 | 62273 | 82879 | 85729 | 1.35 | 0.65 | down |
| Latp003004 | Others | 5782289 | 5785850 | 5808889 | 3857493 | 3545581 | 3851903 | 1.49 | 0.65 | down |
| Lchp000218 | Others | 241937 | 344497 | 295082 | 148831 | 189486 | 234943 | 1.19 | 0.65 | down |
| Wcdp006912 | Others | 12808030 | 10195529 | 11156998 | 7778818 | 7791351 | 6813163 | 1.40 | 0.66 | down |
| mws4163 | Others | 56358 | 71886 | 89553 | 51964 | 33091 | 58021 | 1.05 | 0.66 | down |
| Wcsn010254 | Others | 354215 | 389771 | 325315 | 269790 | 229369 | 208043 | 1.37 | 0.66 | down |
| Lhmp122021 | Others | 846130 | 650960 | 741421 | 507574 | 490625 | 513510 | 1.39 | 0.68 | down |
| MWS1844 | Others | 323673 | 292205 | 306354 | 196382 | 222333 | 208272 | 1.46 | 0.68 | down |
| Wagp005869 | Others | 33064 | 40101 | 52216 | 25005 | 35951 | 24624 | 1.09 | 0.68 | down |
| Lmsp003511 | Others | 268389 | 222231 | 184033 | 149579 | 177293 | 143476 | 1.22 | 0.70 | down |
| pmb0952 | Others | 459203 | 609318 | 439316 | 376893 | 321806 | 354625 | 1.26 | 0.70 | down |
| Lhhp120823 | Others | 16358897 | 19623581 | 19328576 | 11627215 | 13474281 | 13693165 | 1.37 | 0.70 | down |
| pme0534 | Others | 11164370 | 14856341 | 12912980 | 8296350 | 9246975 | 9922633 | 1.31 | 0.71 | down |
| Ljmp121505 | Others | 5561164 | 5124716 | 5645972 | 3661625 | 3906890 | 4043559 | 1.46 | 0.71 | down |
| mws2523 | Others | 1492577 | 1114797 | 1154624 | 876837 | 772704 | 1053048 | 1.18 | 0.72 | down |
| Wbtn006721 | Others | 304721 | 268155 | 272418 | 200279 | 207341 | 200609 | 1.45 | 0.72 | down |
| Wagp005508 | Others | 2060935 | 2949049 | 2358461 | 1802193 | 1691455 | 1826191 | 1.24 | 0.72 | down |
| pme1261 | Others | 80124 | 90561 | 84624 | 55657 | 61872 | 69296 | 1.36 | 0.73 | down |
| Zmzn000079 | Others | 1419060 | 1120802 | 1302751 | 1022529 | 1032899 | 760136 | 1.19 | 0.73 | down |
| mws1090 | Others | 32592333 | 26489189 | 34315427 | 19805352 | 23145661 | 25941248 | 1.21 | 0.74 | down |
| Wcsn010349 | Others | 44480 | 51482 | 52678 | 33318 | 38829 | 38084 | 1.35 | 0.74 | down |
| Wcgp005566 | Others | 2703531 | 2511821 | 2612675 | 2007252 | 1936125 | 1897933 | 1.48 | 0.75 | down |
| Ymmg000006 | Others | 494799 | 482483 | 403356 | 376927 | 336338 | 346034 | 1.31 | 0.77 | down |
| pmf0440 | Others | 197488 | 166086 | 200974 | 165490 | 137181 | 134194 | 1.23 | 0.77 | down |
| Lmsn000381 | Others | 1958352 | 1340285 | 1785111 | 1240096 | 1478878 | 1245774 | 1.03 | 0.78 | down |
| mws5038 | Others | 1958352 | 1340285 | 1785111 | 1240096 | 1478878 | 1245774 | 1.03 | 0.78 | down |
| Lmlp002765 | Others | 731533 | 726723 | 668056 | 603671 | 545194 | 517201 | 1.37 | 0.78 | down |
| Wcfp002357 | Others | 718339 | 681892 | 685315 | 619060 | 475001 | 570544 | 1.23 | 0.80 | down |
| pmp001229 | Others | 13077762 | 12764785 | 12093594 | 9709513 | 9962031 | 10996710 | 1.39 | 0.81 | down |
| pme2125 | Others | 8396317 | 10346633 | 8671056 | 7257403 | 7616220 | 7565505 | 1.24 | 0.82 | down |
| Lsmp121622 | Others | 557633 | 510297 | 613728 | 468947 | 454417 | 487896 | 1.25 | 0.84 | down |
| Hmyp002315 | Others | 3216038 | 3527116 | 3346884 | 2827393 | 2757761 | 2985843 | 1.38 | 0.85 | down |
| Zmhn001746 | Others | 268429 | 286612 | 272400 | 236105 | 244550 | 227414 | 1.41 | 0.86 | down |
| Zmgn000447 | Others | 992203 | 1031541 | 1145177 | 977809 | 864919 | 934427 | 1.14 | 0.88 | down |
| Zmsn003840 | Others | 703667 | 775858 | 716730 | 642490 | 643623 | 642323 | 1.36 | 0.88 | down |
| Lmqn000351 | Others | 88156 | 93507 | 89686 | 77145 | 86785 | 85478 | 1.08 | 0.92 | down |
| pme2596 | Others | 211745 | 206034 | 204607 | 196849 | 193676 | 183852 | 1.30 | 0.92 | down |
| pmf0175 | Others | 1865702 | 1984425 | 1999838 | 2048332 | 2020682 | 2032094 | 1.03 | 1.04 | up |
| Wcfn003941 | Others | 609629 | 632958 | 559391 | 671064 | 712258 | 666971 | 1.26 | 1.14 | up |
| Lmdn005449 | Others | 84004 | 95148 | 87367 | 101731 | 104889 | 98131 | 1.28 | 1.14 | up |
| Zmdp000376 | Others | 1884486 | 2217384 | 1988196 | 2419373 | 2394953 | 2212475 | 1.19 | 1.15 | up |
| mws0628 | Others | 327809 | 390814 | 398052 | 437372 | 434397 | 418728 | 1.14 | 1.16 | up |
| Wcdp006247 | Others | 2348809 | 2892779 | 2516954 | 3236487 | 3145541 | 2936209 | 1.21 | 1.20 | up |
| ML10171848 | Others | 2846826 | 3332296 | 3082793 | 3847433 | 3968326 | 3342790 | 1.20 | 1.20 | up |
| pma1751 | Others | 4173847 | 4776892 | 5479574 | 5691619 | 5959108 | 6541596 | 1.20 | 1.26 | up |
| Wcgn003981 | Others | 2338924 | 2243547 | 2192824 | 2885677 | 3023400 | 2689574 | 1.43 | 1.27 | up |
| Wasn000977 | Others | 262212 | 210131 | 258101 | 317017 | 344131 | 277296 | 1.20 | 1.28 | up |
| Wchn003892 | Others | 2954704 | 2745527 | 2661490 | 3640104 | 3670574 | 3504803 | 1.45 | 1.29 | up |
| pmp001267 | Others | 590947 | 559888 | 547936 | 778438 | 716529 | 724956 | 1.45 | 1.31 | up |
| mws1593 | Others | 15089 | 15066 | 16805 | 18512 | 21144 | 22015 | 1.36 | 1.31 | up |
| Wcdp006741 | Others | 16330069 | 19400416 | 13989894 | 25103604 | 19534925 | 20899184 | 1.14 | 1.32 | up |
| Wchn004079 | Others | 27284931 | 26056609 | 26387885 | 34189213 | 35586086 | 36614166 | 1.48 | 1.33 | up |
| Zasn010461 | Others | 75980 | 88577 | 122719 | 120450 | 134169 | 131930 | 1.10 | 1.35 | up |
| Zmyn000108 | Others | 10143548 | 10768727 | 13435427 | 13679229 | 15548358 | 17979957 | 1.21 | 1.37 | up |
| Wbmp003302 | Others | 685025 | 577973 | 640717 | 807866 | 872854 | 959407 | 1.38 | 1.39 | up |
| MWSHY0141 | Others | 1067865 | 1150397 | 943368 | 1464388 | 1468178 | 1461860 | 1.42 | 1.39 | up |
| Lmbn005172 | Others | 115880 | 153233 | 128359 | 187046 | 192194 | 174649 | 1.34 | 1.39 | up |
| Zmcp102206 | Others | 58653 | 64868 | 74540 | 96431 | 91079 | 88726 | 1.37 | 1.39 | up |
| Wmmp000181 | Others | 87942 | 104165 | 87904 | 142723 | 133217 | 115561 | 1.34 | 1.40 | up |
| pme2529 | Others | 88153 | 115320 | 88825 | 126688 | 119195 | 164889 | 1.18 | 1.41 | up |
| pme2165 | Others | 4369784 | 3622011 | 2211369 | 4755851 | 5115615 | 4647882 | 1.03 | 1.42 | up |
| MWSmce417 | Others | 2022069 | 1840048 | 1915926 | 3137678 | 2843911 | 2339184 | 1.34 | 1.44 | up |
| Lcsn000560 | Others | 2053078 | 1605016 | 1978833 | 2890839 | 2392988 | 2878288 | 1.33 | 1.45 | up |
| Lmsn003522 | Others | 1475701 | 1398039 | 1469311 | 2072638 | 2081826 | 2148171 | 1.49 | 1.45 | up |
| Lmqn000432 | Others | 1031399 | 1340761 | 874815 | 1759275 | 1735475 | 1502578 | 1.28 | 1.54 | up |
| pme1383 | Others | 286785 | 323180 | 273440 | 409595 | 514204 | 447588 | 1.40 | 1.55 | up |
| Labp005050 | Others | 110045 | 144994 | 172645 | 303800 | 215520 | 166731 | 1.09 | 1.60 | up |
| Hmcn000192 | Others | 5546706 | 3253792 | 5142711 | 6862376 | 6199208 | 9406674 | 1.14 | 1.61 | up |
| Lmbn002737 | Others | 205283 | 229461 | 186021 | 337576 | 333327 | 341968 | 1.46 | 1.63 | up |
| Zmyn000083 | Others | 1296345 | 1073414 | 1145635 | 1858145 | 2284400 | 1727406 | 1.40 | 1.67 | up |
| pme3511 | Others | 35955 | 59889 | 66641 | 69661 | 105589 | 96472 | 1.14 | 1.67 | up |
| pmb2653 | Others | 12699 | 18488 | 13580 | 21946 | 19647 | 39312 | 1.13 | 1.81 | up |
| pme3163 | Others | 339540 | 376207 | 451785 | 873028 | 401645 | 838753 | 1.07 | 1.81 | up |
| Wcdp000970 | Others | 11158929 | 9105693 | 8981308 | 17542904 | 19350267 | 19034061 | 1.46 | 1.91 | up |
| Wcjp000888 | Others | 23777661 | 17962461 | 19041613 | 34500995 | 41196645 | 41266104 | 1.43 | 1.92 | up |
| Lmbn002644 | Others | 146550 | 180277 | 158935 | 325968 | 315512 | 343272 | 1.48 | 2.03 | up |
| pma0134 | Others | 1644638 | 1656109 | 1647982 | 3409493 | 3662496 | 3090838 | 1.49 | 2.05 | up |
| Zmpn000199 | Others | 9442893 | 6992310 | 7997545 | 11854020 | 11654316 | 26856265 | 1.12 | 2.06 | up |
| Wafn003633 | Others | 9170 | 11457 | 3340 | 11823 | 24320 | 14955 | 1.04 | 2.13 | up |
| pme1014 | Others | 335327 | 459023 | 451491 | 708119 | 1091456 | 900677 | 1.38 | 2.17 | up |
| pmp000293 | Others | 1379065 | 1238651 | 1409600 | 3454055 | 3528192 | 3466259 | 1.50 | 2.59 | up |
| Wagp009011 | Others | 128250 | 141617 | 131404 | 348053 | 337378 | 414863 | 1.49 | 2.74 | up |
| Lmzn003953 | Others | 13228 | 18641 | 18020 | 67288 | 38846 | 48836 | 1.42 | 3.11 | up |
| mws1359 | Others | 5835 | 6421 | 7588 | 19021 | 22117 | 22417 | 1.48 | 3.20 | up |
| Wagp009250 | Others | 472020 | 392238 | 406208 | 1470093 | 1401074 | 1465863 | 1.49 | 3.41 | up |
| mws1603 | Lignans and Coumarins | 463831 | 467878 | 485567 | 209822 | 214245 | 201436 | 1.50 | 0.44 | down |
| HX1341 | Lignans and Coumarins | 7933671 | 8967592 | 7000368 | 3908112 | 3393684 | 3775484 | 1.47 | 0.46 | down |
| Lahp003986 | Lignans and Coumarins | 1188441 | 1461590 | 1230610 | 618291 | 701613 | 632050 | 1.47 | 0.50 | down |
| Rfmb26201 | Lignans and Coumarins | 50436 | 110248 | 83140 | 64512 | 39637 | 21016 | 1.01 | 0.51 | down |
| Lhmp121010 | Lignans and Coumarins | 206313 | 196601 | 148652 | 127910 | 81867 | 96226 | 1.31 | 0.55 | down |
| Lajp004644 | Lignans and Coumarins | 312589 | 204986 | 245057 | 126354 | 185339 | 131619 | 1.27 | 0.58 | down |
| Hmcn002743 | Lignans and Coumarins | 22033 | 17411 | 20995 | 9299 | 15016 | 13139 | 1.26 | 0.62 | down |
| pmb4777 | Lignans and Coumarins | 167579 | 153610 | 181525 | 142748 | 89464 | 93913 | 1.24 | 0.65 | down |
| pmn001375 | Lignans and Coumarins | 57229 | 81570 | 55377 | 48256 | 39864 | 46462 | 1.19 | 0.69 | down |
| MWSmce617 | Lignans and Coumarins | 197966 | 177955 | 185105 | 117641 | 148352 | 127149 | 1.39 | 0.70 | down |
| Zmln002252 | Lignans and Coumarins | 347103 | 327015 | 373218 | 263266 | 255885 | 220118 | 1.40 | 0.71 | down |
| Lmwp102713 | Lignans and Coumarins | 181654 | 186918 | 234797 | 148928 | 113983 | 164330 | 1.18 | 0.71 | down |
| Wbmn002637 | Lignans and Coumarins | 16701834 | 17326450 | 13331827 | 11364511 | 10751389 | 11726224 | 1.33 | 0.71 | down |
| pmp000284 | Lignans and Coumarins | 112098 | 104133 | 90500 | 73629 | 77016 | 70054 | 1.38 | 0.72 | down |
| MWSmce265 | Lignans and Coumarins | 1055618 | 1072437 | 1020046 | 705726 | 773130 | 800093 | 1.46 | 0.72 | down |
| Lmbp003208 | Lignans and Coumarins | 245620 | 210321 | 272663 | 179058 | 149657 | 203507 | 1.21 | 0.73 | down |
| Lmbp102601 | Lignans and Coumarins | 10131574 | 12411987 | 11295218 | 6863968 | 8809531 | 9289900 | 1.22 | 0.74 | down |
| Lmjp003090 | Lignans and Coumarins | 375263 | 317495 | 305879 | 236634 | 216863 | 296659 | 1.19 | 0.75 | down |
| Hmhn004708 | Lignans and Coumarins | 345176 | 268247 | 308312 | 244590 | 217879 | 232517 | 1.30 | 0.75 | down |
| Lcyp000672 | Lignans and Coumarins | 686005 | 775009 | 878884 | 627122 | 640125 | 505016 | 1.20 | 0.76 | down |
| Wbwp010459 | Lignans and Coumarins | 73120 | 53788 | 72970 | 51200 | 54307 | 57317 | 1.01 | 0.81 | down |
| HX1361 | Lignans and Coumarins | 29565954 | 29363161 | 32560964 | 26741118 | 22535513 | 25428188 | 1.29 | 0.82 | down |
| Hmhp002580 | Lignans and Coumarins | 494469 | 526140 | 473553 | 401783 | 451138 | 431893 | 1.29 | 0.86 | down |
| pmb0235 | Lignans and Coumarins | 531533 | 579936 | 593153 | 480055 | 503154 | 484430 | 1.35 | 0.86 | down |
| Lmjp101401 | Lignans and Coumarins | 7084290 | 6293912 | 6466153 | 5211606 | 6228033 | 5704622 | 1.14 | 0.86 | down |
| Wagp009817 | Lignans and Coumarins | 5319208 | 5339534 | 5648125 | 5669735 | 5811963 | 6042958 | 1.20 | 1.07 | up |
| pmb1207 | Lignans and Coumarins | 822822 | 921786 | 886588 | 976483 | 935130 | 931773 | 1.10 | 1.08 | up |
| Wbwp008548 | Lignans and Coumarins | 16477278 | 16823764 | 17119473 | 18376166 | 17625185 | 19568336 | 1.25 | 1.10 | up |
| Wagp010109 | Lignans and Coumarins | 2431945 | 2727327 | 2869784 | 2755466 | 3183420 | 3280777 | 1.03 | 1.15 | up |
| Lahp004665 | Lignans and Coumarins | 319248 | 361553 | 325589 | 436305 | 393964 | 368006 | 1.22 | 1.19 | up |
| MWSmce388 | Lignans and Coumarins | 267504 | 350712 | 305163 | 341124 | 415688 | 362150 | 1.06 | 1.21 | up |
| pmb1178 | Lignans and Coumarins | 671315 | 738681 | 790165 | 743418 | 928342 | 1003871 | 1.02 | 1.22 | up |
| HX1366 | Lignans and Coumarins | 4183484 | 2908939 | 3583951 | 4523123 | 4463933 | 4070194 | 1.04 | 1.22 | up |
| Cmyn001328 | Lignans and Coumarins | 52630 | 63700 | 44978 | 64762 | 66086 | 71944 | 1.12 | 1.26 | up |
| mws1015 | Lignans and Coumarins | 52630 | 63700 | 44978 | 64762 | 66086 | 71944 | 1.12 | 1.26 | up |
| Wagp008739 | Lignans and Coumarins | 55910 | 42383 | 47613 | 57395 | 71109 | 61366 | 1.19 | 1.30 | up |
| Wagp004859 | Lignans and Coumarins | 315166 | 376879 | 230186 | 459056 | 357405 | 411035 | 1.02 | 1.33 | up |
| zjgp122320 | Lignans and Coumarins | 3993807 | 4249621 | 5168553 | 6264963 | 6002756 | 6000729 | 1.34 | 1.36 | up |
| Zmpn002553 | Lignans and Coumarins | 50617 | 45293 | 59181 | 61197 | 77081 | 73059 | 1.25 | 1.36 | up |
| Lmbn001162 | Lignans and Coumarins | 44769 | 40854 | 40836 | 59097 | 51905 | 64093 | 1.38 | 1.38 | up |
| Wagp005475 | Lignans and Coumarins | 325196 | 256065 | 382495 | 425463 | 474070 | 451142 | 1.24 | 1.40 | up |
| HX1398 | Lignans and Coumarins | 115980 | 88167 | 116501 | 145271 | 158894 | 148415 | 1.32 | 1.41 | up |
| MWSmce200 | Lignans and Coumarins | 341927 | 352540 | 363138 | 561770 | 488496 | 483213 | 1.45 | 1.45 | up |
| Zmhn002291 | Lignans and Coumarins | 45801 | 49635 | 41363 | 59471 | 75263 | 65285 | 1.36 | 1.46 | up |
| Wagp006657 | Lignans and Coumarins | 176969 | 156996 | 167244 | 247485 | 242535 | 270986 | 1.46 | 1.52 | up |
| Cmmn005231 | Lignans and Coumarins | 99304 | 122142 | 112821 | 163264 | 154735 | 191099 | 1.39 | 1.52 | up |
| Hmqn002332 | Lignans and Coumarins | 118095 | 141131 | 158497 | 180279 | 249337 | 209079 | 1.29 | 1.53 | up |
| Qmzp101825 | Lignans and Coumarins | 28725 | 23739 | 26896 | 44048 | 42347 | 41979 | 1.46 | 1.62 | up |
| Wagp005148 | Lignans and Coumarins | 338470 | 295065 | 354523 | 542594 | 475716 | 605549 | 1.41 | 1.64 | up |
| MW0010655 | Lignans and Coumarins | 450960 | 571847 | 392524 | 802699 | 806889 | 803558 | 1.39 | 1.71 | up |
| Cmpp005475 | Lignans and Coumarins | 853780 | 905787 | 939006 | 1366451 | 1527522 | 1719775 | 1.45 | 1.71 | up |
| Zmcp001750 | Lignans and Coumarins | 162756 | 231570 | 207882 | 303809 | 441329 | 327054 | 1.32 | 1.78 | up |
| Lajp008026 | Lignans and Coumarins | 41096 | 30623 | 21913 | 66248 | 51294 | 51623 | 1.26 | 1.81 | up |
| Wagp009556 | Lignans and Coumarins | 27989 | 19608 | 14324 | 30185 | 51722 | 33479 | 1.17 | 1.86 | up |
| Qmzp101810 | Lignans and Coumarins | 158425 | 238540 | 108333 | 320014 | 273398 | 360382 | 1.22 | 1.89 | up |
| Wagp007597 | Lignans and Coumarins | 143685 | 109269 | 116792 | 226181 | 261156 | 227602 | 1.44 | 1.93 | up |
| Wagp012431 | Lignans and Coumarins | 29453 | 31885 | 38840 | 50555 | 77186 | 71442 | 1.37 | 1.99 | up |
| HJN083 | Lignans and Coumarins | 85769 | 85258 | 75144 | 160419 | 180531 | 156216 | 1.48 | 2.02 | up |
| Wagp009246 | Lignans and Coumarins | 159014 | 134963 | 99635 | 332355 | 311621 | 293678 | 1.43 | 2.38 | up |
| HX1390 | Lignans and Coumarins | 139507 | 154137 | 144450 | 357394 | 336649 | 366271 | 1.50 | 2.42 | up |
| Wagp012906 | Lignans and Coumarins | 48924 | 37120 | 46655 | 95839 | 143934 | 91318 | 1.41 | 2.50 | up |
| Wagp005635 | Lignans and Coumarins | 7489 | 6955 | 10550 | 34318 | 16054 | 14260 | 1.24 | 2.59 | up |
| Lmjp101205 | Lignans and Coumarins | 382383 | 367212 | 361472 | 1029058 | 933120 | 946841 | 1.50 | 2.62 | up |
| Cmpp007452 | Lignans and Coumarins | 336497 | 307854 | 287643 | 857134 | 760680 | 870157 | 1.49 | 2.67 | up |
| Wagp005741 | Lignans and Coumarins | 207305 | 115199 | 117964 | 396202 | 371252 | 453661 | 1.40 | 2.77 | up |
| Lajp010752 | Lignans and Coumarins | 321965 | 303248 | 299519 | 904078 | 850152 | 861063 | 1.50 | 2.83 | up |
| Wagp006873 | Lignans and Coumarins | 225792 | 240385 | 253685 | 645710 | 736987 | 678399 | 1.49 | 2.86 | up |
| Cmpp007319 | Lignans and Coumarins | 331610 | 332113 | 293504 | 891274 | 980686 | 924585 | 1.49 | 2.92 | up |
| Walp004391 | Lignans and Coumarins | 112203 | 97224 | 58254 | 305412 | 226291 | 253412 | 1.40 | 2.93 | up |
| Wagp006865 | Lignans and Coumarins | 180800 | 171209 | 162266 | 508369 | 498194 | 532309 | 1.50 | 2.99 | up |
| Wagp005750 | Lignans and Coumarins | 11555 | 8799 | 16393 | 18374 | 37324 | 55015 | 1.22 | 3.01 | up |
| Lajp011784 | Lignans and Coumarins | 55442 | 41313 | 43877 | 168669 | 143243 | 117520 | 1.46 | 3.05 | up |
| Wagp005832 | Lignans and Coumarins | 700220 | 749363 | 741308 | 2429215 | 2267008 | 2356864 | 1.50 | 3.22 | up |
| Lajp005829 | Lignans and Coumarins | 56745 | 19925 | 37301 | 89364 | 205155 | 72575 | 1.19 | 3.22 | up |
| Cmpp008880 | Lignans and Coumarins | 1122092 | 1136274 | 1066790 | 3661605 | 3682849 | 3673596 | 1.50 | 3.31 | up |
| Lajp007234 | Lignans and Coumarins | 85599 | 60514 | 71234 | 248966 | 238520 | 234088 | 1.48 | 3.32 | up |
| Lmjp101101 | Lignans and Coumarins | 400914 | 313846 | 349615 | 1249952 | 1170506 | 1139600 | 1.49 | 3.34 | up |
| Lajp008902 | Lignans and Coumarins | 380217 | 340390 | 347519 | 1275423 | 1109855 | 1279392 | 1.49 | 3.43 | up |
| Lhqp101807 | Lignans and Coumarins | 908835 | 836668 | 798261 | 2974119 | 2980905 | 3125655 | 1.50 | 3.57 | up |
| Wagp005836 | Lignans and Coumarins | 760797 | 853292 | 837132 | 2963319 | 2753227 | 3036912 | 1.50 | 3.57 | up |
| Lmjp101102 | Lignans and Coumarins | 350321 | 318412 | 326333 | 1104890 | 1092175 | 1395730 | 1.49 | 3.61 | up |
| Wagp009522 | Lignans and Coumarins | 350321 | 318412 | 326333 | 1104890 | 1092175 | 1395730 | 1.49 | 3.61 | up |
| Wagp008361 | Lignans and Coumarins | 53707 | 56939 | 60028 | 210233 | 215311 | 195246 | 1.50 | 3.64 | up |
| Wagp012423 | Lignans and Coumarins | 59824 | 38454 | 48188 | 177917 | 194422 | 174105 | 1.47 | 3.73 | up |
| Wagp012414 | Lignans and Coumarins | 318328 | 313338 | 325955 | 1322972 | 1245953 | 1204596 | 1.50 | 3.94 | up |
| Wagp012715 | Lignans and Coumarins | 344493 | 309389 | 299824 | 1319318 | 1379536 | 1275511 | 1.50 | 4.17 | up |
| Wagp010375 | Lignans and Coumarins | 97733 | 65482 | 96383 | 412458 | 385117 | 385310 | 1.48 | 4.56 | up |
| Wagp012904 | Lignans and Coumarins | 19773 | 6885 | 17475 | 101693 | 52082 | 74350 | 1.37 | 5.17 | up |
| Wagp010335 | Lignans and Coumarins | 35298 | 28804 | 30053 | 200827 | 167494 | 156973 | 1.49 | 5.58 | up |
| MWSmce082 | Lignans and Coumarins | 110663 | 127400 | 182195 | 789794 | 843027 | 857778 | 1.48 | 5.93 | up |
| Cmbp003769 | Alkaloids | 9545409 | 8640708 | 17489965 | 6722195 | 4411548 | 6406023 | 1.19 | 0.49 | down |
| MWS3020 | Alkaloids | 281957 | 312989 | 221440 | 139636 | 92836 | 229980 | 1.12 | 0.57 | down |
| MW0000332 | Alkaloids | 234872 | 393284 | 264445 | 107852 | 290300 | 117297 | 1.00 | 0.58 | down |
| pmn001727 | Alkaloids | 533051 | 782675 | 559670 | 366693 | 388758 | 396612 | 1.33 | 0.61 | down |
| Zmnn009241 | Alkaloids | 4353 | 6987 | 7617 | 3355 | 4331 | 4413 | 1.11 | 0.64 | down |
| Wagp003518 | Alkaloids | 46616 | 43012 | 58740 | 34740 | 28860 | 34176 | 1.32 | 0.66 | down |
| pmb1912 | Alkaloids | 16946759 | 18028081 | 15821855 | 11273169 | 11684695 | 12966150 | 1.43 | 0.71 | down |
| Lwhp010635 | Alkaloids | 86900 | 97522 | 59589 | 66923 | 49198 | 57330 | 1.03 | 0.71 | down |
| Lmhp002501 | Alkaloids | 192337 | 166284 | 210927 | 154800 | 137407 | 125163 | 1.29 | 0.73 | down |
| Wagp001741 | Alkaloids | 277376 | 281554 | 367069 | 224272 | 252327 | 206223 | 1.22 | 0.74 | down |
| Cmbp003825 | Alkaloids | 1457288 | 1155830 | 1434533 | 935519 | 978537 | 1086574 | 1.29 | 0.74 | down |
| pmb0770 | Alkaloids | 1115102 | 1166009 | 901939 | 797153 | 734852 | 851045 | 1.27 | 0.75 | down |
| pma0101 | Alkaloids | 180530 | 194834 | 187975 | 123608 | 153152 | 153458 | 1.32 | 0.76 | down |
| MWStz058 | Alkaloids | 115669 | 121028 | 103919 | 97951 | 77834 | 92779 | 1.24 | 0.79 | down |
| Lmmp002080 | Alkaloids | 6729690 | 7243660 | 6614999 | 5035152 | 5602755 | 5834472 | 1.36 | 0.80 | down |
| Wafp003720 | Alkaloids | 206759 | 193189 | 229258 | 154148 | 185736 | 176792 | 1.20 | 0.82 | down |
| Lmgp001898 | Alkaloids | 381932 | 402175 | 433019 | 363724 | 343034 | 295234 | 1.21 | 0.82 | down |
| Wagp001008 | Alkaloids | 12158301 | 11808882 | 13878136 | 10234708 | 10553346 | 11138542 | 1.25 | 0.84 | down |
| Lmmp002013 | Alkaloids | 6304634 | 7155307 | 7717488 | 5983877 | 5341279 | 6573451 | 1.06 | 0.85 | down |
| Wbkp008219 | Alkaloids | 794307 | 972796 | 919637 | 755617 | 844644 | 682225 | 1.03 | 0.85 | down |
| Wbsp001938 | Alkaloids | 5112327 | 4741409 | 4156959 | 4073346 | 4065031 | 3854383 | 1.16 | 0.86 | down |
| Lwhp010633 | Alkaloids | 301628 | 295375 | 309425 | 257810 | 252853 | 267019 | 1.45 | 0.86 | down |
| Wagp002534 | Alkaloids | 12737688 | 13994864 | 13724723 | 13083050 | 10454710 | 11501250 | 1.08 | 0.87 | down |
| Lmlp003161 | Alkaloids | 8591363 | 7628284 | 6972108 | 6382199 | 6891121 | 6878622 | 1.09 | 0.87 | down |
| MWSmce338 | Alkaloids | 419282 | 377036 | 325747 | 331463 | 319403 | 324932 | 1.01 | 0.87 | down |
| Wagp001908 | Alkaloids | 15765309 | 16141212 | 16004754 | 12720184 | 14140113 | 14972513 | 1.23 | 0.87 | down |
| Lsmp121605 | Alkaloids | 987871 | 903570 | 923398 | 881837 | 894582 | 882433 | 1.08 | 0.94 | down |
| Wasn002329 | Alkaloids | 716092 | 708625 | 696176 | 808980 | 718355 | 762462 | 1.10 | 1.08 | up |
| Zblp004317 | Alkaloids | 3078251 | 3629056 | 3317786 | 3738315 | 3607502 | 3642350 | 1.04 | 1.10 | up |
| Lmyp003387 | Alkaloids | 543739 | 590198 | 522139 | 620493 | 639430 | 656685 | 1.32 | 1.16 | up |
| pme2693 | Alkaloids | 491330 | 465270 | 432903 | 514960 | 508564 | 586222 | 1.17 | 1.16 | up |
| Lmhp005872 | Alkaloids | 69232 | 74268 | 73795 | 84297 | 77821 | 97353 | 1.17 | 1.19 | up |
| pmb0504 | Alkaloids | 351865 | 337583 | 356698 | 499466 | 380746 | 374156 | 1.01 | 1.20 | up |
| Layp000971 | Alkaloids | 2616764 | 3117562 | 3142741 | 3775743 | 4021808 | 3318899 | 1.21 | 1.25 | up |
| pmb0484 | Alkaloids | 5274705 | 6497592 | 6900261 | 7373001 | 7514546 | 8763897 | 1.16 | 1.27 | up |
| pme2268 | Alkaloids | 10476402 | 11228832 | 11499090 | 11671930 | 14825542 | 15851652 | 1.16 | 1.28 | up |
| pmb0501 | Alkaloids | 379679 | 404010 | 376300 | 532640 | 485649 | 486700 | 1.44 | 1.30 | up |
| Wagp001323 | Alkaloids | 1581314 | 1618891 | 1465646 | 2087230 | 1933139 | 2065539 | 1.44 | 1.30 | up |
| Lmxn006423 | Alkaloids | 18691 | 15546 | 17134 | 18559 | 22823 | 26039 | 1.15 | 1.31 | up |
| pmp001083 | Alkaloids | 818839 | 806053 | 706058 | 891261 | 1134122 | 1099379 | 1.28 | 1.34 | up |
| Hahp000801 | Alkaloids | 2589370 | 2870210 | 2805859 | 3725607 | 3697612 | 3793652 | 1.47 | 1.36 | up |
| mws1383 | Alkaloids | 239783 | 218053 | 251440 | 287662 | 293971 | 387086 | 1.24 | 1.37 | up |
| MWSmce548 | Alkaloids | 1041438 | 872970 | 1094042 | 1204698 | 1666227 | 1339628 | 1.22 | 1.40 | up |
| pmp001287 | Alkaloids | 5971366 | 4594837 | 5711580 | 8004562 | 6968101 | 7837350 | 1.32 | 1.40 | up |
| Lmqp002784 | Alkaloids | 54159 | 60281 | 67086 | 93054 | 84003 | 79880 | 1.37 | 1.42 | up |
| YC512115 | Alkaloids | 119114 | 114994 | 119914 | 139106 | 180937 | 202316 | 1.29 | 1.48 | up |
| pma3649 | Alkaloids | 40303065 | 42975668 | 37808005 | 56643156 | 57319426 | 67660995 | 1.42 | 1.50 | up |
| MWStz070 | Alkaloids | 4288128 | 4940715 | 4761627 | 7322186 | 6541387 | 7208476 | 1.45 | 1.51 | up |
| pmb0782 | Alkaloids | 1144566 | 1357531 | 1181218 | 1988331 | 1830715 | 1750788 | 1.43 | 1.51 | up |
| Hmtp000776 | Alkaloids | 132117 | 116112 | 66694 | 129966 | 167293 | 179081 | 1.04 | 1.51 | up |
| pmp001198 | Alkaloids | 918796 | 986216 | 979238 | 1433622 | 1574942 | 1470665 | 1.48 | 1.55 | up |
| pmp001214 | Alkaloids | 65458 | 46876 | 43505 | 75721 | 106421 | 65471 | 1.15 | 1.59 | up |
| Zasp102439 | Alkaloids | 4147446 | 4298284 | 4497620 | 5915451 | 7425538 | 7230724 | 1.43 | 1.59 | up |
| Lmlp001118 | Alkaloids | 266252 | 316409 | 183698 | 399701 | 472046 | 347985 | 1.19 | 1.59 | up |
| pme1002 | Alkaloids | 75641 | 70166 | 57581 | 107256 | 103700 | 113618 | 1.41 | 1.60 | up |
| HJKP000649 | Alkaloids | 4363687 | 4490748 | 4460696 | 7066360 | 7384781 | 7042755 | 1.50 | 1.61 | up |
| Lmxp000429 | Alkaloids | 2667193 | 2332222 | 2709302 | 4291060 | 4180266 | 4131206 | 1.47 | 1.63 | up |
| Lmrn003201 | Alkaloids | 20793 | 11731 | 15140 | 26689 | 23928 | 27374 | 1.25 | 1.64 | up |
| Zmsp000878 | Alkaloids | 1989044 | 2609887 | 2556417 | 4064430 | 3962592 | 3790409 | 1.42 | 1.65 | up |
| Wmtn012065 | Alkaloids | 151025 | 163043 | 171883 | 343699 | 218138 | 247464 | 1.30 | 1.67 | up |
| Lmgp000659 | Alkaloids | 4234152 | 4213296 | 4453399 | 6895032 | 7202054 | 7642307 | 1.49 | 1.69 | up |
| MWSmce089 | Alkaloids | 61914 | 94963 | 99837 | 135432 | 162720 | 147817 | 1.31 | 1.74 | up |
| mws0677 | Alkaloids | 98096 | 52416 | 48234 | 127414 | 110345 | 118763 | 1.22 | 1.79 | up |
| pmp001245 | Alkaloids | 54876 | 18075 | 55431 | 79920 | 66148 | 96791 | 1.04 | 1.89 | up |
| Wbmp002283 | Alkaloids | 4403244 | 3956183 | 4387575 | 8658328 | 7661078 | 7945554 | 1.48 | 1.90 | up |
| pmb1096 | Alkaloids | 3538868 | 1483171 | 3997733 | 5375813 | 6074137 | 5931367 | 1.14 | 1.93 | up |
| pmb0769 | Alkaloids | 201815 | 158399 | 110605 | 313263 | 293564 | 305612 | 1.34 | 1.94 | up |
| Wcjp002598 | Alkaloids | 6233601 | 5820553 | 6709964 | 14188503 | 11050034 | 11169638 | 1.45 | 1.94 | up |
| Zmbp002538 | Alkaloids | 4867620 | 4338114 | 5230388 | 9630200 | 9986576 | 9386361 | 1.48 | 2.01 | up |
| mws1433 | Alkaloids | 25383 | 27796 | 23364 | 51028 | 67787 | 43873 | 1.41 | 2.13 | up |
| Lahp002608 | Alkaloids | 1682093 | 1792034 | 1562979 | 4064075 | 3175800 | 3466671 | 1.47 | 2.13 | up |
| Hmmp002124 | Alkaloids | 4135003 | 3918577 | 3459258 | 9099391 | 7665716 | 8132434 | 1.47 | 2.16 | up |
| pmb0037 | Alkaloids | 4897648 | 4514972 | 4941207 | 11460954 | 6788383 | 12836804 | 1.32 | 2.17 | up |
| pme2244 | Alkaloids | 98759 | 125296 | 89798 | 218041 | 258463 | 233000 | 1.45 | 2.26 | up |
| Smcp001137 | Alkaloids | 1600922 | 1742029 | 1545703 | 3631860 | 3307177 | 4129928 | 1.48 | 2.26 | up |
| Lahp003147 | Alkaloids | 61685 | 209553 | 34946 | 216619 | 220743 | 262280 | 1.08 | 2.29 | up |
| Hmgp002327 | Alkaloids | 1803597 | 1495312 | 1522759 | 4023948 | 3377674 | 3617980 | 1.47 | 2.29 | up |
| Wagp004424 | Alkaloids | 21674 | 30080 | 19910 | 62923 | 53140 | 49839 | 1.42 | 2.31 | up |
| pmb0818 | Alkaloids | 427504 | 651983 | 584327 | 1361202 | 1123248 | 1388910 | 1.42 | 2.33 | up |
| Hmmp001310 | Alkaloids | 1489200 | 1586207 | 1495327 | 3838982 | 3642796 | 3840814 | 1.50 | 2.48 | up |
| Ladp003640 | Alkaloids | 23340 | 10384 | 11885 | 38522 | 23340 | 56277 | 1.20 | 2.59 | up |
| MWSmce709 | Alkaloids | 669429 | 880573 | 701282 | 2090825 | 2074207 | 1811090 | 1.47 | 2.65 | up |
| Zblp005777 | Alkaloids | 14697 | 10201 | 28340 | 39389 | 69766 | 50281 | 1.29 | 2.99 | up |
| MWSHC2028 | Alkaloids | 6314806 | 4953974 | 5553379 | 16976008 | 18930430 | 18582273 | 1.49 | 3.24 | up |
| pme0122 | Amino acids and derivatives | 713551 | 302559 | 313112 | 185030 | 128640 | 176363 | 1.26 | 0.37 | down |
| Zbqn004207 | Amino acids and derivatives | 391210 | 403561 | 385984 | 190814 | 177864 | 186858 | 1.50 | 0.47 | down |
| pmp001257 | Amino acids and derivatives | 54235 | 29621 | 34562 | 26031 | 19356 | 16639 | 1.22 | 0.52 | down |
| pme2743 | Amino acids and derivatives | 616245 | 569162 | 528780 | 376128 | 409959 | 261238 | 1.30 | 0.61 | down |
| mws0217 | Amino acids and derivatives | 162251 | 247120 | 261735 | 148711 | 122583 | 161176 | 1.17 | 0.64 | down |
| mws0629 | Amino acids and derivatives | 351924 | 433151 | 331777 | 277617 | 239039 | 265703 | 1.33 | 0.70 | down |
| mws0875 | Amino acids and derivatives | 969655 | 1043519 | 1170166 | 731083 | 645914 | 862641 | 1.31 | 0.70 | down |
| Lmhp002031 | Amino acids and derivatives | 96833 | 100400 | 104762 | 55822 | 73053 | 85546 | 1.22 | 0.71 | down |
| Zmmp001429 | Amino acids and derivatives | 48808 | 44518 | 34855 | 32695 | 33302 | 35055 | 1.12 | 0.79 | down |
| pme2914 | Amino acids and derivatives | 446291 | 412128 | 493662 | 330477 | 367474 | 381966 | 1.28 | 0.80 | down |
| Rfmb318 | Amino acids and derivatives | 2409787 | 2429798 | 2310868 | 2178150 | 1833097 | 2144042 | 1.20 | 0.86 | down |
| pmb2591 | Amino acids and derivatives | 295656 | 306075 | 298152 | 296535 | 243168 | 239359 | 1.10 | 0.87 | down |
| Zmzn000113 | Amino acids and derivatives | 6987553 | 7059015 | 7606204 | 5900627 | 7028527 | 5863522 | 1.12 | 0.87 | down |
| Zbqn002253 | Amino acids and derivatives | 2127246 | 2097575 | 2063097 | 2042147 | 2046818 | 2040382 | 1.23 | 0.97 | down |
| pme3033 | Amino acids and derivatives | 4474209 | 3894582 | 3795713 | 4871572 | 4761618 | 4413629 | 1.16 | 1.15 | up |
| Wcdp000380 | Amino acids and derivatives | 5796266 | 5622757 | 6424518 | 7136694 | 6752361 | 6725174 | 1.28 | 1.16 | up |
| pmb2673 | Amino acids and derivatives | 42611 | 42621 | 44771 | 48915 | 47908 | 53644 | 1.33 | 1.16 | up |
| MWS4471 | Amino acids and derivatives | 370734 | 476136 | 435069 | 463798 | 491321 | 540383 | 1.02 | 1.17 | up |
| pme2617 | Amino acids and derivatives | 2632231 | 2921303 | 2874937 | 3079426 | 3533558 | 3303911 | 1.27 | 1.18 | up |
| ML10181668 | Amino acids and derivatives | 2121548 | 1916144 | 1926364 | 2511186 | 2033926 | 2477506 | 1.09 | 1.18 | up |
| MWS4309 | Amino acids and derivatives | 115816 | 99988 | 87754 | 117850 | 114924 | 126077 | 1.07 | 1.18 | up |
| MWSmce119 | Amino acids and derivatives | 7855596 | 8668232 | 9931324 | 9936552 | 10733495 | 10811898 | 1.16 | 1.19 | up |
| MWSmce585 | Amino acids and derivatives | 3058219 | 3054899 | 3303783 | 4072922 | 3282316 | 3979858 | 1.17 | 1.20 | up |
| Yalp000545 | Amino acids and derivatives | 2120467 | 2206210 | 2075557 | 2961410 | 2390461 | 2591103 | 1.28 | 1.24 | up |
| mws0582 | Amino acids and derivatives | 1190137 | 1060172 | 1224208 | 1193443 | 1496587 | 1686185 | 1.06 | 1.26 | up |
| mws0254 | Amino acids and derivatives | 3102273 | 3337433 | 3320981 | 3850976 | 4284107 | 4197549 | 1.42 | 1.26 | up |
| pme0021 | Amino acids and derivatives | 3450991 | 3744041 | 3426528 | 4780610 | 4521111 | 4327058 | 1.43 | 1.28 | up |
| mws0263 | Amino acids and derivatives | 286353 | 330012 | 273539 | 442592 | 371242 | 328298 | 1.15 | 1.28 | up |
| MWS0813 | Amino acids and derivatives | 286353 | 330012 | 273539 | 442592 | 371242 | 328298 | 1.15 | 1.28 | up |
| mws0256 | Amino acids and derivatives | 10016174 | 9009753 | 10138894 | 13438880 | 12799502 | 12441290 | 1.43 | 1.33 | up |
| mws1587 | Amino acids and derivatives | 3994719 | 3624613 | 3943206 | 5135190 | 5008548 | 5739600 | 1.43 | 1.37 | up |
| MWSprf117 | Amino acids and derivatives | 83649 | 73582 | 77622 | 107378 | 102738 | 114193 | 1.44 | 1.38 | up |
| MWS04555g | Amino acids and derivatives | 3530390 | 3788443 | 3854455 | 5010914 | 5515806 | 5370778 | 1.46 | 1.42 | up |
| mws0258 | Amino acids and derivatives | 3530390 | 3788443 | 3854455 | 5010914 | 5515806 | 5370778 | 1.46 | 1.42 | up |
| mws0227 | Amino acids and derivatives | 3530390 | 3788443 | 3854455 | 5010914 | 5515806 | 5370778 | 1.46 | 1.42 | up |
| Zmyn000155 | Amino acids and derivatives | 13450511 | 16161726 | 14300382 | 20850105 | 19166109 | 22630759 | 1.39 | 1.43 | up |
| Zbqn004234 | Amino acids and derivatives | 14798 | 26256 | 30253 | 35225 | 35565 | 33777 | 1.05 | 1.47 | up |
| mws4516 | Amino acids and derivatives | 821883 | 689337 | 794689 | 827796 | 1216494 | 1453419 | 1.12 | 1.52 | up |
| Zmmp002946 | Amino acids and derivatives | 322194 | 304263 | 349699 | 466538 | 549747 | 466512 | 1.43 | 1.52 | up |
| mws0250 | Amino acids and derivatives | 7777790 | 8565374 | 8282289 | 13720387 | 11811177 | 13064332 | 1.46 | 1.57 | up |
| Zmdp000972 | Amino acids and derivatives | 4787602 | 3942375 | 4788274 | 7607717 | 6057445 | 7690235 | 1.37 | 1.58 | up |
| Zmmp003443 | Amino acids and derivatives | 245744 | 206486 | 208806 | 344485 | 388255 | 331171 | 1.43 | 1.61 | up |
| MWS04559g | Amino acids and derivatives | 7610298 | 7357297 | 7372810 | 12858116 | 12421140 | 12809565 | 1.50 | 1.70 | up |
| Zmdp001857 | Amino acids and derivatives | 99627 | 121732 | 133538 | 251084 | 209248 | 210711 | 1.43 | 1.89 | up |
| Zazp002547 | Amino acids and derivatives | 25233832 | 36735728 | 29443345 | 58688812 | 60845089 | 64547050 | 1.43 | 2.01 | up |
| mws0282 | Amino acids and derivatives | 3489341 | 3701960 | 3844649 | 7316845 | 8053065 | 8536318 | 1.49 | 2.17 | up |
| MWS1771 | Amino acids and derivatives | 30262 | 12397 | 41104 | 64748 | 55753 | 63228 | 1.17 | 2.19 | up |
| MWSmce056 | Amino acids and derivatives | 4107199 | 4774692 | 4559737 | 11122296 | 10110656 | 9762417 | 1.49 | 2.31 | up |
| Wcfp004977 | Amino acids and derivatives | 61289 | 11096 | 24115 | 136060 | 74714 | 97593 | 1.19 | 3.20 | up |
| Walp007192 | Lipids | 414198 | 2054366 | 876005 | 299406 | 351672 | 181254 | 1.16 | 0.25 | down |
| Zmyn004732 | Lipids | 9427 | 10814 | 6923 | 2269 | 4973 | 3085 | 1.33 | 0.38 | down |
| Walp007190 | Lipids | 3836988 | 9095622 | 3959012 | 2008202 | 2849022 | 1972741 | 1.21 | 0.40 | down |
| pmp001260 | Lipids | 106203 | 76494 | 60294 | 27370 | 37443 | 65477 | 1.11 | 0.54 | down |
| pmf0293 | Lipids | 399397 | 291581 | 275917 | 159311 | 150975 | 242587 | 1.25 | 0.57 | down |
| mws0126 | Lipids | 421412 | 715715 | 354018 | 330140 | 347320 | 202886 | 1.02 | 0.59 | down |
| Walp007043 | Lipids | 12536175 | 10724978 | 10960587 | 7346931 | 7686173 | 7304782 | 1.46 | 0.65 | down |
| pmp001276 | Lipids | 643921 | 631932 | 701994 | 444719 | 430582 | 427206 | 1.48 | 0.66 | down |
| pmp001264 | Lipids | 13690504 | 11470147 | 12813628 | 8675056 | 8945127 | 9102082 | 1.43 | 0.70 | down |
| Waln011524 | Lipids | 135394 | 102707 | 119340 | 109554 | 67360 | 75010 | 1.11 | 0.70 | down |
| mws0396 | Lipids | 561066 | 460358 | 480643 | 364153 | 361527 | 362189 | 1.40 | 0.72 | down |
| Wcdp006929 | Lipids | 98099 | 80452 | 85740 | 61221 | 61705 | 69802 | 1.36 | 0.73 | down |
| mws0367 | Lipids | 2526131 | 2691953 | 2944834 | 2282314 | 2275592 | 1848082 | 1.24 | 0.78 | down |
| mws0366 | Lipids | 2526131 | 2691953 | 2944834 | 2282314 | 2275592 | 1848082 | 1.24 | 0.78 | down |
| pmn001694 | Lipids | 32144 | 35754 | 38905 | 26312 | 30127 | 27699 | 1.30 | 0.79 | down |
| Lmmn004951 | Lipids | 61178 | 73169 | 74244 | 56701 | 61053 | 57208 | 1.19 | 0.84 | down |
| mws1488 | Lipids | 8587 | 9029 | 8108 | 7556 | 6812 | 7463 | 1.31 | 0.85 | down |
| Lmhp011562 | Lipids | 75922 | 76220 | 79960 | 67467 | 60742 | 76393 | 1.03 | 0.88 | down |
| MWS5231 | Lipids | 71755 | 82025 | 84166 | 70975 | 71209 | 72389 | 1.07 | 0.90 | down |
| pmd0132 | Lipids | 37553430 | 39255318 | 38645970 | 38640286 | 40863081 | 41314269 | 1.02 | 1.05 | up |
| pmb0855 | Lipids | 4203156 | 3720803 | 3835384 | 4135565 | 4468816 | 4370878 | 1.13 | 1.10 | up |
| Hmyn007168 | Lipids | 73994 | 74892 | 73323 | 78692 | 88183 | 83438 | 1.30 | 1.13 | up |
| pmp001270 | Lipids | 3417537 | 3465749 | 3271483 | 4140349 | 3706745 | 3763544 | 1.30 | 1.14 | up |
| pmb2260 | Lipids | 200637 | 188370 | 219715 | 222997 | 220886 | 254169 | 1.10 | 1.15 | up |
| pmb0865 | Lipids | 2755652 | 3269311 | 2891786 | 3163483 | 3754990 | 3331607 | 1.04 | 1.15 | up |
| pmp001251 | Lipids | 15255460 | 15058863 | 16427671 | 18594401 | 17658751 | 18041628 | 1.39 | 1.16 | up |
| pmb2221 | Lipids | 11791043 | 11411281 | 11243301 | 13732489 | 13553141 | 12895371 | 1.43 | 1.17 | up |
| Lmhp008763 | Lipids | 774968 | 920524 | 835285 | 965397 | 928888 | 1109841 | 1.14 | 1.19 | up |
| pmp001273 | Lipids | 16496731 | 16782500 | 15015251 | 19241889 | 19327732 | 19045906 | 1.40 | 1.19 | up |
| Wcfp008029 | Lipids | 450328 | 464443 | 448438 | 582992 | 513655 | 559980 | 1.39 | 1.22 | up |
| MWSslk133 | Lipids | 35232 | 36285 | 36362 | 47216 | 41497 | 43105 | 1.39 | 1.22 | up |
| Lmhp010162 | Lipids | 96983 | 116920 | 93290 | 142856 | 117305 | 122198 | 1.15 | 1.24 | up |
| pmb2319 | Lipids | 435778 | 440658 | 447742 | 590870 | 601096 | 600846 | 1.50 | 1.35 | up |
| pmd0136 | Lipids | 410911 | 233963 | 260977 | 427440 | 434337 | 373919 | 1.03 | 1.36 | up |
| Lmhp008885 | Lipids | 167142 | 115028 | 151024 | 197220 | 180496 | 219811 | 1.20 | 1.38 | up |
| Lmhp009187 | Lipids | 127083 | 144947 | 140088 | 196342 | 211241 | 161522 | 1.31 | 1.38 | up |
| Waln010192 | Lipids | 18250 | 17341 | 15055 | 25428 | 25734 | 18880 | 1.21 | 1.38 | up |
| Lmhp008718 | Lipids | 93848 | 96497 | 77428 | 115289 | 125364 | 131306 | 1.35 | 1.39 | up |
| Waln010743 | Lipids | 55936 | 70947 | 74025 | 83671 | 122664 | 87602 | 1.17 | 1.46 | up |
| pmb0880 | Lipids | 94667 | 67825 | 69279 | 98748 | 96805 | 152488 | 1.10 | 1.50 | up |
| Waln011009 | Lipids | 69417 | 56568 | 72015 | 101617 | 95685 | 100976 | 1.41 | 1.51 | up |
| ML10195036 | Lipids | 1376352 | 1734478 | 1266965 | 2342770 | 2205489 | 2166107 | 1.37 | 1.53 | up |
| Zbfn007626 | Lipids | 1287773 | 1737325 | 1728304 | 2255248 | 2607638 | 2575215 | 1.35 | 1.56 | up |
| Lmhp008440 | Lipids | 20541 | 32053 | 20875 | 45736 | 36870 | 38469 | 1.28 | 1.65 | up |
| pmb0863 | Lipids | 340566 | 317684 | 299793 | 501951 | 592880 | 528634 | 1.46 | 1.69 | up |
| pma1303 | Lipids | 326756 | 390403 | 337598 | 626175 | 637612 | 563547 | 1.46 | 1.73 | up |
| MWS0552 | Lipids | 822139 | 842998 | 796973 | 2449953 | 1773594 | 1564623 | 1.43 | 2.35 | up |
| Zajn009321 | Terpenoids | 5640 | 7635 | 7131 | 2259 | 1928 | 2645 | 1.46 | 0.33 | down |
| Labp005299 | Terpenoids | 81695 | 68547 | 77539 | 11670 | 21146 | 45311 | 1.26 | 0.34 | down |
| Wmqn000820 | Terpenoids | 2339015 | 2711594 | 2894408 | 1029482 | 804669 | 1012520 | 1.47 | 0.36 | down |
| Zjyp102926 | Terpenoids | 515937 | 469238 | 467405 | 179829 | 255950 | 232014 | 1.45 | 0.46 | down |
| Qmyp101422 | Terpenoids | 979164 | 919598 | 809922 | 393006 | 572914 | 373655 | 1.39 | 0.49 | down |
| Cmbn005546 | Terpenoids | 86720 | 91374 | 85537 | 54145 | 32979 | 49871 | 1.36 | 0.52 | down |
| Lmjp002718 | Terpenoids | 3858054 | 3407014 | 3465278 | 1783417 | 1993465 | 2035495 | 1.48 | 0.54 | down |
| Wagp004279 | Terpenoids | 887816 | 701750 | 577124 | 386884 | 342419 | 465794 | 1.33 | 0.55 | down |
| Zmsn003589 | Terpenoids | 1326850 | 1416102 | 1071655 | 945395 | 740634 | 646669 | 1.31 | 0.61 | down |
| MWSmce096 | Terpenoids | 317076 | 324970 | 315775 | 194490 | 197837 | 207175 | 1.49 | 0.63 | down |
| Ljmp121502 | Terpenoids | 172249 | 175734 | 166809 | 101990 | 109871 | 113025 | 1.48 | 0.63 | down |
| Lhlp111516 | Terpenoids | 149996 | 137336 | 139253 | 89270 | 94575 | 92716 | 1.48 | 0.65 | down |
| Wbsn001677 | Terpenoids | 296082 | 267210 | 317035 | 232705 | 179580 | 163656 | 1.32 | 0.65 | down |
| Qmwp111611 | Terpenoids | 234837 | 206746 | 184313 | 126536 | 169627 | 118383 | 1.27 | 0.66 | down |
| Lacp005602 | Terpenoids | 33064 | 40101 | 52216 | 25005 | 35951 | 24624 | 1.09 | 0.68 | down |
| Yajn003045 | Terpenoids | 948326 | 905212 | 685646 | 646934 | 469497 | 626518 | 1.19 | 0.69 | down |
| Lakn004098 | Terpenoids | 659960 | 733690 | 756969 | 557521 | 548457 | 508598 | 1.42 | 0.75 | down |
| Qmyp101125 | Terpenoids | 140097 | 136421 | 106591 | 89287 | 83889 | 115449 | 1.11 | 0.75 | down |
| Qmyp101114 | Terpenoids | 342066 | 278134 | 309364 | 244701 | 258476 | 216743 | 1.27 | 0.77 | down |
| Wbmn002291 | Terpenoids | 29714 | 32423 | 34762 | 28248 | 20509 | 26727 | 1.14 | 0.78 | down |
| Wcop004851 | Terpenoids | 34148384 | 39544204 | 35059991 | 28860710 | 29409828 | 29745403 | 1.37 | 0.81 | down |
| Saln000462 | Terpenoids | 13435948 | 16517744 | 16279667 | 13894754 | 12743439 | 12965731 | 1.09 | 0.86 | down |
| Saln003762 | Terpenoids | 2408190 | 2513122 | 2604641 | 1985617 | 2155030 | 2440991 | 1.10 | 0.87 | down |
| Cmpp004946 | Terpenoids | 6705445 | 6670885 | 6616593 | 6530194 | 5889331 | 5891981 | 1.18 | 0.92 | down |
| Lhsp101904 | Terpenoids | 2287100 | 2202424 | 2334521 | 2057481 | 2243211 | 2050470 | 1.09 | 0.93 | down |
| pmf0085 | Terpenoids | 298651 | 300357 | 319270 | 326540 | 333886 | 333805 | 1.31 | 1.08 | up |
| Cmpp004143 | Terpenoids | 2025459 | 1960099 | 2197722 | 2323840 | 2302013 | 2152848 | 1.11 | 1.10 | up |
| Cmpp004617 | Terpenoids | 2704333 | 2337975 | 2260731 | 2774636 | 2688408 | 2986954 | 1.14 | 1.16 | up |
| Sayp010368 | Terpenoids | 226322 | 234750 | 216127 | 285015 | 266511 | 248214 | 1.31 | 1.18 | up |
| Wmxn004282 | Terpenoids | 6587151 | 6431853 | 6267314 | 7555743 | 7473508 | 7990020 | 1.44 | 1.19 | up |
| pmp001046 | Terpenoids | 157637 | 128300 | 134462 | 198555 | 177153 | 160946 | 1.22 | 1.28 | up |
| pmp000529 | Terpenoids | 1852783 | 1959195 | 1721124 | 2520584 | 2594859 | 2248164 | 1.39 | 1.33 | up |
| pmp001090 | Terpenoids | 67900 | 68129 | 70421 | 110995 | 84676 | 89161 | 1.33 | 1.38 | up |
| Yajp005210 | Terpenoids | 233424 | 239835 | 301241 | 393167 | 306858 | 373345 | 1.24 | 1.39 | up |
| Lcmp123017 | Terpenoids | 213850 | 328067 | 194721 | 403887 | 356564 | 278335 | 1.02 | 1.41 | up |
| Lmzp003694 | Terpenoids | 4870873 | 5239566 | 5014259 | 6885260 | 7478696 | 7931469 | 1.46 | 1.47 | up |
| Hmcn000773 | Terpenoids | 18259 | 10689 | 14260 | 21787 | 24827 | 17354 | 1.10 | 1.48 | up |
| Yalp005912 | Terpenoids | 1298659 | 1229032 | 1149201 | 1977688 | 1836233 | 1818661 | 1.47 | 1.53 | up |
| Lwhp011003 | Terpenoids | 306714 | 258168 | 240500 | 486674 | 397879 | 383756 | 1.37 | 1.57 | up |
| MWSHC2097 | Terpenoids | 1488247 | 1299235 | 1492913 | 2477331 | 2135731 | 2238122 | 1.45 | 1.60 | up |
| Qmgp082703 | Terpenoids | 18618745 | 15858513 | 19031776 | 27916605 | 27157313 | 30604208 | 1.44 | 1.60 | up |
| Sahp005646 | Terpenoids | 247793 | 218820 | 219620 | 370877 | 374163 | 363458 | 1.48 | 1.62 | up |
| Cmmp006152 | Terpenoids | 3124650 | 3198200 | 3366804 | 5573314 | 5403251 | 5547665 | 1.49 | 1.71 | up |
| Lwhp010603 | Terpenoids | 74334 | 97250 | 90181 | 139205 | 146653 | 172466 | 1.41 | 1.75 | up |
| Lmzn003560 | Terpenoids | 191587 | 176434 | 188400 | 395478 | 362769 | 379455 | 1.49 | 2.04 | up |
| MWSHY0091 | Terpenoids | 45909 | 53140 | 52190 | 109433 | 126606 | 89171 | 1.44 | 2.15 | up |
| MWSmce536 | Organic acids | 55692 | 61942 | 57694 | 35258 | 29345 | 30841 | 1.47 | 0.54 | down |
| pme3009 | Organic acids | 841455 | 889703 | 795115 | 541305 | 452928 | 542727 | 1.45 | 0.61 | down |
| Zmgn001448 | Organic acids | 98818 | 90783 | 106222 | 65630 | 58698 | 61577 | 1.46 | 0.63 | down |
| Lmbn001754 | Organic acids | 98818 | 90783 | 106222 | 65630 | 58698 | 61577 | 1.46 | 0.63 | down |
| Lmbn001288 | Organic acids | 474701 | 557718 | 589968 | 341793 | 300175 | 425303 | 1.30 | 0.66 | down |
| Lmbn001364 | Organic acids | 444311 | 461200 | 473107 | 329752 | 318272 | 301822 | 1.48 | 0.69 | down |
| Lmmn002164 | Organic acids | 439244 | 470336 | 559060 | 389441 | 324702 | 313247 | 1.31 | 0.70 | down |
| mws0473 | Organic acids | 439244 | 470336 | 559060 | 389441 | 324702 | 313247 | 1.31 | 0.70 | down |
| Lmmn000806 | Organic acids | 439244 | 470336 | 559060 | 389441 | 324702 | 313247 | 1.31 | 0.70 | down |
| Zbdp002392 | Organic acids | 941820 | 932605 | 695679 | 500823 | 501593 | 829696 | 1.01 | 0.71 | down |
| mws0192 | Organic acids | 9586203 | 9205682 | 9946324 | 6071841 | 7591340 | 7614279 | 1.34 | 0.74 | down |
| Lmsn015919 | Organic acids | 6077 | 4177 | 4760 | 4304 | 3416 | 3491 | 1.10 | 0.75 | down |
| pme3096 | Organic acids | 354482 | 372433 | 350532 | 305400 | 223223 | 302091 | 1.19 | 0.77 | down |
| pmb3101 | Organic acids | 88240 | 80373 | 78598 | 63417 | 64331 | 62956 | 1.45 | 0.77 | down |
| Lmtn004049 | Organic acids | 1733045 | 1679732 | 1694335 | 1339747 | 1266220 | 1340100 | 1.48 | 0.77 | down |
| mws0470 | Organic acids | 9607606 | 9004050 | 8710626 | 6325216 | 7576330 | 7259923 | 1.35 | 0.77 | down |
| mws0376 | Organic acids | 1819654 | 1502480 | 1824122 | 1232155 | 1567801 | 1373112 | 1.12 | 0.81 | down |
| Rfmb320 | Organic acids | 2409787 | 2429798 | 2310868 | 2178150 | 1833097 | 2144042 | 1.20 | 0.86 | down |
| Wayn001528 | Organic acids | 31538034 | 31254210 | 33248607 | 34006899 | 34990779 | 36764352 | 1.28 | 1.10 | up |
| Wayn000884 | Organic acids | 19895286 | 20156589 | 18338289 | 19622225 | 22396869 | 24099608 | 1.01 | 1.13 | up |
| Wasn003197 | Organic acids | 75456 | 98963 | 80358 | 105473 | 94500 | 99384 | 1.03 | 1.17 | up |
| MWS2040 | Organic acids | 376632 | 369009 | 419736 | 437348 | 486528 | 460722 | 1.30 | 1.19 | up |
| Lcsn003224 | Organic acids | 1172081 | 1150345 | 1317178 | 1349297 | 1672899 | 1323578 | 1.06 | 1.19 | up |
| pme3017 | Organic acids | 3525221 | 3926351 | 3451404 | 4534635 | 4132074 | 4433060 | 1.33 | 1.20 | up |
| Wayn000918 | Organic acids | 5855317 | 7649975 | 8152051 | 8546513 | 8783488 | 8813261 | 1.05 | 1.21 | up |
| Lmyn002403 | Organic acids | 1010224 | 1138527 | 1036805 | 1168138 | 1501652 | 1387426 | 1.23 | 1.27 | up |
| MWS0274 | Organic acids | 127212 | 138039 | 128880 | 162252 | 197098 | 177088 | 1.39 | 1.36 | up |
| Wayn002279 | Organic acids | 82318 | 116769 | 105874 | 113689 | 152161 | 152818 | 1.12 | 1.37 | up |
| MWS0811 | Organic acids | 2146391 | 1707564 | 1931410 | 3098825 | 2809075 | 2494603 | 1.35 | 1.45 | up |
| pme0274 | Organic acids | 3313628 | 3974380 | 3640481 | 4639576 | 5237217 | 6101297 | 1.34 | 1.46 | up |
| mws0147 | Organic acids | 169752 | 197656 | 212823 | 333949 | 290278 | 235583 | 1.27 | 1.48 | up |
| Zbqp000579 | Organic acids | 532521 | 518604 | 635025 | 668887 | 1126761 | 703959 | 1.08 | 1.48 | up |
| Lmgn002091 | Organic acids | 51264 | 68023 | 87121 | 110503 | 111615 | 100868 | 1.25 | 1.56 | up |
| pme3186 | Organic acids | 1210903 | 1607575 | 1387116 | 2190891 | 2488992 | 2175025 | 1.40 | 1.63 | up |
| Lmgn000224 | Organic acids | 26591 | 21030 | 31038 | 45512 | 50166 | 36301 | 1.30 | 1.68 | up |
| mws0874 | Nucleotides and derivatives | 23288 | 38345 | 55015 | 10987 | 14231 | 18113 | 1.28 | 0.37 | down |
| mws0976 | Nucleotides and derivatives | 125309 | 119796 | 137597 | 104637 | 99458 | 69166 | 1.19 | 0.71 | down |
| MWS5083 | Nucleotides and derivatives | 514490 | 524136 | 481403 | 381938 | 412826 | 324968 | 1.35 | 0.74 | down |
| ML10180524 | Nucleotides and derivatives | 485169 | 556632 | 622808 | 452561 | 375759 | 442571 | 1.23 | 0.76 | down |
| pmb0981 | Nucleotides and derivatives | 3665144 | 4357683 | 4168885 | 3328174 | 2964964 | 3258561 | 1.33 | 0.78 | down |
| MWSmce116 | Nucleotides and derivatives | 151920 | 168295 | 155568 | 134640 | 141834 | 100695 | 1.11 | 0.79 | down |
| pme3337 | Nucleotides and derivatives | 2557468 | 2779028 | 2715050 | 2054370 | 2509699 | 2270651 | 1.20 | 0.85 | down |
| pme1474 | Nucleotides and derivatives | 14707459 | 13969813 | 13487264 | 13445798 | 12940092 | 12591810 | 1.17 | 0.92 | down |
| MW0154492 | Nucleotides and derivatives | 983476 | 1007005 | 1034041 | 1043633 | 1068154 | 1035990 | 1.14 | 1.04 | up |
| Zmsp001773 | Nucleotides and derivatives | 9206931 | 8511090 | 7902376 | 9193760 | 9220609 | 9753953 | 1.06 | 1.10 | up |
| pme0152 | Nucleotides and derivatives | 237701 | 242928 | 247630 | 260915 | 270611 | 300460 | 1.25 | 1.14 | up |
| MW0103352 | Nucleotides and derivatives | 135275 | 121574 | 132667 | 163871 | 154920 | 130545 | 1.01 | 1.15 | up |
| mws0847 | Nucleotides and derivatives | 3314277 | 2752958 | 2741454 | 4014186 | 3427717 | 3654145 | 1.25 | 1.26 | up |
| pme1178 | Nucleotides and derivatives | 1245213 | 1684709 | 1409613 | 2100775 | 1742766 | 1658381 | 1.09 | 1.27 | up |
| Wayn000421 | Nucleotides and derivatives | 58569 | 50907 | 52841 | 55668 | 74004 | 79945 | 1.08 | 1.29 | up |
| pmb2507 | Nucleotides and derivatives | 236119 | 273602 | 245358 | 361636 | 276015 | 367451 | 1.21 | 1.33 | up |
| pmc0281 | Nucleotides and derivatives | 301843 | 255311 | 238013 | 343550 | 342084 | 385394 | 1.32 | 1.35 | up |
| MWSmce295 | Nucleotides and derivatives | 234735 | 398661 | 311113 | 395890 | 504479 | 401260 | 1.05 | 1.38 | up |
| MWS4525 | Nucleotides and derivatives | 100344 | 107128 | 102811 | 166138 | 123301 | 142410 | 1.32 | 1.39 | up |
| mws0863 | Nucleotides and derivatives | 183714 | 183937 | 219660 | 264473 | 291202 | 318198 | 1.39 | 1.49 | up |
| pmb0530 | Nucleotides and derivatives | 163124 | 155696 | 53415 | 163340 | 307558 | 297708 | 1.04 | 2.06 | up |
| Wayp001024 | Nucleotides and derivatives | 23017 | 18371 | 16601 | 61206 | 68567 | 57513 | 1.48 | 3.23 | up |
| Wbjp004531 | Quinones | 498045 | 549539 | 455544 | 314101 | 327452 | 297045 | 1.45 | 0.62 | down |
| Lmqp003022 | Quinones | 978821 | 1214357 | 1133772 | 760474 | 720604 | 666159 | 1.42 | 0.65 | down |
| Zbzn008048 | Quinones | 1692797 | 1795872 | 2064612 | 1717858 | 1445595 | 1595583 | 1.06 | 0.86 | down |
| Wcfn008271 | Quinones | 15789643 | 14839349 | 15481959 | 15925787 | 16496738 | 16659020 | 1.21 | 1.06 | up |
| Lsj214147 | Quinones | 803752 | 822053 | 485874 | 1186627 | 1199042 | 1114129 | 1.26 | 1.66 | up |
| Zmtp102902 | Quinones | 1592294 | 993946 | 1295253 | 2527500 | 1862249 | 2177132 | 1.28 | 1.69 | up |
| Zmdn011161 | Quinones | 733803 | 787307 | 882385 | 2249535 | 1595529 | 1600609 | 1.43 | 2.27 | up |

Note: FC: Fold Change

Table S3. Number of reads after filtering rRNA and low quality

| Sample | Total | Mapped reads (%) | Unique Mapped reads (%) | Multi Mapped reads (%) | Q20 (%) | Q30 (%) |
| --- | --- | --- | --- | --- | --- | --- |
| SD-PH0d-1 | 39643132 | 37442019(94.45%) | 36529646(92.15%) | 912373(2.30%) | 99.95 | 97.05 |
| SD-PH0d-2 | 48142322 | 45433753(94.37%) | 44235200(91.88%) | 1198553(2.49%) | 99.97 | 97.37 |
| SD-PH0d-3 | 43053782 | 40648663(94.41%) | 39652145(92.10%) | 996518(2.31%) | 99.95 | 97.07 |
| SD-PH30d-1 | 44678496 | 42060666(94.14%) | 40720716(91.14%) | 1339950(3.00%) | 99.96 | 97.21 |
| SD-PH30d-2 | 42555992 | 40119777(94.28%) | 38966982(91.57%) | 1152795(2.71%) | 99.96 | 97.11 |
| SD-PH30d-3 | 45180810 | 42511279(94.09%) | 41240351(91.28%) | 1270928(2.81%) | 99.95 | 97.09 |
| SD-PH50d-1 | 42821778 | 40295704(94.10%) | 38985537(91.04%) | 1310167(3.06%) | 99.96 | 97.17 |
| SD-PH50d-2 | 49867474 | 46627631(93.50%) | 44659336(89.56%) | 1968295(3.95%) | 99.95 | 97.08 |
| SD-PH50d-3 | 41559084 | 39160517(94.23%) | 37418248(90.04%) | 1742269(4.19%) | 99.96 | 97.14 |
| CK-PH0d-1 | 45019486 | 42270741(93.89%) | 41061398(91.21%) | 1209343(2.69%) | 99.98 | 97.79 |
| CK-PH0d-2 | 44827942 | 41995016(93.68%) | 40834842(91.09%) | 1160174(2.59%) | 99.98 | 97.69 |
| CK-PH0d-3 | 48565188 | 45638493(93.97%) | 43907269(90.41%) | 1731224(3.56%) | 99.98 | 97.76 |
| CK-PH30d-1 | 50023204 | 46975104(93.91%) | 45418217(90.79%) | 1556887(3.11%) | 99.97 | 97.73 |
| CK-PH30d-2 | 48023012 | 45075659(93.86%) | 43487805(90.56%) | 1587854(3.31%) | 99.97 | 97.81 |
| CK-PH30d-3 | 46703292 | 43829600(93.85%) | 42288822(90.55%) | 1540778(3.30%) | 99.97 | 97.66 |
| CK-PH50d-1 | 48265830 | 45258977(93.77%) | 43878494(90.91%) | 1380483(2.86%) | 99.97 | 97.75 |
| CK-PH50d-2 | 47752962 | 44826993(93.87%) | 42961582(89.97%) | 1865411(3.91%) | 99.96 | 97.37 |
| CK-PH50d-3 | 50262874 | 47132674(93.77%) | 45440320(90.41%) | 1692354(3.37%) | 99.97 | 97.82 |


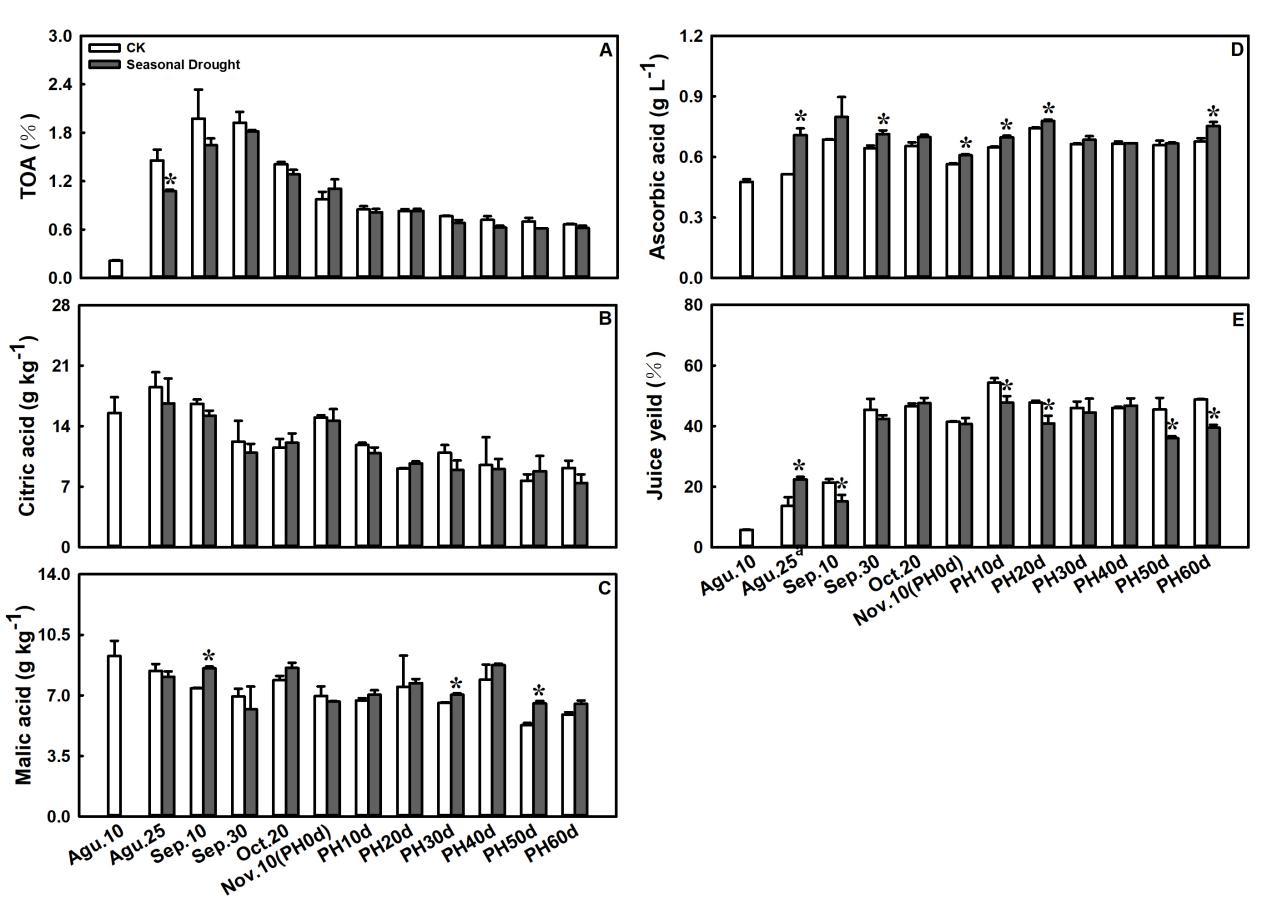


Fig. S1 Effects of drought treatment on the content of total organic acid (A), citric acid (B), malic acid (C), ascorbic acid (D) and juice yield (E) during fruit ripening and postharvest storage period. Total organic acid: TOA. Error bars represent the standard deviations of the mean in three replicates, and for each stage, * stand for significant differences between seasonal drought treatment and control at *p* < 0.05.


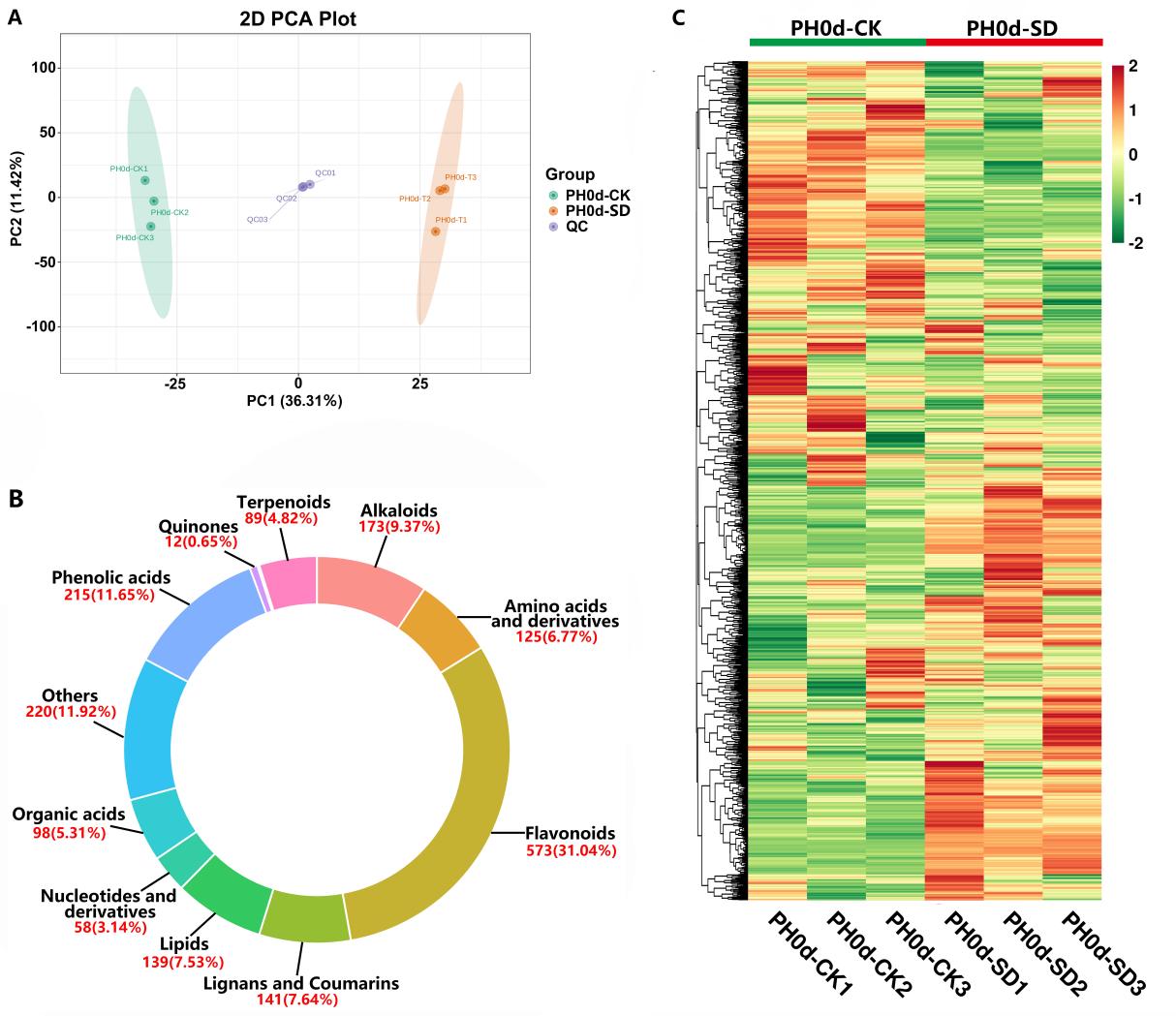


Fig. S2 Widely targeted metabolome analysis of the mature fruit juice sacs between control and seasonal drought treatment. (A) Principal component analysis (PCA) scores of mass spectrometry data of samples from different groups. (B) Biochemical categories of the metabolites identified from fruit juice sacs of blood orange fruit. (C) Cluster heat map of metabolites from samples of the control and seasonal drought treatment. The color indicates the accumulation level of each metabolite, from low (green) to high (red). The data was standardized by Zscore. ‘PH0d-CK’ and ‘PH0d-SD’ means samples from control and seasonal drought treatment blood orange fruit at mature stage, respectively; ‘QC’ means quality control samples.


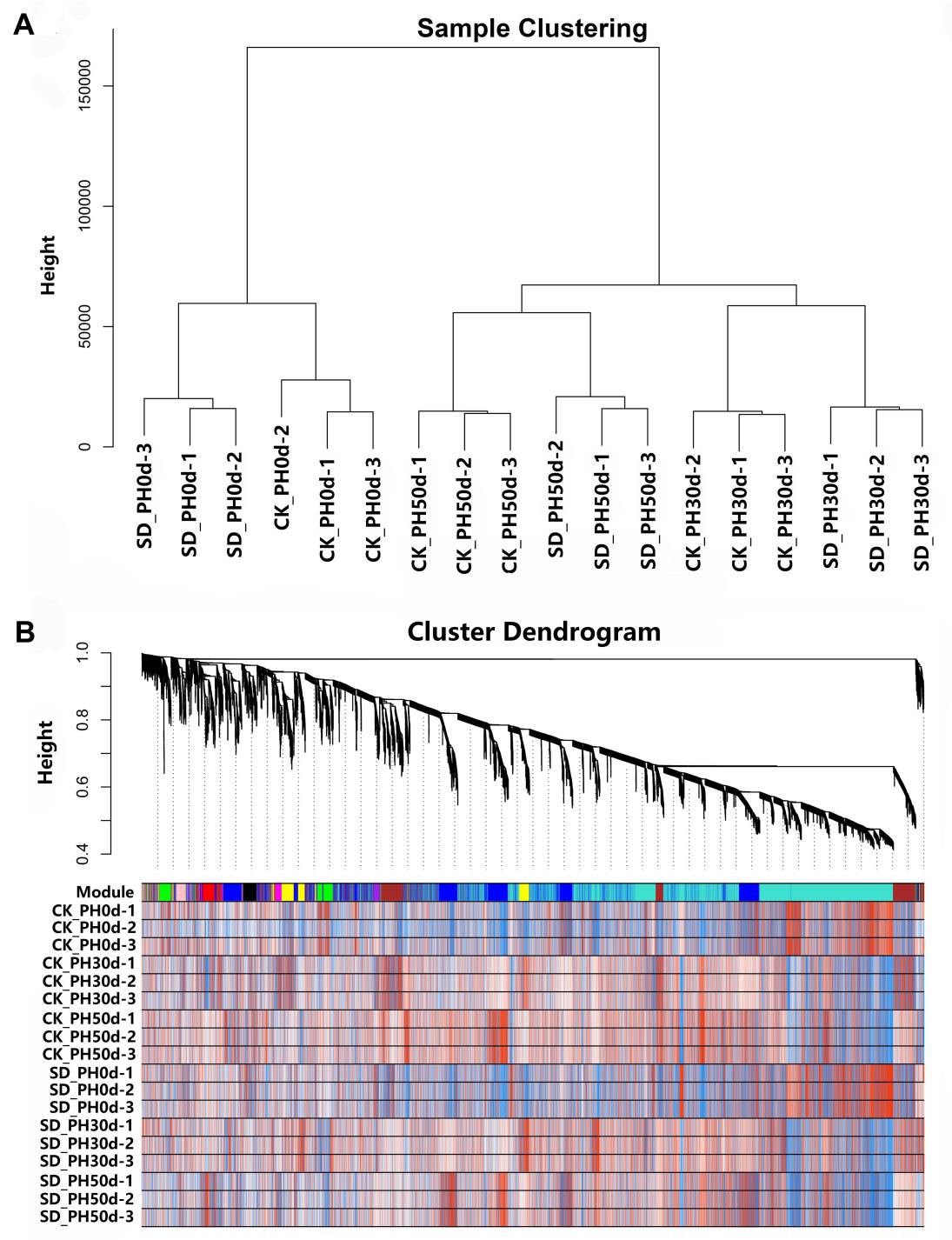


Fig. S3 Construction of weighted gene coexpression network. (A) Sample clustering and (B) modules of coexpression genes showed by hierarchical clustering.
